# Development and evaluation of an advanced National Air Quality Forecasting Capability using the NOAA Global Forecast System version 16

**DOI:** 10.5194/gmd-15-3281-2022

**Published:** 2022-04-21

**Authors:** Patrick C. Campbell, Youhua Tang, Pius Lee, Barry Baker, Daniel Tong, Rick Saylor, Ariel Stein, Jianping Huang, Ho-Chun Huang, Edward Strobach, Jeff McQueen, Li Pan, Ivanka Stajner, Jamese Sims, Jose Tirado-Delgado, Youngsun Jung, Fanglin Yang, Tanya L. Spero, Robert C. Gilliam

**Affiliations:** 1 NOAA Air Resources Laboratory (ARL), College Park, MD, USA; 2 Center for Spatial Information Science and Systems, George Mason University, Fairfax, VA, USA; 3 NOAA National Centers for Environmental Prediction (NCEP), College Park, MD, USA; 4 I.M. Systems Group Inc., Rockville, MD, USA; 5 NOAA NWS/STI, College Park, MD, USA; 6 Eastern Research Group, Inc. (ERG), College Park, MD, USA; 7 US Environmental Protection Agency, Research Triangle Park, NC, USA

## Abstract

A new dynamical core, known as the Finite-Volume Cubed-Sphere (FV3) and developed at both NASA and NOAA, is used in NOAA’s Global Forecast System (GFS) and in limited-area models for regional weather and air quality applications. NOAA has also upgraded the operational FV3GFS to version 16 (GFSv16), which includes a number of significant developmental advances to the model configuration, data assimilation, and underlying model physics, particularly for atmospheric composition to weather feedback. Concurrent with the GFSv16 upgrade, we couple the GFSv16 with the Community Multiscale Air Quality (CMAQ) model to form an advanced version of the National Air Quality Forecasting Capability (NAQFC) that will continue to protect human and ecosystem health in the US. Here we describe the development of the FV3GFSv16 coupling with a “state-of-the-science” CMAQ model version 5.3.1. The GFS–CMAQ coupling is made possible by the seminal version of the NOAA-EPA Atmosphere–Chemistry Coupler (NACC), which became a major piece of the next operational NAQFC system (i.e., NACC-CMAQ) on 20 July 2021. NACC-CMAQ has a number of scientific advancements that include satellite-based data acquisition technology to improve land cover and soil characteristics and inline wildfire smoke and dust predictions that are vital to predictions of fine particulate matter (PM_2.5_) concentrations during hazardous events affecting society, ecosystems, and human health. The GFS-driven NACC-CMAQ model has significantly different meteorological and chemical predictions compared to the previous operational NAQFC, where evaluation of NACC-CMAQ shows generally improved near-surface ozone and PM_2.5_ predictions and diurnal patterns, both of which are extended to a 72 h (3 d) forecast with this system.

## 1 Introduction

Air quality is defined as the degree to which the ambient air is free of pollutants – which are either directly emitted into the atmosphere (primary air pollutants) or formed within the atmosphere itself (secondary air pollutants) – that cause degradation to human health, visibility, and/or ecological systems (WHO, 2006). Air quality is as ubiquitous and important as weather impacts, where outdoor air pollution is globally responsible for ~ 4.2 million early deaths each year (https://www.who.int/health-topics/air-pollution#tab=tab_1, last access: 5 April 2022). To put this into perspective, this is over 3 times the number of people who die from HIV/AIDS and over 8 times the number of homicides each year (2017 Global Burden of Disease Study: https://www.thelancet.com/gbd, last access: 5 April 2022). Air pollution is costly and leads to huge economic damage (Landrigan et al., 2018). There are also disproportionate impacts of air pollution across poorer people and some racial and ethnic groups, who are among those who often face higher exposure and potential responses to pollutants (Institute of Medicine, 1999; American Lung Association, 2001; O’Neil et al., 2003; Finkelstein et al., 2003; Zeka et al., 2006).

Air pollutants are composed of both gaseous and particulate species, which under prolonged exposure can cause non-carcinogenic (Lee et al., 2014) and/or carcinogenic adverse health effects (Demetirou and Vineis, 2015). High ground-level ozone (O_3_) concentrations (i.e., smog), for example, can lead to decreased lung function and cause respiratory symptoms. These symptoms are particularly dangerous for sensitive groups such as young children, the elderly, and those with preexisting conditions that include asthma, chronic obstructive pulmonary disease (COPD), lung cancer, and respiratory infection (Kar Kurt et al., 2016).

To protect against the health and environmental impacts of air pollution, world agencies have developed regulations and standards on the allowable amount of primary and secondary air pollution measured at different spatiotemporal scales (e.g., seconds to months and local to global scales), which largely depend on the atmospheric lifetime of specific air components (WHO, 2005, 2010). Typically, the world’s most extreme air pollution occurs near global megacities where population density is highest (Marlier et al., 2016). Rapid economic growth in China, for example, has led to extremely high air pollution levels over the past decade (Zhou et al., 2017; Liu and Wang, 2020), necessitating significant efforts to implement air pollution prevention and control plans (Chinese State Council, 2013; Zhao et al., 2017). The US Environmental Protection Agency (EPA) defines ambient concentration limits for primary pollutants such as sulfur dioxide (SO_2_), oxides of nitrogen (NO_*x*_=NO+NO_2_), carbon monoxide (CO), lead (Pb), and total (carbonaceous and non-carbonaceous) particulate matter (PM). Other important primary pollutants include total volatile organic compounds (VOCs), which have many sources (both natural and anthropogenic) and serve as vital precursor gases to secondary pollutants such as ground-level O_3_ and the formation of fine particulate matter with an aerodynamic diameter of less than 2.5 μm (PM_2.5_). Ground-level O_3_ and PM_2.5_ are two of the six US EPA “criteria pollutants” that are regulated for their concentrations, exposure level, and health impacts. This is largely because there is a relatively mature understanding of their sources, formation, and characteristics (e.g., Sillman et al., 1990; Sillman, 1995, 1999; Pinder et al., 2008; Kim et al., 2011a, b; Zhang et al., 2009a, b; Campbell et al., 2015; Karamchandani et al., 2017). There is also a widespread ability to compare observed and simulated ambient ozone concentrations over both short-term (McKeen et al., 2005, 2007, 2009) and dynamic long-term periods (e.g., Astitha et al., 2017), which has helped lead to an understanding of their well-attributable health impacts (e.g., WHO, 2006; Sun et al., 2015; Zhang et al., 2018).

To address prolific air pollution concerns in the US during the 1950s–1960s, the first development and application of real-time air quality forecast (RT-AQF) models began in the 1970s–1980s (i.e., the first- and second-generation air quality models) coincident with the establishment of the US EPA by President Nixon. Initially the models were based on empirical approaches and statistical models (Zhang et al., 2012a); however, by the 1990s and early 2000s, RT-AQF models underwent a significant evolution and evolved to more complex 3-D numerical air quality models (third and fourth-generation air quality models). These RT-AQF models involved more sophisticated techniques, including increasingly complex parameterizations and chemistry, bias-correction methods and data fusion, chemical data assimilation, and hybrid statistical or numerical methods with artificial intelligence and machine learning algorithms to improve RT-AQF model efficiency and predictions (Zhang et al., 2012b; Bai et al., 2018). RT-AQF models have become vital tools to improve our understanding and prediction of how air pollutants form, disperse, and deposit to the surface and are used by local health and air managers to assess the air quality conditions to make informed decisions on mitigation measures to reduce public exposure.

To address the nation’s need for reducing the adverse health effects of air pollution and associated costly medical expenses, in 2002 Congress addressed the National Oceanic and Atmospheric Administration (NOAA) to provide National AQF guidance (H.R. Energy Policy Act of 2002 – Senate Amendment S. 517, SA1383, Forecasts and Warnings). A joint project emerged from this amendment between NOAA and the EPA to develop and establish the initial phase of a RT-AQF system, which consisted of the coupled NOAA’s ETA meteorological model (Black, 1994; Rogers et al., 1996) with EPA’s Models-3 Community Multiscale Air Quality (CMAQ) model (Byun and Ching, 1999; Byun and Schere, 2006). This “offline-coupled” model provided O_3_ forecast guidance for the northeastern US states (Kang et al., 2005; Otte et al., 2005; Eder et al., 2006) and formed the early version of the National Air Quality Forecasting Capability (NAQFC) that was first implemented for operations in September 2004 (https://www.weather.gov/sti/stimodeling_airquality_predictions, last access: 5 April 2022). The NAQFC was further developed at NOAA and collaborating laboratories (Mathur et al., 2008; McKeen et al., 2005, 2007, 2009) and was comprehensively evaluated in Eder et al. (2009). The NAQFC has been continuously advanced to provide both O_3_ and PM_2.5_ forecast guidance for the entire conterminous US (CONUS), has expanded its predictions to both Alaska and Hawaii, and has provided pivotal air quality forecast guidance to a multitude of stakeholders to help protect human health and the environment (Stajner et al., 2011; Lee et al., 2017; Huang et al., 2017). Prior to the advanced version described in this paper, the NAQFC used the offline-coupled North American Mesoscale Model Forecast System on the B-Grid (NMMB) (Black, 1994; Janjic and Gall, 2012) and CMAQv5.0.2 (US EPA, 2014). The NAQFC provides forecast guidance for O_3_, PM_2.5_, wildfire smoke, and dust at a horizontal grid spacing of 12 km over a domain centered on the CONUS, Alaska, and Hawaii domains.

NOAA’s National Weather Service (NWS) transitioned operationally in June 2019 to use a new dynamical core known as the Finite-Volume Cubed-Sphere (FV3) in the Global Forecast System (GFS) model. Both the National Aeronautics and Space Administration (NASA) and NOAA’s Geophysical Fluid Dynamics Laboratory (GFDL; https://www.gfdl.noaa.gov/, last access: 5 April 2022) have developed and advanced FV3 over the past few decades (Lin et al., 1994; Lin and Rood, 1996; Lin, 2004; Putman and Lin, 2007; Chen et al., 2013; Harris and Lin, 2013; Harris et al., 2016; Zhou et al., 2019). Overall, the switch to a FV3-based dynamical core with advancements to GFS’s observation quality control, data assimilation, and model physical parameterizations (from the National Center for Environmental Prediction) significantly increases the accuracy of 1–2 d and longer (e.g., 3–7 d) weather forecasts (Chen et al., 2019). Other advantages of FV3GFS are improved computational efficiency and adaptable scaling, enhanced and flexible vertical resolution, and improved representation of atmospheric circulation and weather patterns across different horizontal scales (Yang et al., 2020; https://www.weather.gov/media/notification/pns20–44gfs_v16.pdf, last access: 5 April 2022; https://www.emc.ncep.noaa.gov/emc/pages/numerical_forecast_systems/gfs.php, last access: 5 April 2022; https://ufscommunity.org/wp-content/uploads/2020/10/UFS_Webnair_GFSv16_20201022_FanglinYang.pdf, last access: 5 April 2022).

The improved representation of atmospheric conditions, circulation, transport, and precipitation in GFS are pivotal to the accuracy of chemical predictions when coupled to RT-AQF models. Since 2017, there has also been significant efforts at NOAA to use version 15 of FV3GFS (hereafter, GFSv15) rather than NMMB as the meteorological driver for CMAQ in the NAQFC (Huang et al., 2017, 2018, 2019). Huang et al. (2020) and Chen et al. (2021) demonstrated that a version of the GFS-driven CMAQv5.0.2 (GFSv15-CMAQ) forecasting system had partly improved O_3_ predictions compared to the NMMB-driven CMAQ (NMMB-CMAQ) system but that the GFSv15-CMAQ had large biases for PM_2.5_ that still need improvement.

Concurrently, at NOAA there has been a major upgrade of GFS from version 15 to 16 (GFSv16), which includes a number of major developmental advances to the system (see Sect. 2 of this paper). Thus, there was an opportunity to simultaneously upgrade and streamline the meteorological coupling between the GFSv16 and a more updated, “state-of-the-science” version of CMAQ at the US EPA (US EPA, 2019; Appel et al., 2021). The current CMAQv5.0.2 used in the NMMB-CMAQ and experimental GFSv15-CMAQ is outdated scientifically with numerous deficiencies, many of which led to the elevated biases and error as described in Huang et al. (2017, 2020) and Chen et al. (2021). Hence, there is a need to update the NAQFC to actively developing versions of both FV3GFS and CMAQ.

The main objectives of this paper are to describe the development of the GFSv16 coupling with a state-of-the-science CMAQ model for the advanced updates to NAQFC that includes numerous other RT-AQF science advances (Sect. 2). We also describe the new simulation design and input observations, and evaluate the meteorological and air quality predictions across the US compared to the now discontinued NMMB-CMAQ system for NAQFC (Sects. 3 and 4). We conclude with a summary of NOAA-EPA Atmosphere Chemistry Coupler (NACC)-CMAQ serving as the current (since 20 July 2021) operational NAQFC, as well as longer-term goals (Sect. 5). We hypothesize that advancing to closer state-of-the-science meteorological and chemical transport models will improve atmospheric chemical composition predictions, and the resulting air quality forecasts will better protect human health across the US.

## 2 Methods

### 2.1 Updated meteorological and surface drivers

#### 2.1.1 The Global Forecast System version 16

**Figure 1.**
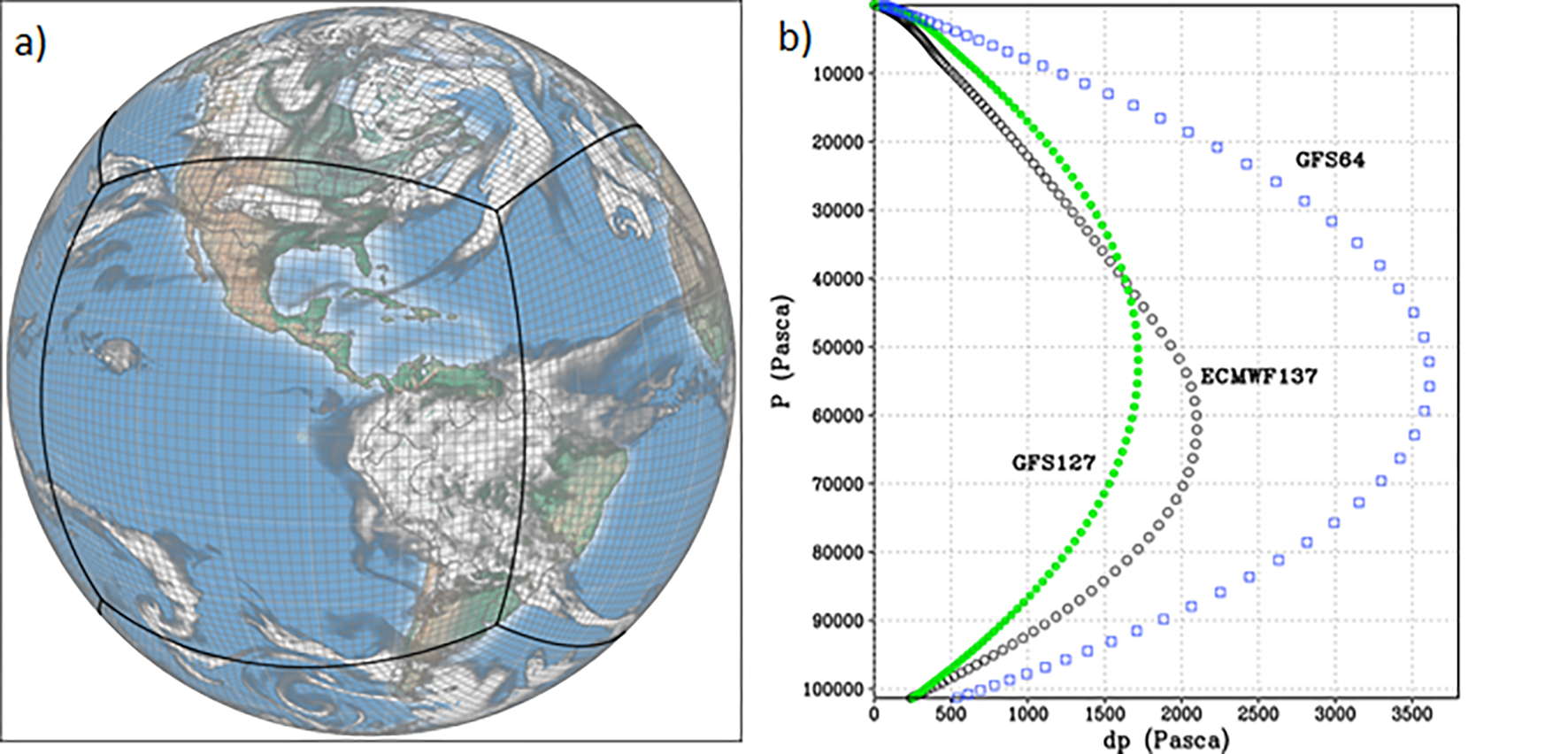
The (**a**) native FV3 gnomonic cubed-sphere grid at C48 (2°) resolution (image courtesy of Dusan Jovic, NOAA) and (**b**) vertical resolution (*P* vs. d*P*) for the upgraded GFSv16 (green) compared to the previous GFSv15.3 (blue) and the European Centre for Medium-Range Weather Forecasts (ECMWF) model (black).

The Environmental Modeling Center (EMC) at NOAA continuously develops and improves the GFS model, which has been in operation at the National Weather Service since 1980. EMC has recently upgraded the GFS model from v15.3 to v16 in February 2021, and the major upgrade improves the model forecast performance while also providing enhanced forecast products. Some of the major structural changes to GFSv16 (compared to previous GFS versions) include increased vertical layers (resolution) from 64 to 127 (Fig. 1) and an extended model top from 54 (upper stratosphere) to 80 km (mesopause). GFSv16 also has a thinner first model layer thickness (20 m) and higher-resolution global horizontal grids of ~ 25 and 13 km (Yang et al., 2020; https://www.weather.gov/media/notification/pns20–44gfs_v16.pdf, last access: 5 April 2022; https://www.emc.ncep.noaa.gov/emc/pages/numerical_forecast_systems/gfs.php, last access: 5 April 2022; https://ufscommunity.org/wp-content/uploads/2020/10/UFS_Webnair_GFSv16_20201022_FanglinYang.pdf, last access: 5 April 2022).

The GFSv16 has significantly improved its physical parameterizations (e.g., planetary boundary layer (PBL), gravity wave, radiation, clouds and precipitation, land surface, and surface layer schemes) and upgraded to the Global Data Assimilation System (GDAS) version 16 (Yang et al., 2020; https://www.weather.gov/media/notification/pns20–44gfs_v16.pdf, last access: 5 April 2022; https://www.emc.ncep.noaa.gov/emc/pages/numerical_forecast_systems/gfs.php, last access: 5 April 2022; https://ufscommunity.org/wp-content/uploads/2020/10/UFS_Webnair_GFSv16_20201022_FanglinYang.pdf, last access: 5 April 2022).

The global GFSv16 has changed the format of forecast output history files from binary (nemsio) to netCDF with zlib compression (data volume reduced by about 60 %), and provides the hourly (important for CMAQ predictions) output for a 72 h (3 d) forecast each day. The prior operational NAQFC (NMMB-CMAQ) forecast is only out to 48 h (2 d). The netCDF output is available (via live disk and archives) to all of NOAA’s downstream model applications and is in the form of a rectangular Gaussian grid with a globally uniform grid resolution of ~ 13 km (referred to as “C768”) and a set number of latitude and longitude coordinates. The NOAA GFDL website provides more information about FV3 and its grids (https://www.gfdl.noaa.gov/fv3/, last access: 5 April 2022). There are additional new surface fields in the GFSv16 output, which include plant canopy surface water, surface temperature and moisture at four below-ground levels (0–0.1, 0.1–0.4, 0.4–1, 1–2 m), surface roughness, soil and vegetation type, and friction velocity.

#### 2.1.2 The NOAA-EPA Atmosphere Chemistry Coupler (NACC)

**Figure 2.**
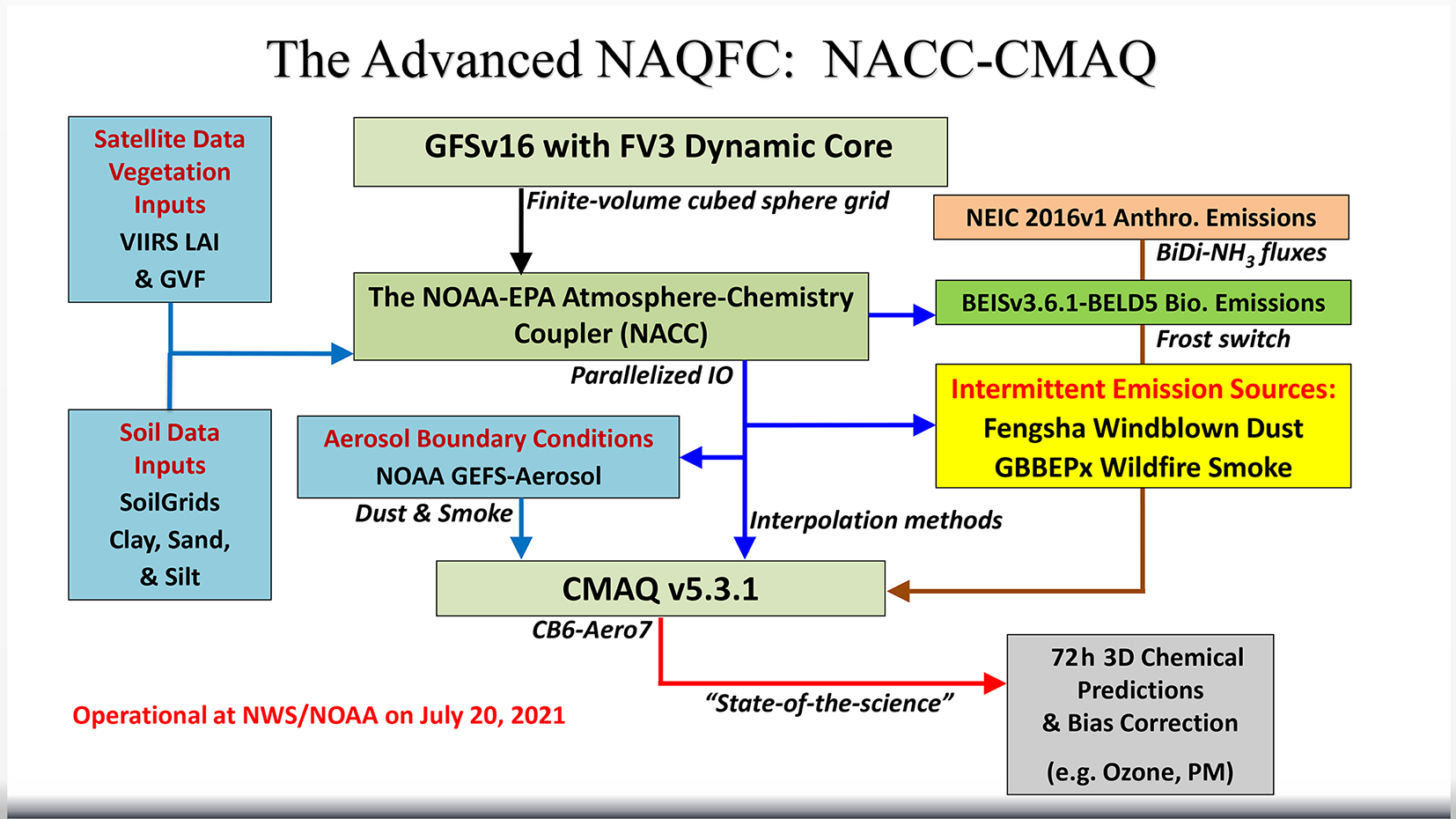
Schematic of the advanced NAQFC based on NACC-CMAQ.

The meteorological–chemical coupling of the GFSv16 to the regional, state-of-the-science CMAQ v5.3.1 model (US EPA, 2019; Appel et al., 2021) is achieved via the NOAA-EPA Atmosphere Chemistry Coupler (NACC) version 1 (NACC, i.e., “knack”, meaning an acquired skill), which is adapted from the US EPA’s Meteorology-Chemistry Interface Processor (MCIP) version 5 (Otte and Pleim, 2010; https://github.com/USEPA/CMAQ, last access: 5 April 2022). The NACC and CMAQ coupling (hereafter referred to as NACC-CMAQ) involves a number of structural and scientific advancements (Fig. 2, i.e., the advanced NAQFC) compared to the previous operational NMMB-CMAQ; hereafter referred to as “prior NAQFC”.

**Figure 3.**
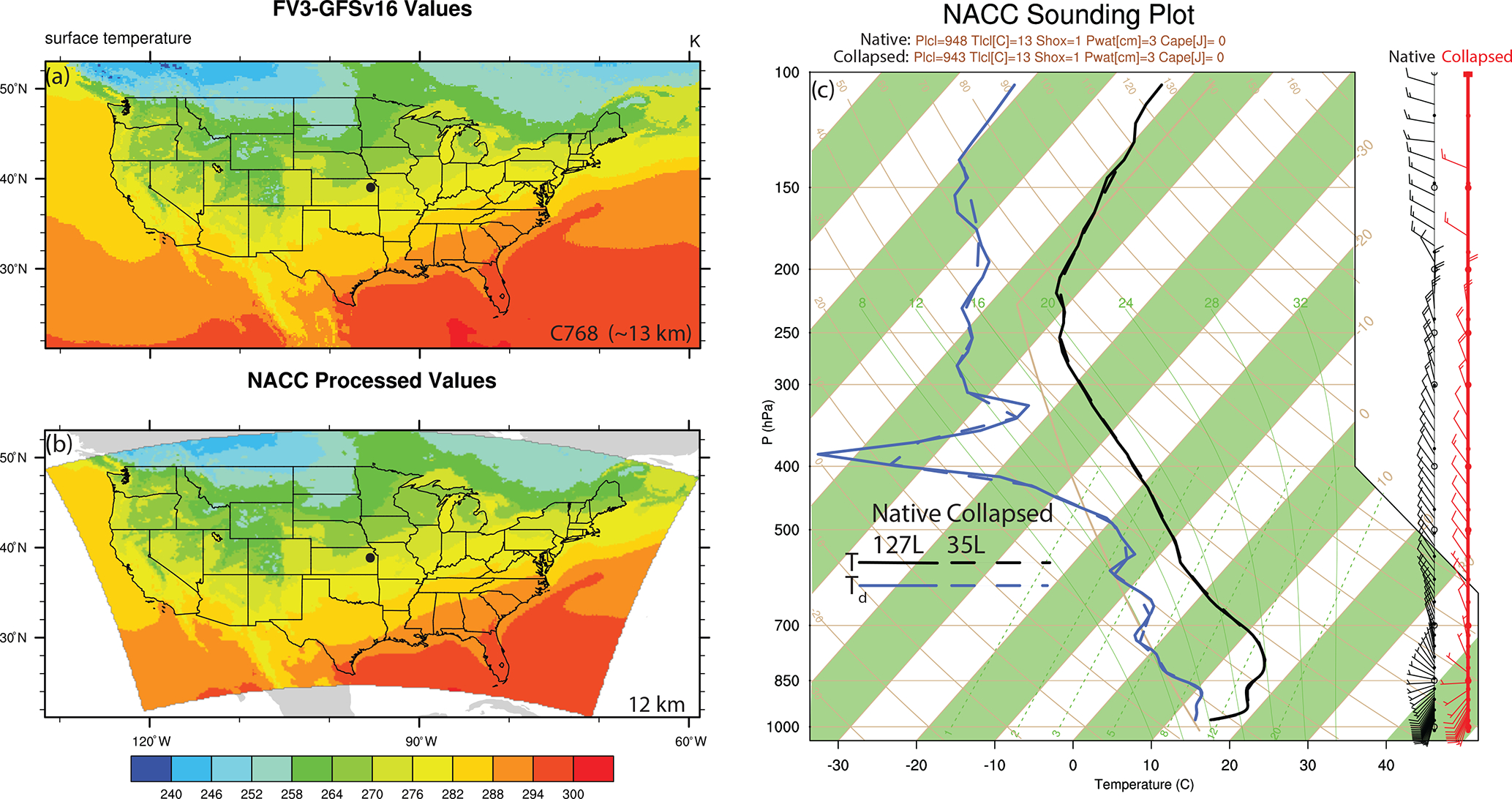
Examples of the NACC-CMAQ (**a**) GFSv16 Gaussian grid surface temperature (C768 ~ 13 km), (**b**) associated bilinear horizontal interpolation NACC LCC output (12 km), and (**c**) Skew-T Log-P diagram of both native GFSv16 (127 layers; solid) and interpolated NACC (35 layers; dashed) profiles of temperature (black), dew point (blue), and wind speed and direction (wind barbs, with native shown in black and collapsed shown in red). The example sounding pertains to a date of 24 September 2020 at the closest model grid square to 39.07° N and 95.62° W (black dot in **a–b**).

The major structural changes to NACC-CMAQ include a variable-dependent bilinear or nearest-neighbor horizontal interpolation of the GFSv16 Gaussian gridded (~ 13 km) fields (e.g., 2 m temperature, 2 m specific humidity, 10 m wind speed and direction, and sea level pressure) to a Lambert conic conformal (LCC) projection at 12 km horizontal grid spacing (same as the prior NAQFC) (Fig. 3a–b). NACC-CMAQ also includes a redefined vertical structure based on vertical interpolation (i.e., collapsing) to a 35-layer configuration (Fig. 3c) that is identical to the prior NAQFC.

Time-splitting techniques based on message passing interface (MPI) commands parallelize the GFSv16-to-NACC input and output (IO), which vastly improves the computational efficiency for the updated 72 h forecast period. The NACC-CMAQ coupling is more unified and streamlined compared to prior NAQFC (Stajner et al., 2011; Lee et al., 2017; Huang et al., 2017) and experimental GFSv15-CMAQ (Huang et al., 2018, 2019) applications, while eliminating multiple pre- and post-processing steps. The NACC-CMAQ processing steps are therefore subject to less uncertainty that comes with multiple grid interpolations and restructuring used previously and are more computationally efficient for the 72 h forecast window. Furthermore, the vertical interpolation from 127 to 35 layers results in an excellent agreement in the vertical structure of key atmospheric state variables (Fig. 3c). While this example is only for the central US, other model grid cell locations in the eastern and western US also demonstrate excellent agreement in the native and collapsed vertical structure in NACC (not shown). While NACC-CMAQ domains for Alaska and Hawaii are also available for NAQFC, this paper focuses only on the results inside the CONUS domain.

The left side of Fig. 2 shows that NACC-CMAQ incorporates high-resolution satellite data for a 2018–2020 climatological (12-month) averaged leaf area index (LAI), which is based on the Visible Infrared Imager Radiometer Suite (VIIRS) 8 d, level 4 global 500 m sinusoidal (SIN) grid, V001 product (Myneni and Knyazikhin, 2018; https://lpdaac.usgs.gov/products/vnp15a2hv001/, last access: 5 April 2022). This is a substantial update from the prior NAQFC, which assumed an unrealistic static LAI value of 4 across the entire domain. The NOAA product for near-real-time (NRT) greenness vegetation fraction (GVF) from VIIRS (Ding and Zhu, 2018; https://www.ospo.noaa.gov/Products/land/gvf/, last access: 5 April 2022) is used as a dynamic, direct input in NACC-CMAQ instead of using the GFSv16 vegetation fraction (VEG). Both VIIRS LAI and GVF are preprocessed, and NACC performs nearest-neighbor interpolation to the NAQFC grid.

More realistic land cover characteristics have shown to improve modeled meteorology, chemistry, and surface–atmosphere exchange processes in the coupled Weather Research and Forecasting (WRF; Powers et al., 2017; Skamarock and Klemp, 2008) and CMAQ model (e.g., Ran et al., 2016; Campbell et al., 2019). Test results here show that rapid-refresh of high-resolution VIIRS LAI and GVF in NACC have distinct differences compared to an older 2010 MODIS International Geosphere–Biosphere Programme (IGBP) LAI climatology and GFSv16-based VEG, respectively (Figs. S1–S2 in the Supplement). The updated dynamic LAI and GVF alter biogenic emissions, dry deposition, and resulting concentrations of gases and aerosols in NACC-CMAQ, particularly during the fall transition month of October 2020 (Fig. S3).

NACC-CMAQ also uses global gridded soil information based on the 2019 SoilGrids™ 250 m resolution data (https://www.isric.org/explore/soilgrids, last access: 5 April 2022) to drive an inline FENGSHA windblown dust model (Fu et al., 2014; Huang et al., 2015; Dong et al., 2016) in NACC-CMAQ (Fig. 2). Section 2.2 below provides more information on the specific parameters used in FENGSHA.

As in the prior NAQFC, the chemical initial conditions (beginning on 20 July 2021 for NACC-CMAQ) are taken from the previous day’s (CMAQ) forecast output, and a NRT bias-correction using AirNow surface observations (https://www.airnow.gov/, last access: 5 April 2022) is applied to the 72 h predictions of O_3_ and PM_2.5_ (Fig. 2). Huang et al. (2017) provides more information on the bias-correction technique.

### 2.2 Updated chemistry, emissions, and air–surface exchange processes

#### 2.2.1 The Community Multiscale Air Quality (CMAQ) model version 5.3.1

A major update in NACC-CMAQ is coupling the GFSv16 to a state-of-the-science chemical transport model, CMAQv5.3.1 (US EPA, 2019; Appel et al., 2021) (Fig. 2). The prior NAQFC and experimental GFSv15-CMAQ both use CMAQv5.0.2, released in April 2014 (US EPA, 2014). The major release of CMAQv5.3 incorporates significant improvements to gas chemistry (e.g., halogen-mediated ozone loss), aerosol modules (e.g., improved secondary organic aerosol formation), photolysis rates, aqueous and heterogeneous chemistry, transport processes, air–surface exchange, emissions, and other structural and computational improvements (Appel et al., 2021). The use of CMAQv5.3.1 in NACC-CMAQ also contains a number of bug fixes to v5.3. Version 6 of the Carbon Bond (CB6) mechanism is used for gas-phase chemistry (Yarwood et al., 2010), and the updated US EPA’s AERO7 module is used for aerosol formation in NACC-CMAQ. The US EPA’s GitHub web page (https://github.com/USEPA/CMAQ/blob/master/DOCS/Release_Notes/README.md, last access: 5 April 2022) contains the CMAQv5.3 and v5.3.1 release notes, mechanism descriptions, and enhancements.

#### 2.2.2 National Emissions Inventory Collaborative (NEIC) 2016 version 1 emissions

The anthropogenic emissions modeling data may be the most influential input for chemical transport model predictions in any AQF system (Matthias et al., 2018). The model emissions are updated from National Emissions Inventory (NEI) 2014 version 2 (2014v2) that is used by the prior NAQFC to NEI Collaborative (NEIC) 2016 version 1 (2016v1) Emissions Modeling Platform (NEIC, 2019), which is based on updated models and datasets applied to the US Environmental Protection Agency’s (EPA) NEI2014v2. The prior NAQFC uses an older NEI2014v2 emissions dataset. There have been substantial updates to the NEIC2016v1, which include emission decreases for CO, NO_*x*_, SO_2_, and PM_2.5_ and increases in total VOC and ammonia (NH_3_) emissions compared to NEI2014v2 (NEIC, 2019). The intermittent, “event-based” emissions from wildfires and windblown dust, as well as persistent biogenic emissions sources, are not from the NEIC2016v1 but are instead dynamically predicted inline within NACC-CMAQ (described in following sections). The NEIC2016v1 area source (i.e., 2-D) emissions are given in a gridded netCDF/IOAPI format that are interpolated to the 12 km NAQFC domain. The NEIC2016v1 also provides major point source (i.e., 3-D) emissions from six sectors: commercial marine vehicles (CMV12 and CMV3), electricity-generating units (EGUs), non-EGUs, oil–gas sources, and “other” point sources. The anthropogenic point source plume rise is calculated inline within NACC-CMAQ using the Briggs plume rise method (Briggs, 1965). Slight adjustments are made to reduce the anthropogenic aerosol and fugitive dust emissions over snow and wet soil surfaces to account for different forecasted meteorology in GFSv16 compared to the conditions used in generating the NEIC2016v1.

We note that the NEIC2016v1 emissions are not projected into the actual forecast year, with the time lag being a long-recognized issue in NAQFC (e.g., Tong et al., 2012). Thus, the NACC-CMAQ air quality simulations for the fall of 2020 and the winter of 2021 are impacted by the COVID-19 pandemic, which resulted in spatiotemporal changes to emission patterns and ozone formation over the US in 2020 and beyond (Campbell et al., 2021). In addition, mobile source emissions have continued to decline since 2016, and thus it is likely that the emissions used in the analysis do not entirely reflect recent changes to the emissions compared to 2016 (almost 5 years earlier). We are actively working to improve the representativeness of anthropogenic emissions sources in NACC-CMAQ and next-generation versions of the NAQFC.

#### 2.2.3 Inline biogenic emissions and bidirectional NH_3_ fluxes

NACC-CMAQ uses the latest version of the Biogenic Emission Inventory System (BEIS) v3.6.1 (Vukovich and Pierce, 2002; Schwede, 2005) for estimating the biogenic VOC (BVOC) emissions. BEISv3.6.1 includes updated vegetation inputs and advanced two-layer canopy model formulations for estimating leaf (sun and shade) temperatures and vegetation data (Weiss and Norman, 1985; Campbell and Norman, 1998; Niinemets et al., 2010; Bash et al., 2016). NACC-CMAQ also uses the revised Biogenic Emissions Landuse Dataset version 5 (BELD5), which includes a newer version of the Forest Inventory and Analysis (FIA) version 8.0 and updated agricultural land use from the 2017 US Department of Agriculture (USDA) crop data layer. The BELD5 dataset also uses a MODIS 21-category land use dataset with lakes identified separately from oceans. The prior NAQFC used a much older BELD3 version (https://www.epa.gov/air-emissions-modeling/biogenic-emissions-landuse-database-version-3-beld3, last access: 6 April 2022).

The prior NAQFC also only considered summer factors in BEIS and did not capture seasonal (summer and winter) changes to the normalized biogenic emissions factors (specific to vegetation species). NACC-CMAQ is improved and uses a new “vegetation frost switch” that adjusts between summer and winter normalized emission factors in BEISv3.6.1 based on the calendar date and 2 m temperature (TEMP2). In NACC, a new time-dependent variable, “SEASON” is equal to 1 during the growing season or equal to 0 outside the growing season. The SEASON is (boreal) summer if the calendar date is on or between 15 April and 15 October but switches to winter if TEMP2 drops below 28 °F (−2 °C), and it is winter if the date is on or between 16 October and 14 April but switches to summer if TEMP2 rises above 32 °F (0 °C). Thus, the SEASON variable in NACC-CMAQ differs from typical retrospective CMAQ applications and is more dynamic with hourly variability based on the GFSv16-forecasted TEMP2. Test results show generally improved model performance for all US regions in December 2020 (winter) with vegetation frost switch compared to using only summer season normalized emissions (Table S1 in the Supplement). Using BELD5 further improves model performance and reduces the error in all CONUS regions compared to the older BELD3 used in December 2020 tests (Table S1).

NACC-CMAQ includes bidirectional NH_3_ (BIDI-NH_3_) for NH_3_ fluxes (i.e., both deposition and evasion) in the CMAQv5.3.1 “M3Dry” deposition model (Nemitz et al., 2000; Cooter et al., 2010; Massad et al., 2010; Pleim and Ran, 2011; Bash et al., 2010, 2013; Pleim et al., 2013; 2019). Here, the NH_3_ fertilizer emissions are removed from the base NEIC2016v1 inventory to avoid double counting, as the inline BIDI-NH_3_ module calculates these fluxes. The BIDI-NH_3_ module typically requires daily inputs (e.g., soil ammonia content, soil pH, soil moisture, and other soil characteristics) from the USDA’s Environmental Policy Integrated Climate (EPIC) agroecosystem model (https://epicapex.tamu.edu/epic/, last access: 5 April 2022; Williams et al., 1995) to calculate the soil ammonia concentrations that are combined with air concentrations in CMAQ to calculate BIDI-NH_3_ fluxes. Typically, the Fertilizer Emission Scenario Tool (FEST-C, https://www.cmascenter.org/fest-c/, last access: 5 April 2022) processes the necessary meteorological conditions for integration with the EPIC simulation for input to CMAQ (Ran et al., 2011; Cooter et al., 2012). Use of the EPIC/FEST-C system is not feasible in an NRT operational forecasting model, and thus we use a pregenerated, full-year 2011 EPIC/FEST-C simulation based on Campbell et al. (2019) for the daily inputs to BIDI-NH_3_ in NACC-CMAQ. NACC-CMAQ directly uses the GFSv16 soil moisture conditions in place of the FEST-C processed soil conditions required for the latest version of BIDI-NH_3_ in CMAQv5.3.1 (Pleim et al., 2019).

#### 2.2.4 Inline wildfire smoke and windblown dust emissions

Wildfires have been increasing in size (Westerling et al., 2006) and potentially in severity (Miller et al., 2009) over the past decades. Wildfire smoke outbreaks can lead to extreme concentrations of PM_2.5_ and enhanced O_3_ and are major concerns for air quality forecasting and consequential human and ecosystem health impacts. NACC-CMAQ includes a new inline calculation of wildfire smoke emissions based on the Blended Global Biomass Burning Emissions Product (GBBEPx V3; Zhang et al., 2012, 2014). GBBEPx provides daily global biomass burning emissions (PM_2.5_; black carbon, BC; organic carbon, OC; NO_*x*_; NH_3_; CO; and SO_2_). It blends fire observations from two sensors, including the Moderate Resolution Imaging Spectroradiometer (MODIS) on the NASA Terra and Aqua satellites and the Visible Infrared Imaging Spectrometer (VIIRS) on the Suomi National Polar-orbiting Partnership (SNPP) and Joint Polar-orbiting Satellite System 1 (JPSS1) satellites. The GBBEPx data are further processed to prepare model-ready emission datasets. First, the 0.1 × 0.1° latitude and longitude data are converted into the NAQFC LCC projection. US EPA-based Sparse Matrix Operator Kernel Emissions (SMOKE) fire speciation and diurnal profiles provide the PM speciation and diurnal patterns in NACC-CMAQ, respectively, while both land use and region are used to identify fire types. The fire duration persists for the 72 h forecast period (with scaling of 1.0, 0.25, and 0.25 for day 1, 2, and 3, respectively) for wildfires identified when the grid cell forest fraction is > 0.4. In the eastern US (longitude east of 100° W), however, the fires are assumed to be mainly prescribed burns in forested regions that only persist for the first 24 h. The wildfire plume rise is calculated inline within NACC-CMAQ using either the Briggs (1965) or Sofiev et al. (2012) algorithms (Wilkins et al., 2019); currently the Briggs method is used by default.

Climate models project warming and drying trends in the southwestern US, where intermittent windblown dust storms are becoming more frequent with the occurrence of drought (Tong et al., 2017) or even “megadrought” conditions (Williams et al., 2020). Windblown dust storms can lead to extreme levels of coarse-mode particulate matter (i.e., PM_10_) and cause detrimental effects to human and agroecosystem health and visibility. NACC-CMAQ includes a novel inline methodology for calculating windblown dust based on the FENGSHA model (Huang et al., 2015; Dong et al., 2016). In NACC-CMAQ, the potential for vertical dust flux in FENGSHA is generally controlled by the sediment supply map (SSM), and the magnitude of the friction velocity (USTAR) compared to a threshold friction velocity (UTHR) that determines the USTAR needed to transfer dust from soil surfaces to the atmosphere. The UTHR is dependent on the land cover, soil type, and soil moisture. The SoilGrids™ 250 m high-resolution dataset (https://www.isric.org/explore/soilgrids, last access: 5 April 2022) provides the necessary clay, silt, and sand fractions used to calculate the SSM.

### 2.3 Updated dynamic aerosol boundary conditions

The chemical lateral boundary conditions (CLBCs) are critical to the prediction accuracy of regional chemical transport models, particularly during intrusion events (Tang et al., 2009, 2021). The CLBCs represent the spatiotemporal distribution of chemical species along the lateral boundaries of the domain of a regional model. NACC-CMAQ uses methods described in Tang et al. (2021) and implements dynamic CLBCs (updated every 6 h) for dust and smoke aerosol data that are extracted (and mapped to CMAQ CB6-Aero7 species) from the NOAA operational global atmospheric aerosol model, known as the Global Ensemble Forecast-Aerosols (GEFS-Aerosols) member (Fig. 2). GEFS-Aerosols is also based on the FV3GFS dynamical core, which uses the Goddard Chemistry Aerosol Radiation and Transport (GOCART) model for its sulfate, dust, BC, OC, and sea salt aerosol predictions (Chin et al., 2000, 2002; Ginoux et al., 2001). GEFS-Aerosols uses the same wildfire smoke and windblown dust dataset and algorithms as in NACC-CMAQ. The operational version of GEFS-Aerosols is run by the NWS as a special unperturbed forecast of the Global Ensemble Forecast System version 12 (https://www.ncdc.noaa.gov/data-access/model-data/model-datasets/global-ensemble-forecast-system-gefs, last access: 5 April 2022), which provides an ensemble forecast product four times per day. Dynamic CLBCs capture the signals of aerosol intrusion events such as biomass burning or windblown dust plumes from outside the domain, which can improve the prediction accuracy of downstream O_3_ and PM_2.5_ concentrations at the surface (Tang et al., 2021).

## 3 Simulation design and evaluation protocol

Table 1 summarizes the GFSv16 and NACC-CMAQv5.3.1 model configuration described in Sect. 2, as well as some additional model details. The model components and configurations used in prior NAQFC system are summarized in Table S2 (based on Lee et al., 2017) for comparison.

The simulation design consists of evaluations of continuous 1-month NACC-CMAQ (72 h, 3 d forecast) and prior NAQFC (48 h, 2 d forecast) simulations for September 2020 (late summer–fall period) and January 2021 (winter period) (with a previous 1-month spin-up and training data period) over the CONUS at a horizontal grid spacing of 12 km (Table 1). September 2020 is used for the warm season because it is the closest month to summer when both the NACC-CMAQ and prior operational NAQFC systems were simultaneously run. The prior operational NAQFC was discontinued on 20 July 2021 due to computational constraints at NWS/NOAA.

The Surface Weather Observations and Reports for Aviation Routine Weather Reports (METAR), collected by NCEP’s Meteorological Assimilation Data Ingest System (MADIS) (https://madis.ncep.noaa.gov/madis_metar.shtml, last access: 5 April 2022), provide observations of TEMP2, 2 m specific humidity (Q2), and 10 m wind speed (WSPD10). The World Radiation Monitoring Center’s (WRMC’s) Baseline Solar Radiation Network (BSRN) (https://bsrn.awi.de/, last access: 5 April 2022; Driemel et al., 2018) and US Surface Radiation Network (SURFRAD; https://gml.noaa.gov/grad/surfrad/, last access: 5 April 2022) provide shortwave radiation observations at the ground (SWDOWN). The PRISM Climate Group, Northwest Alliance for Computational Science and Engineering, at Oregon State University (https://prism.oregonstate.edu/l, last access: 5 May 2021) provide gridded total precipitation observations (PRECIP). The National Oceanic and Atmospheric Administration (NOAA) Earth System Research Laboratory’s (ESRL’s) Radiosonde Database (RAOB) (https://ruc.noaa.gov/raobs/, last access: 5 April 2022) provides vertical profile observations of temperature, relative humidity, and wind speed. The US EPA Air Quality System (AQS; https://www.epa.gov/aqs, last access: 5 April 2022) and near-real-time AirNow observational networks (https://www.airnow.gov/, last access: 5 April 2022) provide near-surface O_3_ and PM_2.5_ measurements.

The statistical measures used to evaluate the meteorological–chemical coupling and air quality predictions include the mean bias (MB), normalized mean bias (NMB), normalized mean error (NME), root-mean-square error (RMSE), anomaly correlation coefficient (ACC), Pearson’s correlation coefficient (*R*), and index of agreement (IOA). Statistical measures such as *R*, NMB, and NME provide measures of the associativity (i.e., correlation), bias, and accuracy, respectively, of specific modeled surface and vertical meteorology and surface O_3_ and PM_2.5_. The meteorological and chemical evaluations use the publicly available US EPA Atmospheric Model Evaluation Tool (AMET; Appel et al., 2011) and NOAA/ARL Model and Observation Evaluation Toolkit (MONET; Baker et al., 2017).

## 4 Results

### 4.1 Meteorological analysis

**Figure 4.**
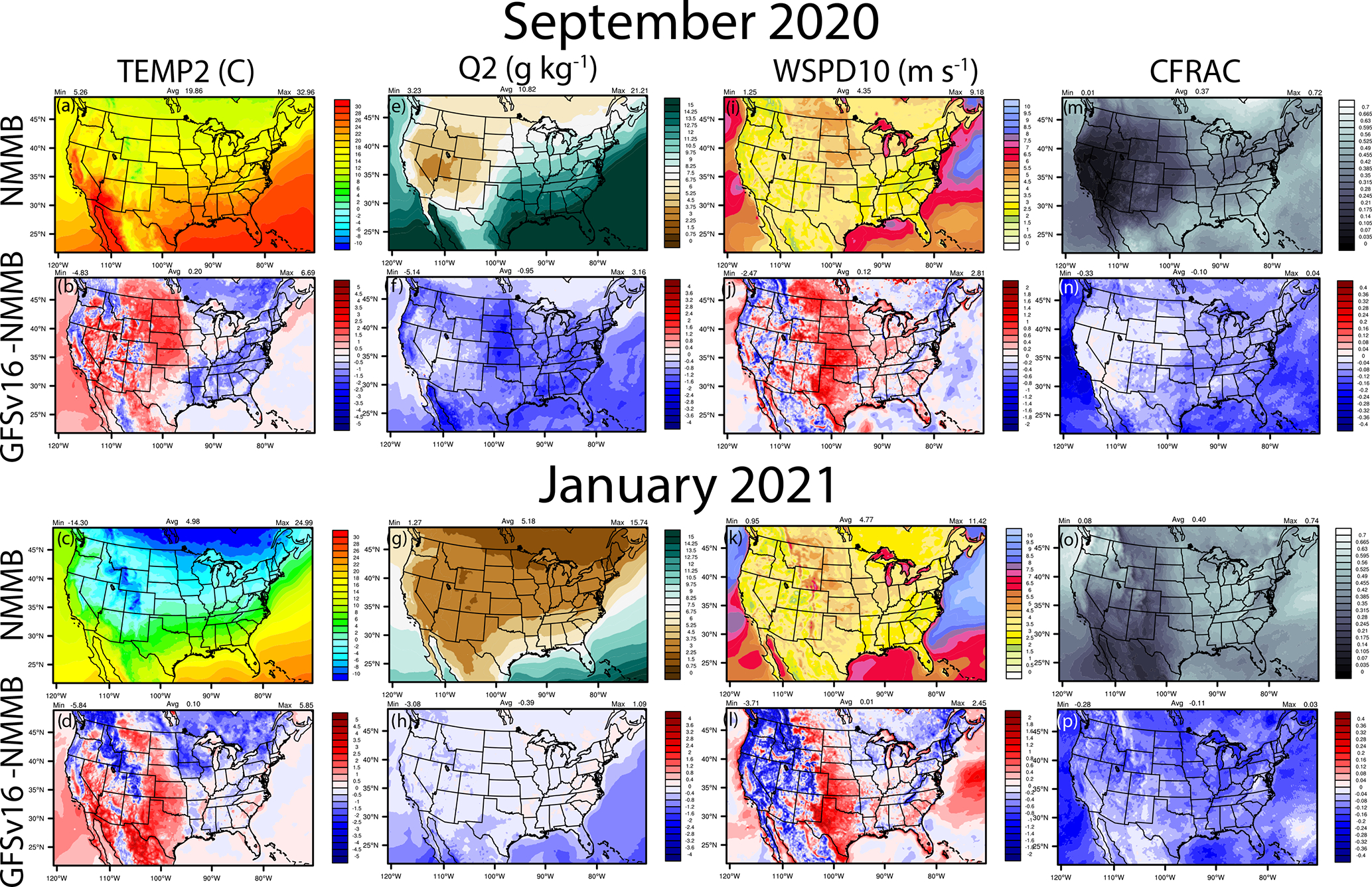
September 2020 and January 2021 spatial average plots for NMMB (prior NAQFC) and the absolute differences for GFSv16 (NACC) – NMMB for TEMP2, Q2, WSPD10 and CFRAC.

**Figure 5.**
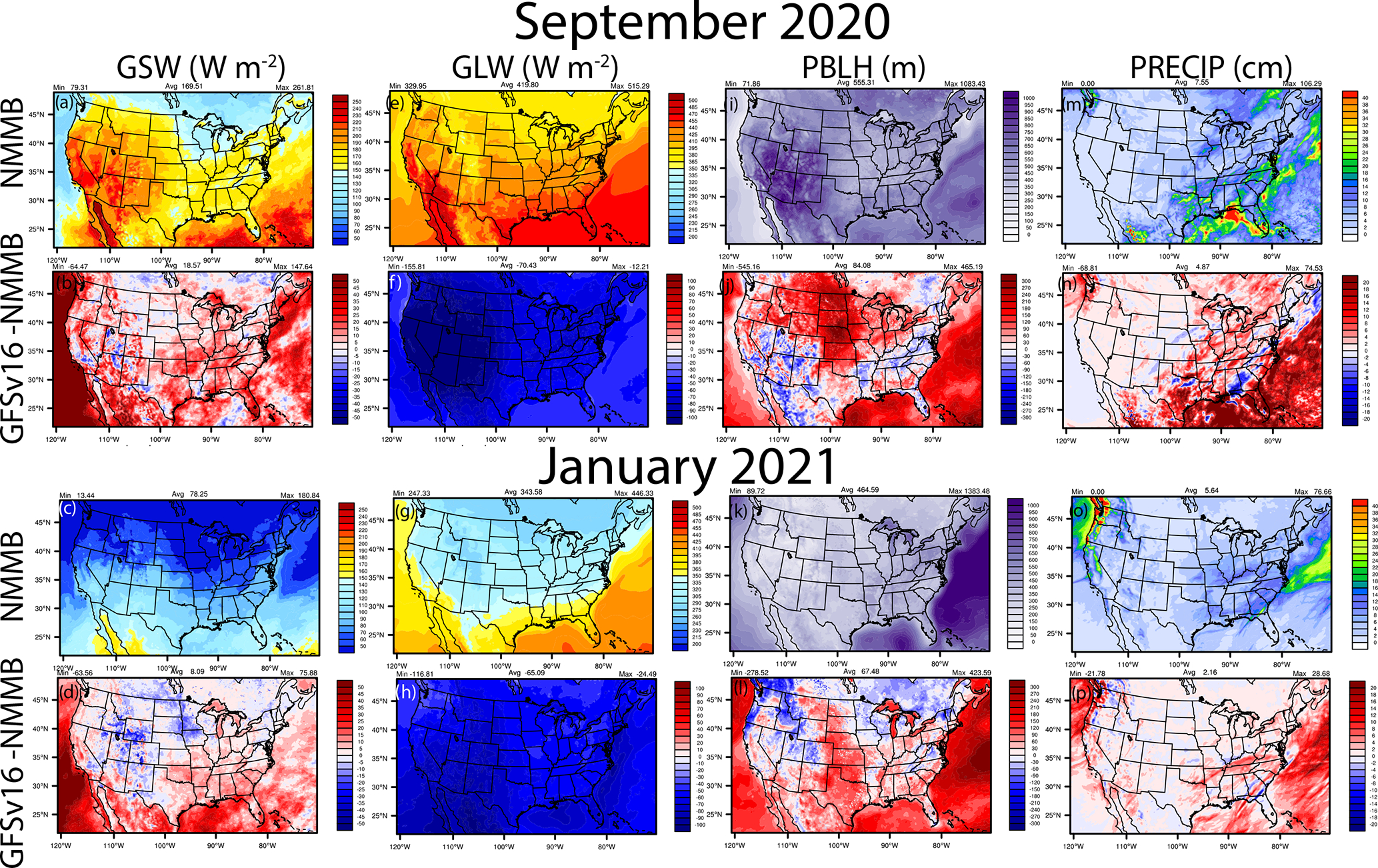
The same as Fig. 4 but for GSW, GLW, PBLH, and PRECIP.

Compared to NMMB used in the prior NAQFC, the GFSv16 model has lower actual TEMP2 in the east and southeast and parts of the northwest (Fig. 4a–d) but has higher TEMP2 in the central Great Plains, northern Great Plains, and parts of the western and southwestern US, with higher 10 m wind speeds (WSPD10) in these regions (Fig. 4i–l). GFSv16 is drier with widespread lower 2 m specific humidity (Q2; Fig. 4e–h) and lower cloud fractions (CFRAC) (Fig. 4m–p), higher solar radiation absorbed at the ground (GSW; Fig. 5a–d), lower longwave radiation absorbed at the ground (GLW; Fig. 5e–h), deeper planetary boundary layer height (PBLH; Fig. 5i–l), and generally more regions of increased precipitation (PRECIP; Fig. 5m–p). Differences in the CFRAC are (in part) impacted by differences in the model definition of cloud cover; NMMB uses a binary cloud cover definition at each grid point, while GFSv16 uses fractional cloud cover to calculate CFRAC. For stable conditions, the PBLH in the prior NAQFC is re-diagnosed based on the Troen and Mahrt (1986) incremental calculation of the bulk Richardson number (*Ri*_b_) from the surface up to a height above the neutral buoyancy level (i.e., approaching the critical Richardson number, *Ri*_crit_) in the Asymmetric Convective Model v2 (ACM2) PBL scheme in CMAQ (Pleim 2007a, b). For unstable conditions, the re-diagnosed ACM2 uses a slightly different PBLH formulation based on first finding the convectively unstable mixing layer (*z*_mix_) and then defining the point where *Ri*_b_ = *Ri*_crit_ for the entrainment layer above *z*_mix_. For both stable and unstable conditions, however, NACC-CMAQ directly uses the diagnosed PBLH from the turbulent kinetic energy (TKE)-based PBL scheme in GFSv16 (Table 1; Han and Bretherton, 2019), which is also based on the Troen and Mahrt (1986) incremental *Ri*_b_ formulation. Thus, NACC/GFSv16-CMAQ calculation is similar to the re-diagnosed ACM2 PBLH for nighttime-stable conditions (with slight differences in *Ri*_crit_ values), while there exists some distinct differences in their daytime-unstable PBLH formulations and *Ri*_crit_ calculations.

Consequently, the GFSv16 (NACC) and re-diagnosed ACM2 (prior NAQFC) diurnal PBLH patterns are similar at night; however, the GFSv16 PBLH is considerably higher than the prior NAQFC during the daytime for all regions in September and January (Figs. S4–S5).

The meteorological differences between GFSv16 and NMMB (Figs. 4–5) influence chemical predictions in CMAQ, which include a deeper daytime PBL and more precipitation that can effectively dilute the gaseous and aerosol concentrations for NACC-CMAQ in some regions across the CONUS. Areas of lower CFRAC and higher TEMP2 in GFSv16, however, will increase photolysis and daytime O_3_ formation in NACC-CMAQ in certain regions including the southern US and upper Great Plains. We note that although there are differences in the PBLH calculation methodologies between the prior NAQFC and NACC-CMAQ (particularly for the unstable daytime PBLH), the differences in near-surface meteorology (i.e., generally warmer and drier) conditions in the GFSv16 (Tables 2 and S2) also in part affect the differences in PBLH (Fig. 5i–l). These differences affect the pollutant mixing and dilution, and in part the resulting air quality predictions between the prior NAQFC and NACC-CMAQ (see Sect. 4.4 below).

### 4.2 Meteorological evaluation and metrics

**Figure 6.**
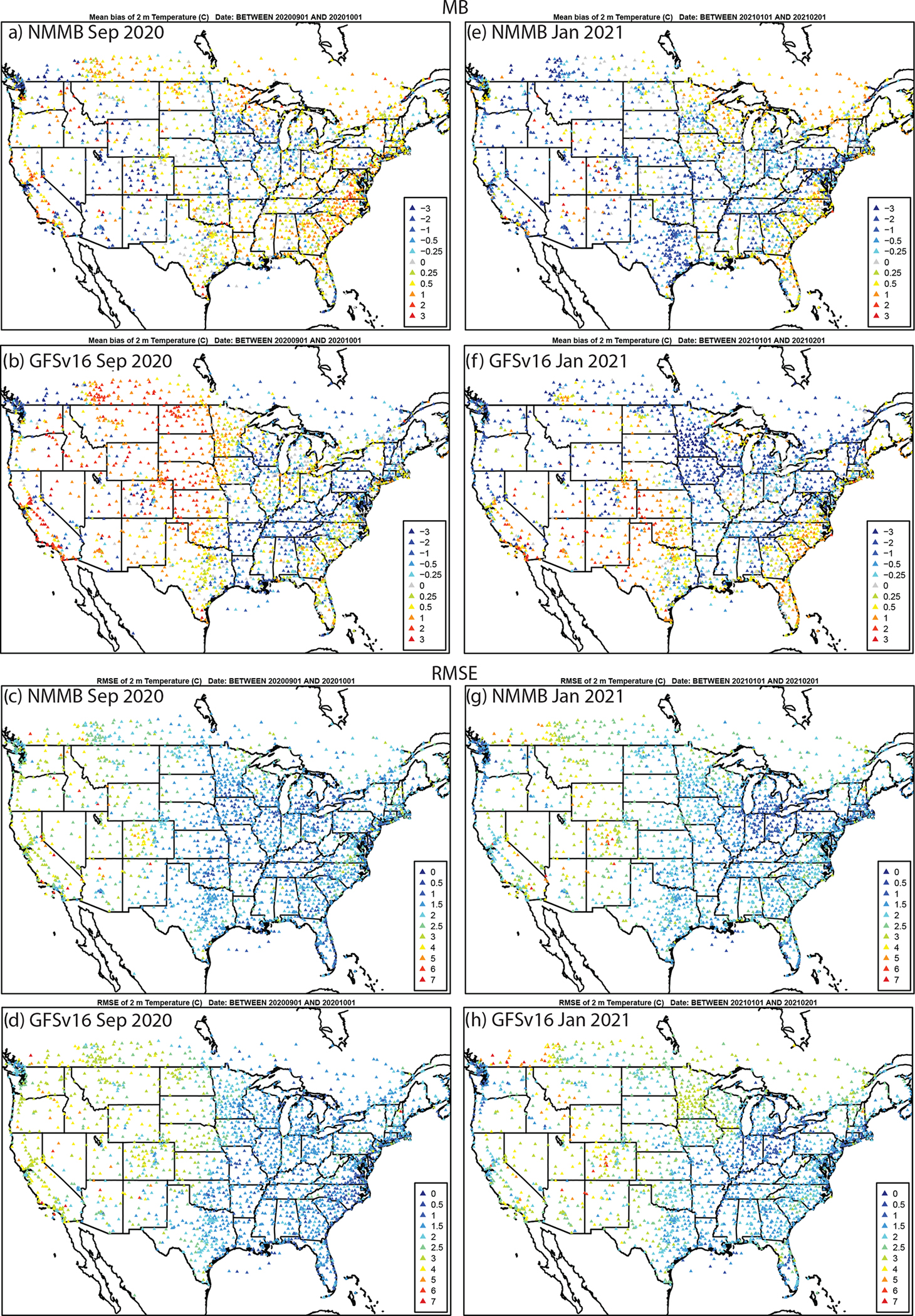
Average day 1 (0–24 h) forecasted TEMP2 MB (°C) and RMSE (°C) for NMMB and GFSv16 during (**a**)–(**d**) September 2020 and (**e**)–(**h**) January 2021 compared to METAR observations.

**Figure 7.**
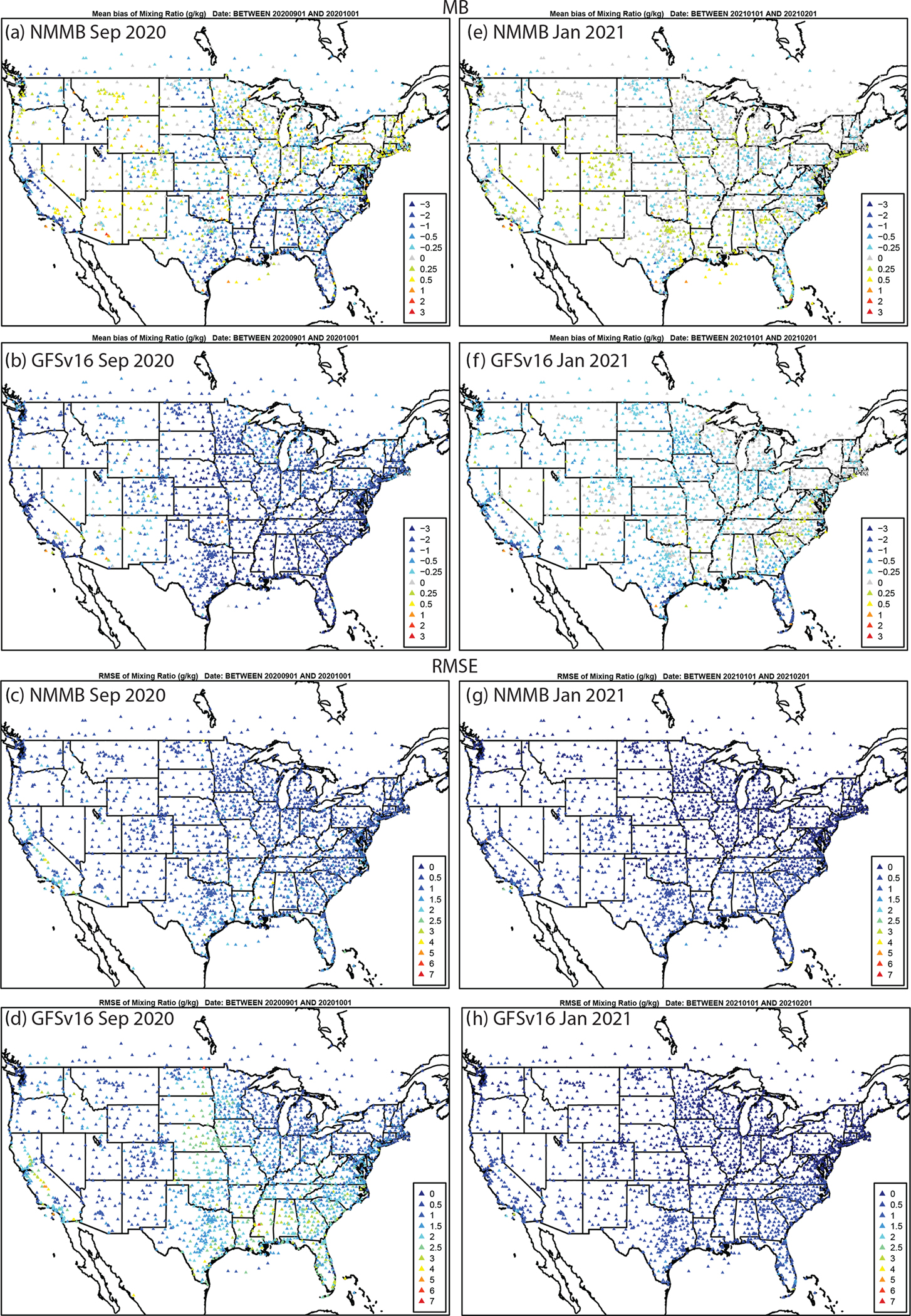
The same as Fig. 6 but for Q2 (g kg^−1^).

Evaluation of the simulated day 1 (0–24 h) forecasted meteorology against the METAR network shows that GFSv16 generally has a higher positive TEMP2 (warmer) bias (Fig. 6) in the west and a CONUS-wide higher negative Q2 (dry) bias (Fig. 7) compared to prior NMMB (i.e., prior NAQFC) in both September and January.

**Figure 8.**
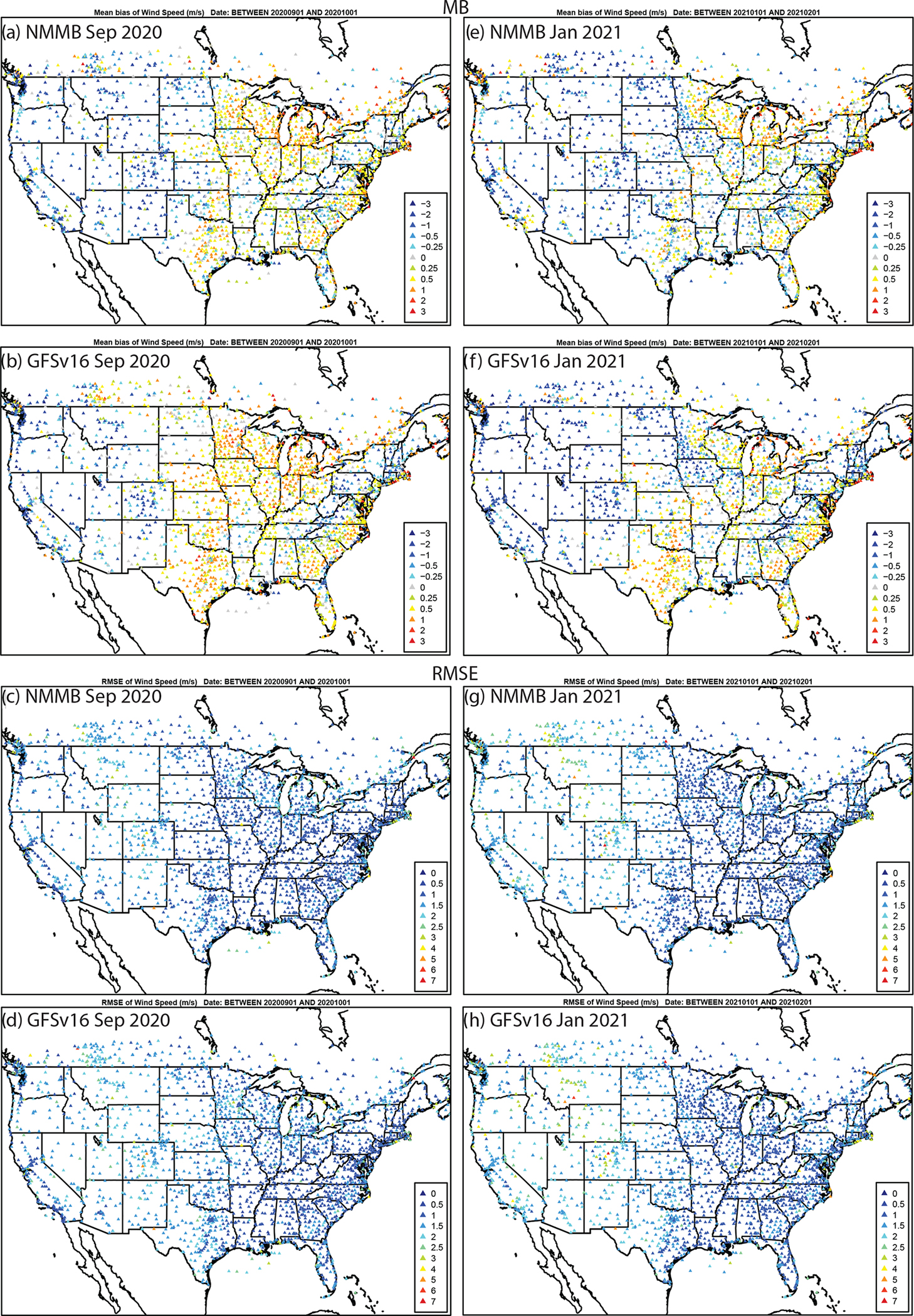
The same as in Fig. 6 but for WSPD10 (m s^−1^).

There are regions of higher RMSE for T2 and Q2, and lower and degraded ACC (Figs. S7–S8) for GFSv16 compared to NMMB, especially in the southern and western CONUS regions during September. The spatial patterns and magnitudes of WSPD10 bias and error are similar between GFSv16 and NMMB (Fig. 8); however, the higher WSPD10 for GFSv16 in the southern and central CONUS leads to a shift from negative to positive biases from Texas northward to North Dakota, especially during September. The WSPD10 RMSE is higher (Fig. 8) and the ACC is also lower/degraded (Fig. S9) for GFSv16 in those regions, but otherwise the GFSv16 and NMMB have similar performance for WSPD10. The day 1 forecast model performance (MB, RMSE, and ACC) for 10 m wind direction (WDIR10) is similar between NMMB and GFSv16 in both September and January (Figs. S6 and S10).

Overall, the GFSv16 results are favorable for driving the advanced NACC-CMAQ system, with some areas of concern in the degraded TEMP2 and Q2 in the warmer and drier regions, particularly in the south and west CONUS during September. This roughly correlates with warmer/drier top-layer soil conditions in GFSv16 in these regions (Fig. S11), and thus land surface and soil data assimilation and model improvement in GFSv16 is an active area of focus at NOAA. The widespread dry bias in GFSv16 appears to be persistent, as an independent evaluation of August 2019 demonstrated a very similar spatial pattern and magnitude of Q2 under-predictions in the eastern half of CONUS compared to the METAR network (not shown).

The GFSv16-driven NACC-CMAQ system extends out to a 72 h forecast. Hence, there is a question of how the day 1 and 2 forecasts perform for NMMB vs. GFSv16 in the eastern (*<* 100° W) and western (> 100° W) US and how a day 3 forecast extension also affects the GFSv16 diurnal and statistical model performance. The GFSv16/NACC diurnal patterns of standard deviation, error, and bias for TEMP2, Q2, and WSPD10 are very similar to each other for days 1–3 (Figs. S12–S14). While there is a slight increase in error and decreased correlation (*R*), the relevant statistical metrics (e.g., MB, NMB, RMSE, and *R*) do not change appreciably from day 1 to 3 for both September and January (Tables S3–S4). This lends confidence in the utility of using the updated GFSv16 meteorology to drive a 72 h air quality forecast in NACC-CMAQ.

The day 1 diurnal statistics highlight both similar and contrasting TEMP2 and Q2 patterns for NMMB vs. GFSv16 in the eastern and western CONUS (Figs. S12–S13). In September (Fig. S12a), NMMB has higher error and positive TEMP2 (i.e., warm) bias in eastern CONUS during morning hours and lower error with a slight cool bias in the afternoon and evening, while GFSv16 shows slightly overpredicted TEMP2 during most hours of the day in the east. Over the western CONUS, there are larger diurnal TEMP2 differences that include small oscillating TEMP2 biases (about zero) for NMMB, along with distinctly large warm biases during all daytime hours for GFSv16 in the west. There are larger error and negative Q2 (i.e., drier) biases for GFVSv16 compared to NMMB in eastern and western CONUS (Fig. S13a). In January, the TEMP2 and Q2 diurnal statistical patterns are similar for NMMB and GFSv16 in both the eastern and western CONUS; however, the GFSv16 daytime hours have slightly higher error and warmer and drier biases compared to NMMB (Figs. S12b and S13b).

**Figure 9.**
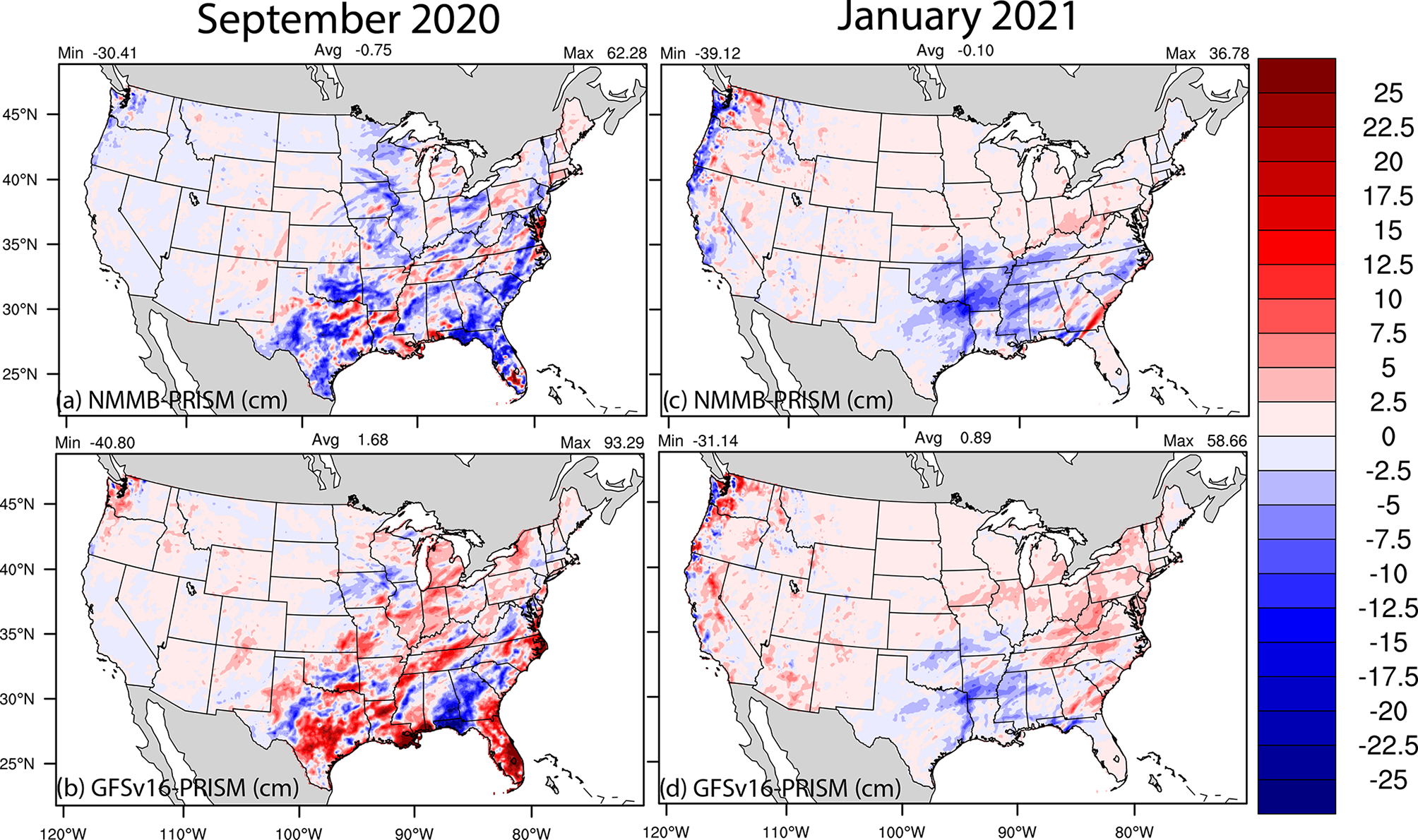
Average day 1 (0–24 h) forecasted total PRECIP (cm) biases (Predicted-PRISM) for NMMB (**a, c**) and GFSv16 (**b, d**) during (**a**)–(**b**) September 2020 and (**c**)–(**d**) January 2021.

The total PRECIP is generally higher in GFSv16 compared to NMMB toward the east (Fig. 5), which leads to larger overpredictions on average in the CONUS compared to PRISM (Fig. 9). GFSv16 has a positive PRECIP bias on average in the CONUS, NMMB has a negative bias, and there is a relatively large difference in the spatial patterns between NMMB and GFSv16 for September compared to January. The difference is impacted by higher convective activity during late summer and early fall in September compared to winter in January (not shown). Further analysis indicated that generally heavier PRECIP in GFSv16 reduces the predicted PM_2.5_ concentrations via wet deposition (not shown) in the east and southeast and in parts of the west and northwest compared to NMMB.

**Figure 10.**
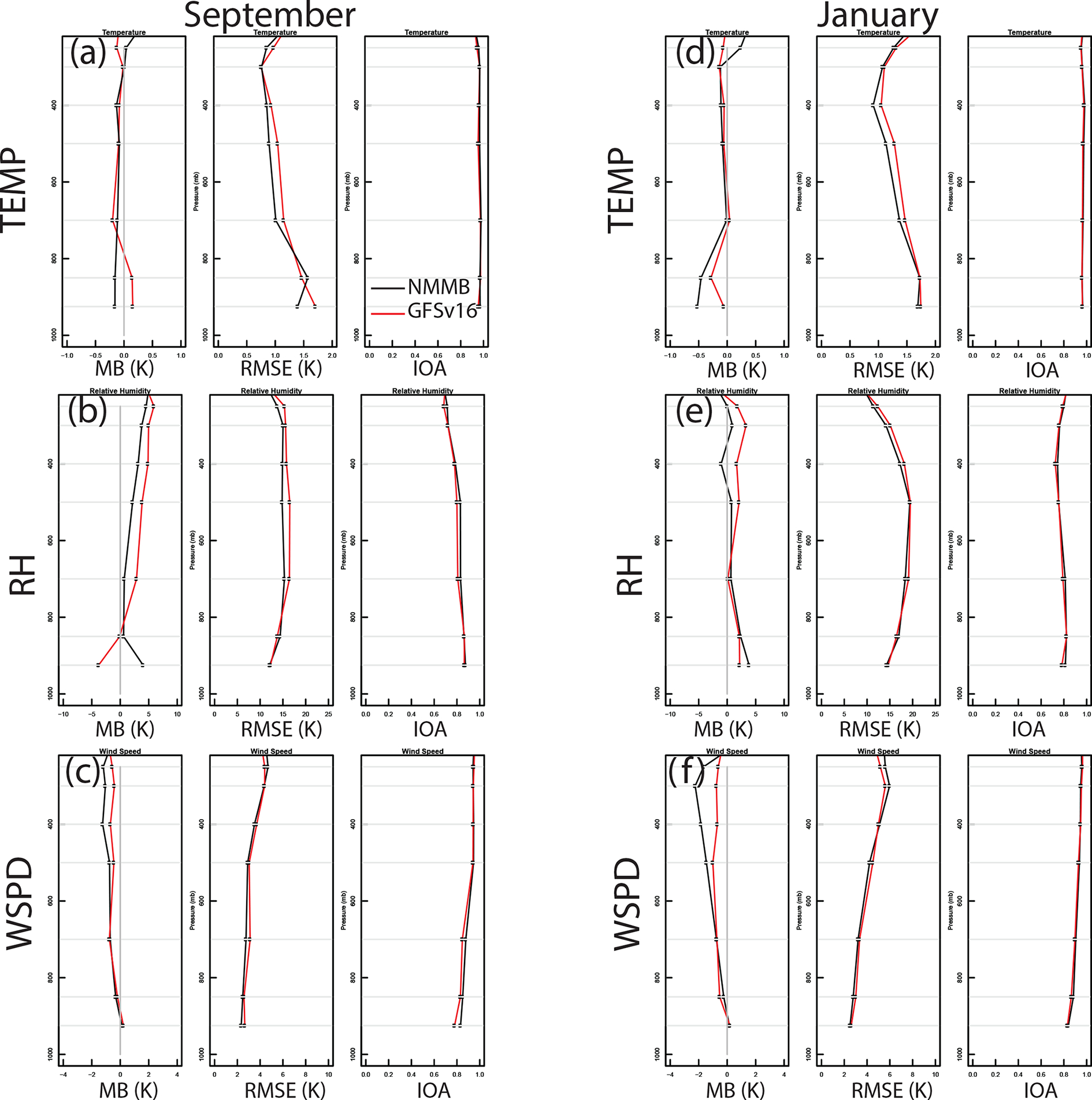
September 2020 (**a, b, c**) and January 2021 (**d, e, f**) vertical (1000–250 mb) temperature (TEMP), relative humidity (RH), and wind speed (WSPD) statistics (MB, RMSE, and IOA) for NMMB (black) and GFSv16 (red) against an average for select RAOB sites in the CONUS. Figure S15a shows the specific RAOB site profiles, and Figs. S18–S19 provide their relative locations.

Comparisons of the model vertical profile statistics (i.e., MB, RMSE, and IOA) for TEMP, RH, and WSPD against an average of select RAOB observations across the CONUS indicate that the GFSv16 (NACC) performs consistently with the operational NMMB (NAQFC) column (Fig. 10; IOA nearly identical at ~ 0.8–0.9). GFSv16 is warmer and drier than NMMB in the model layers near the surface (> 850 mb), especially in September; however, GFSv16 has a moister atmospheric column with higher wind speeds compared to NMMB above the surface and in the free troposphere (*<* 850 mb). Figures S15–S17 show the spatial variability across the different RAOB sites used in the average for Fig. 10. Analysis of the column (1000–250 hPa) average for all CONUS RAOB sites across CONUS indicate that GFSv16 has a predominantly cooler and moister atmospheric column in September, despite being strongly warmer and drier near the surface (Figs. S18–S19).

### 4.3 Emissions analysis

The updated NEIC2016v1 emissions in NACC-CMAQ are lower compared to the NEI2014v2 emissions used in the operational NAQFC for all major species, except for NH_3_ (Table 2), as the NEIC2016v1 includes updated data sources and model projections that have generally decreasing emissions compared to the NEI2014v2 (NEIC, 2019).

**Figure 11.**
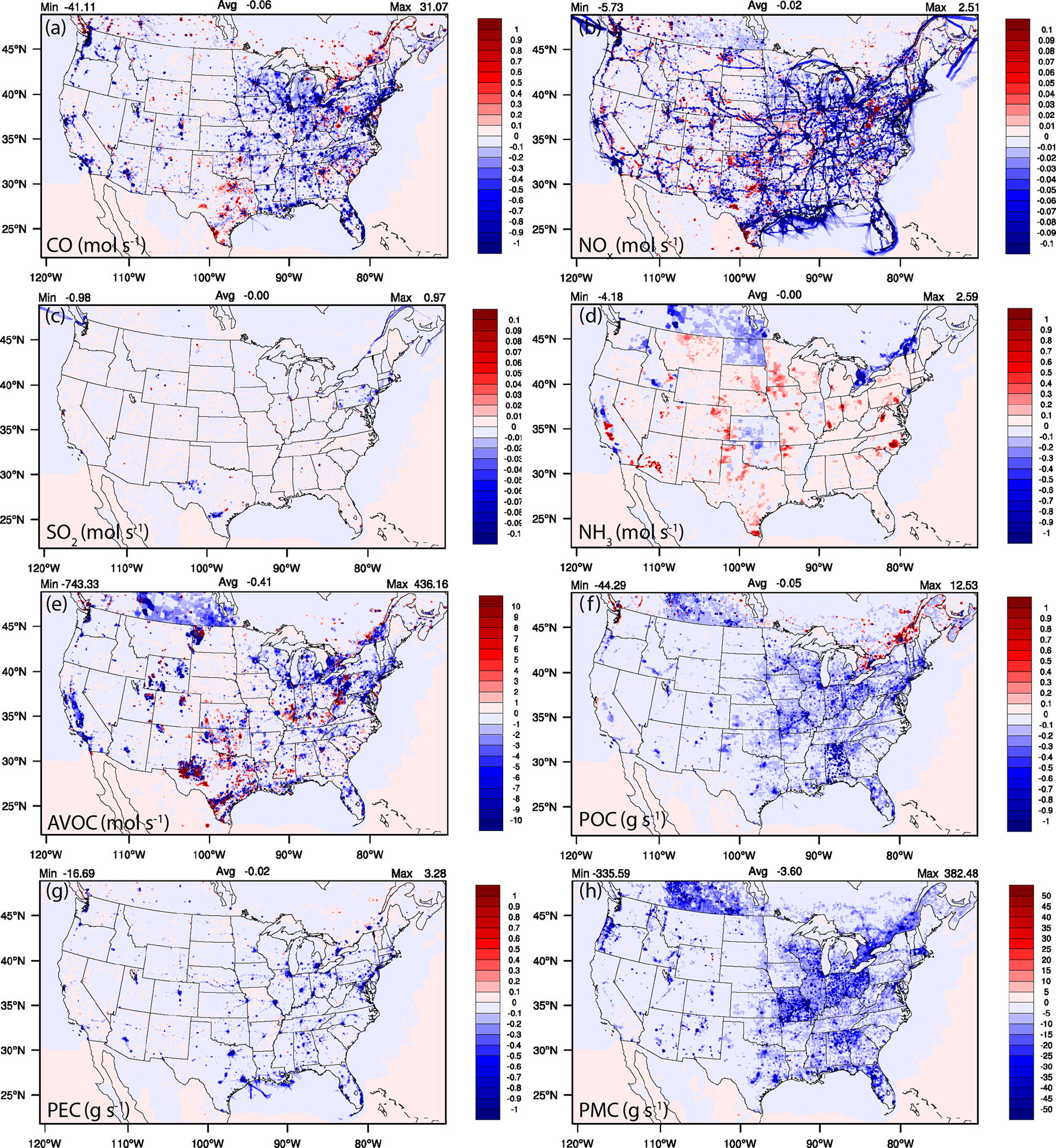
September 2020 average spatial difference plots for NEIC2016v1–NEI2014v2 combined 2-D area and mobile emissions. Figure S20 shows very similar emission changes for January 2021.

The spatial emission changes show widespread decreases in the 2-D area and mobile emissions near the major urban cities for CO and NO_*x*_ and across the major interstates and railways for NO_*x*_ (Fig. 11a–b).

The spatial variability in NO_*x*_ emission changes, however, are impacted by changes in a number of on-road inputs including vehicles miles traveled, age distribution, and speeds, which caused some emissions to go up or go down depending on the specific counties. The NO_*x*_ emissions variability is also impacted by national increases in railway levels and fuel use, while at the same time being impacted by changes to fuel efficiency and cleaner engines for both passenger and commuter trains. There are relatively minor area and mobile changes in SO_2_ (Fig. 11c), with some exceptions in the east-northeast; however, there are widespread increases in NH_3_ emissions driven by changes to the livestock counts and updated fertilization methods and inputs found in the NEIC2016v1 (Fig. 11d). Changes in non-point oil and gas production, exploration, and emission factors generation, as well as changes to updated activity and data sources for commercial cooking, residential fuel combustion, and industrial/commercial/institutional (ICI) fuel combustion impact the anthropogenic VOC (AVOC) area emission changes (Fig. 11e). The widespread and spatially consistent decreases in particulate organic carbon (carbon only) ≤ 2.5 *μ*g (POC) and PMC (defined as coarse PM > 2.5 *μ*g and ≤ 10 *μ*g) are due to decreasing fugitive dust sources (Fig. 11f and h), with the exception of the St. Lawrence River valley, that has both increases in POC and AVOC (e.g., formaldehyde; not shown) emissions in the NEIC2016v1. Updated appliance counts and residential wood combustion estimates affect the particulate elemental carbon ≤ 2.5 *μ*g (PEC) area emission decreases (Fig. 11g).

**Figure 12.**
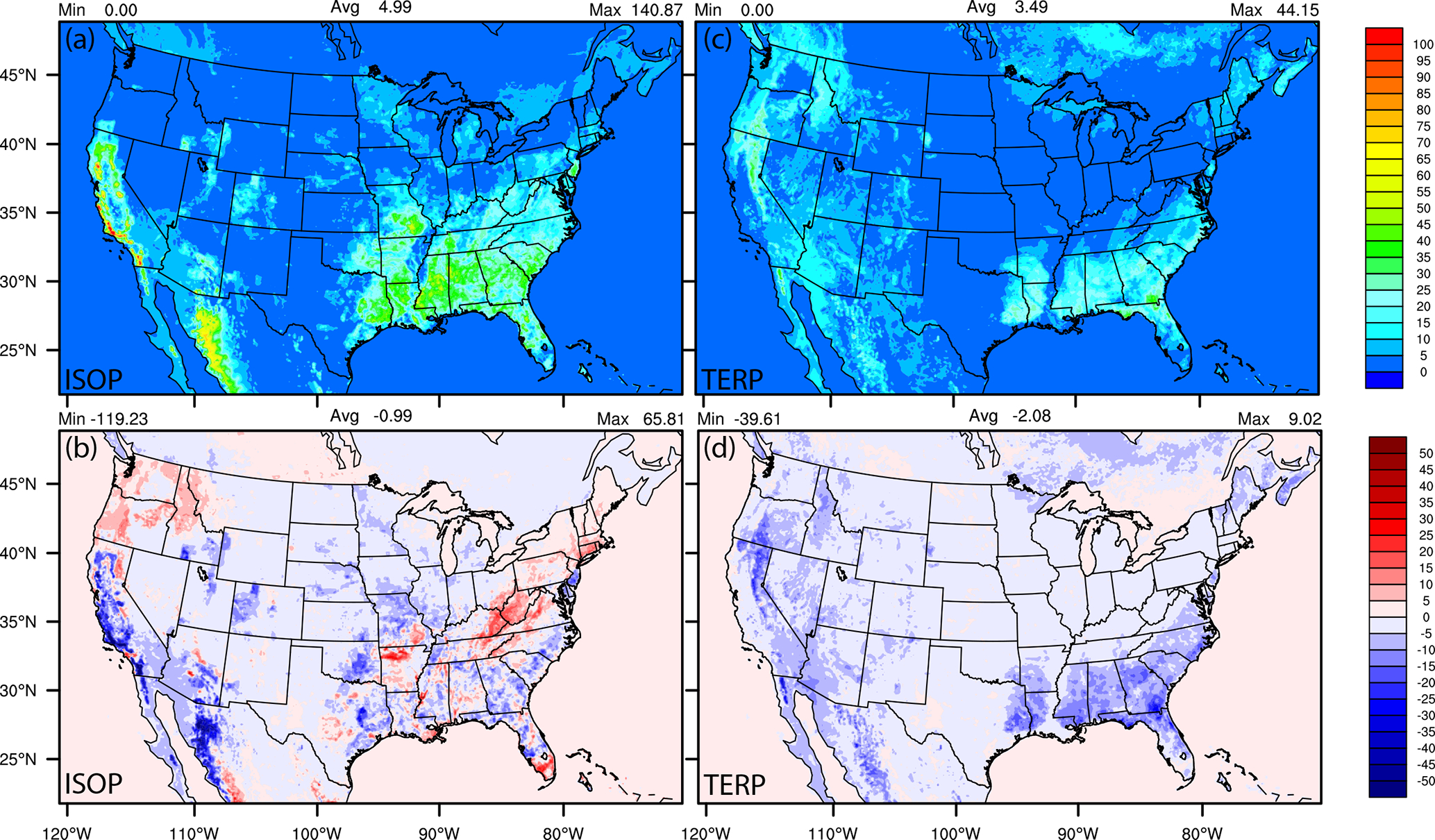
September 2020 average isoprene (ISOP) and terpene (TERP) emissions (**a, b**) in the prior NAQFC with BEISv3.1.4 and the absolute differences (**c, d**) between NACC-CMAQ (with BEISv3.6.1) and NAQFC.

There are also biogenic emissions differences due to the updated inline BEISv3.6.1 and BELD5 in NACC-CMAQ (Table 2) and due to the impacts of NMMB (prior NAQFC) vs. GFSv16 (NACC) meteorology on BEIS calculations (Fig. 12).

The lower GFSv16 temperatures near many of the highly vegetated regions of the CONUS in September (Fig. 4b) decrease the isoprene (ISOP) and terpene (TERP) emissions, with some notable localized ISOP emission increases due to larger relative increases in downward solar radiation at the surface (GSW; Fig. 5b) and resulting photosynthetic active radiation (PAR; not shown). The differences are also impacted by the derivations of leaf temperatures in the updated BEISv3.6.1 and BELD5 in NACC-CMAQ compared to the BEISv3.14 and BELD3 in the prior NAQFC (see discussion in Sect. 2.2). Hence, the differences in spatial variability between ISOP and TERP emission changes stem from both differences in the locations of their relative maxima and from the different algorithms for temperature and light dependencies in BEIS. The GFSv16 (NACC) performs very similarly to NMMB (prior NAQFC) for GSW at the surface compared against BSRN-SURFRAD observations in the CONUS, with a slightly larger overprediction in the late afternoon at some sites (Figs. S21 and S22). The relatively low ISOP and TERP emissions in NACC-CMAQ will effectively lower the ground-level O_3_ and contribution of secondary organic aerosol (SOA) formation to PM_2.5_ compared to the prior NAQFC, particularly in the southeast and parts of the western CONUS in the late summer and early fall.

### 4.4 Air quality analysis

**Figure 13.**
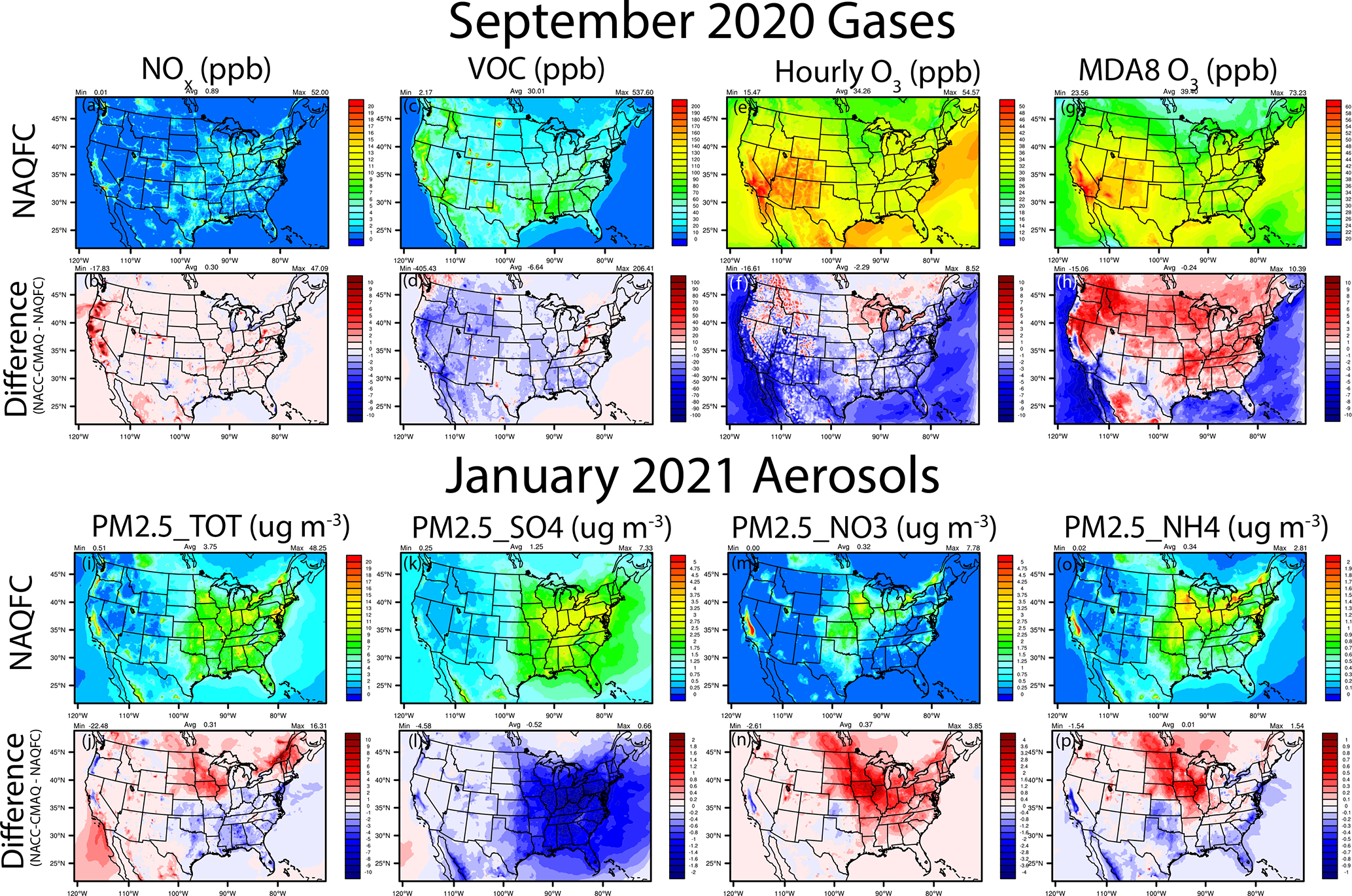
Average September 2020 NO*x*, total VOCs, hourly O_3_, and MDA8 O_3_ and January 2021 PM_2.5__TOT, PM2.5_SO4, PM2.5_NO3, and PM2.5_NH4 for the prior NAQFC and the absolute differences for NACC-CMAQ–NAQFC.

Here we focus on analysis of NACC-CMAQ predictions of gaseous O_3_ for the late summer and early fall (September 2020) and PM_2.5_ concentrations during the winter (January 2021) as concentrations are relatively high for the pollutant’s respective seasons. During the late US ozone season in September 2020, a large majority of the local NO_*x*_ concentration increases in NACC-CMAQ (Fig. 13a–b) correlate with areas of NO_*x*_ emissions increases in the NEIC2016v1 compared to the NEI2014v2 (Fig. 11b). An exception is the large NO_*x*_ increases in the far west (e.g., California and Oregon) that stem from gaseous NO_*x*_ emissions from strong wildfires that are captured by the GBBEPx in NACC-CMAQ (Table 1) but are excluded from the prior NAQFC wildfire emissions system (Table S2).

The increases in NO_*x*_ concentrations and enhanced night-time O_3_ titration, widespread decreases in total VOC concentrations due to both anthropogenic and biogenic VOC emission decreases in NACC-CMAQ, GFSv16-meteorology impacts (e.g., higher PBLH), and updated CMAQv5.3.1 chemistry and transport lead to widespread decreases in hourly O_3_ when averaged over all hours (Fig. 13e–f). Regions of higher NO_*x*_ emissions, overall drier (i.e., widespread lower Q2) conditions, and stronger mid- to late-afternoon solar radiation reaching the surface (i.e., widespread lower CFRAC) (see Figs. 4–5 and S21–22) lead to enhanced daytime O_3_ formation, which is shown in the widespread increases in the maximum daily 8 h average (MDA8) O_3_ for NACC-CMAQ (Fig. 13g–h). This is particularly true for the strongly NO_*x*_-limited conditions across much of the western CONUS, where the MDA8 O_3_ increases are impacted by large increases in wildfire NO_*x*_ emissions in GBBEPx and VOC decreases (anthropogenic+biogenic but no wildfire VOC emission impacts) in NACC-CMAQ. These effects subsequently impact the ozone NO_*x*_-VOC sensitivity regime that enhances the NO_*x*_-saturated (i.e., VOC-limited) conditions in this case (Fig. S24). There are exceptions, with MDA8 O_3_ decreases in the west, including western Oregon, the San Joaquin Valley in California, and regions of the southwestern CONUS, all of which are strongly VOC-limited (Fig. S24). These regions are further impacted by the VOC decreases and further NO_*x*_ saturation from wildfire emissions in some locations of the west. Although outside the scope of this work, we also found that the NACC/GFSv16-CMAQ system yields reasonable results when comparing fire-enhanced O_3_ and PM_2.5_ concentrations to aircraft measurements during the 2019 Fire Influence on Regional to Global Environments and Air Quality (FIREX-AQ) field campaign (https://csl.noaa.gov/projects/firex-aq/, last access: 5 April 2022) (not shown). The widespread decreases in both the hourly and MDA8 O_3_ over all oceanic regions in the domain are driven by the updated halogen (e.g., bromine and iodine chemistry) mediated O_3_ loss in NACC-CMAQ, which can reduce annual mean surface ozone over seawater by 25 % (Sarwar et al., 2019).

There are both relatively large increases (north, northeast, and west) and decreases (south, southeast, and parts of the west) for winter (January 2021) total PM_2.5_ (PM25_TOT) in the CONUS for NACC-CMAQ compared to NAQFC (Fig. 13i–j). The decreases in inorganic PM25_TOT in the east and southeast are dominated by decreases in particulate sulfate (PM25_SO4) and ammonium (PM25_NH4), while the increases in the northern central and eastern CONUS are driven by increases in particulate nitrate (PM25_NO3) and PM25_NH4. Further analysis indicates that the widespread decreases in PM25_SO4 (strongest in the east) are driven strongly by widespread lower CFRAC in GFSv16 (Fig. 4o–p) and lower aqueous-phase oxidation in CMAQ (not shown). There are also contributions from decreased SO_2_ emissions found in some CONUS regions for NACC-CMAQ (e.g., the northeast; Fig. 11c). Additional consumption of inorganic sulfate as secondary isoprene epoxydiol (IEPOX) organosulfates are formed in the updated AERO7 aerosol mechanism in NACC-CMAQ (Table 1; Pye et al., 2013, 2017), and these further contribute to the PM25_SO4 decreases. The higher total PRECIP for NACC-CMAQ (Fig. 5) also leads to lower PM25_TOT in the eastern and southeastern regions.

The largest PM25_TOT increases in the northern central CONUS are primarily driven by enhanced ammonium nitrate formation, PM25_NO3, and PM25_NH4, which are influenced by increases in NH_3_ emissions (Fig. 11) and the inclusion of BIDI-NH3 fluxes in NACC-CMAQ (Table 1). BIDI-NH3 in NACC-CMAQ allows for inline calculation of the diurnal pattern of both NH_3_ evasion (emission) and deposition, while the prior NAQFC only includes deposition. Consequently, BIDI-NH3 in NACC-CMAQ generally increases ambient $NH_{4}^{+}$ and $NO_{3}^{-}$ aerosol concentrations (Bash et al., 2013; Pleim et al., 2019) compared to the prior NAQFC.

There are also contributions to the increased PM25_TOT from organic carbon sources (Fig. S25; PM25_OC), especially in the northeastern portion of the domain that include the St. Lawrence River valley region. This is in part due to enhanced anthropogenic VOC emissions in NEIC2016v1 (Fig. 11e, e.g., formaldehyde; not shown) and enhanced AERO7 secondary organic aerosol formation in this region for NACC-CMAQ (not shown). There are also small PM25_EC contributions to the PM25_TOT decreases in the east and increases in the west for NACC-CMAQ (Fig. S25), which are mainly due to decreases in anthropogenic PEC emissions in the east (Fig. 11g) but also stem from contributions of relatively small GBBEPx PM emissions in the west (not shown). The prior NAQFC does not include biomass burning smoke emissions during the month of January.

### 4.5 Air quality evaluations and metrics

**Figure 14.**
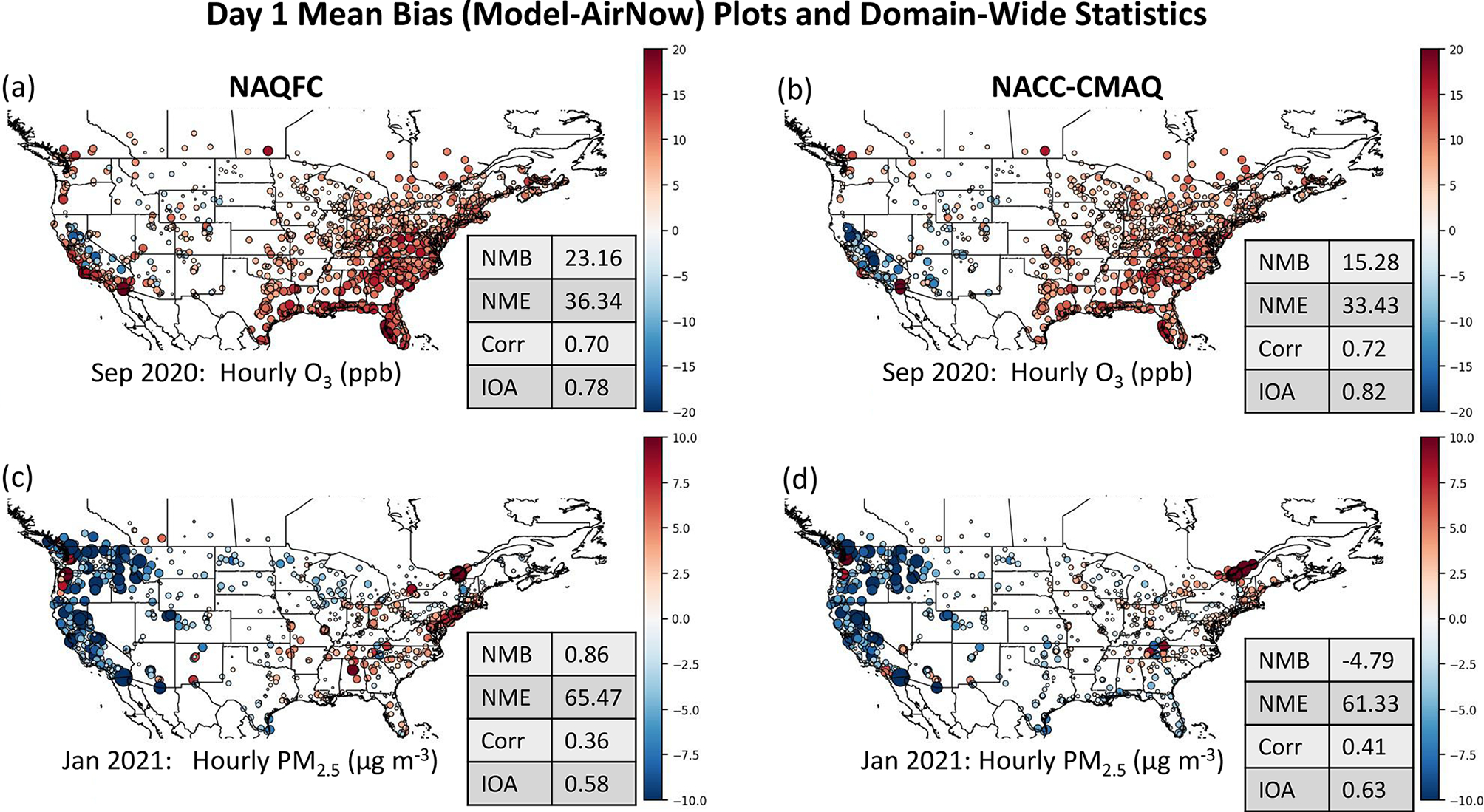
Day 1 forecast mean bias plots (model-AirNow) for the current operational NAQFC (**a, c**) and NACC-CMAQ (**b, d**) hourly O_3_ (a, **b**) and PM_2.5_ (**c, d**) in (**a**)–(**b**) September 2020 and (**c**)–(**d**) January 2021. Average domain-wide statistics are shown in the tables on the bottom right of each panel.

Evaluation of NACC-CMAQ shows overall improvement in the spatial MB of hourly O_3_ (September) and PM_2.5_ (January) against the AirNow network across CONUS (Fig. 14). There are clear reductions in the NAQFC overpredictions of O_3_ and PM_2.5_ in the east, and overall reduction in NME, and overall improved correlation (*R*) and IOA for NACC-CMAQ. There are also reduced overpredictions in the west for O_3_ in September. The shifts to lower concentrations result in larger domain-wide average PM_2.5_ under-predictions for NACC-CMAQ compared to the prior NAQFC (cf. Fig. 13 above); however, the improvements in *R* and IOA for NACC-CMAQ are substantial. The MDA8 O_3_ spatial MB evaluation against AirNow behaves similarly to NAQFC, with slight degradation in the model performance statistics because of areas of higher overpredictions in the eastern US due to reasons discussed above for enhanced daytime O_3_ formation in NACC-CMAQ (Fig. S26).

The day 2 forecasts have similar spatial model performance and statistics, with improved hourly O_3_ and PM_2.5_ model performance (Fig. S27) and slightly higher MDA8 O_3_ overpredictions in the east for NACC-CMAQ (Fig. S28). The consistent model performance for day 3 also shows utility in extending to 72 h air quality forecasts in the advanced NACC-CMAQ system (Figs. S29–S30). There is, however, a more notable degradation in skill for the day 3 forecast of PM_2.5_ compared to O_3_ in NACC-CMAQ (compare Figs. 14 and S29).

**Figure 15.**
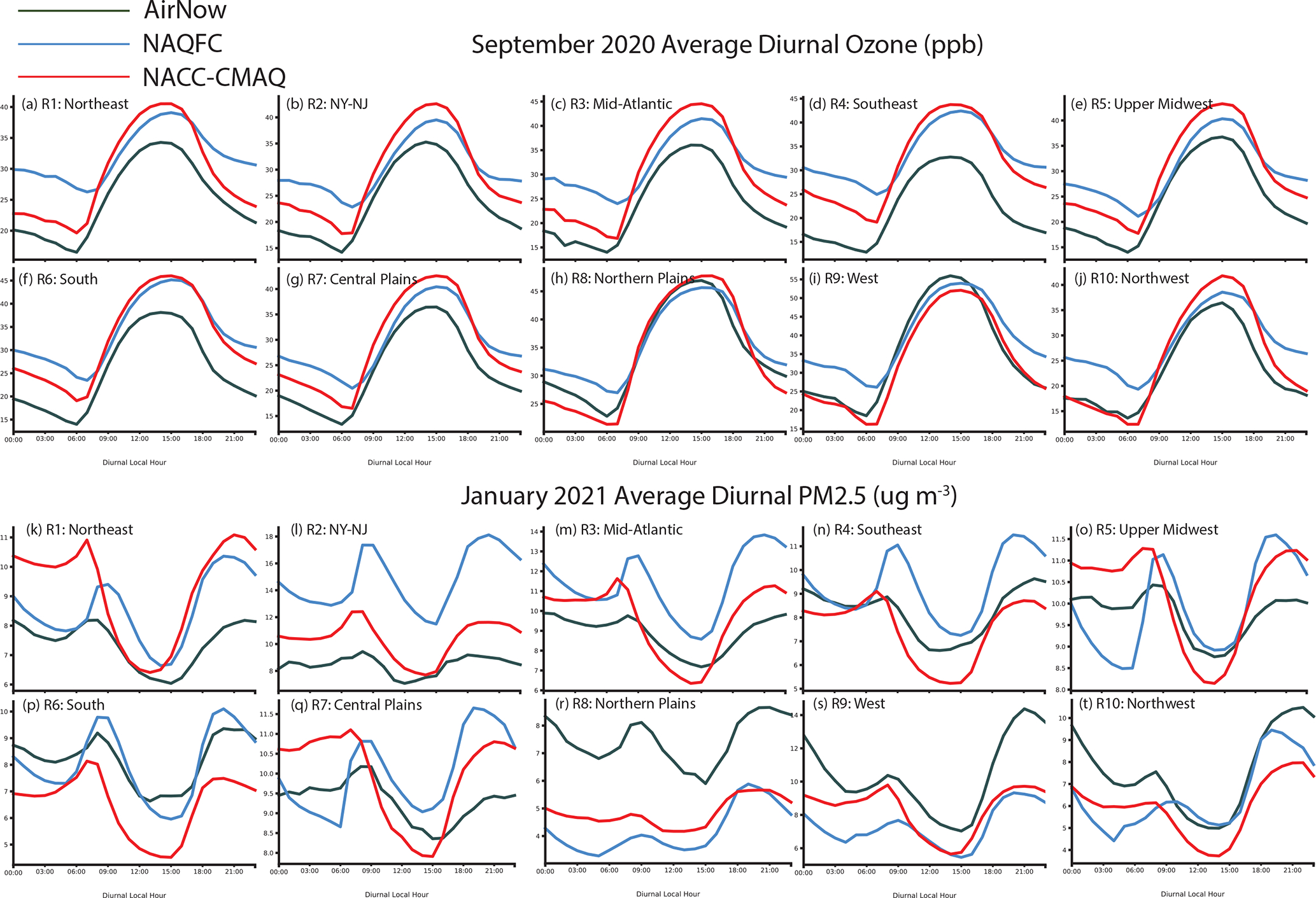
Average September 2020 O_3_ (top) and January 2021 PM_2.5_ (bottom) diurnal patterns for NAQFC (blue), NACC-CMAQ (red), and AirNow observations (green) for different regions in the CONUS. The regions are based on https://www.epa.gov/aboutepa/regional-and-geographic-offices (last access: 5 April 2022).

There is significant improvement in the average O_3_ and PM_2.5_ diurnal patterns for each CONUS region other than higher daytime O_3_ peaks for NACC-CMAQ compared to prior NAQFC (Fig. 15a–i). This is reflected in the improved *R* and IOA over the CONUS on average for NACC-CMAQ (Fig. 14a–b). There is improved day-to-night O_3_ transition, i.e., a sharper slope or cutoff of daytime O_3_ formation, which leads to lower night-time O_3_ mixing ratios in NACC-CMAQ that agree better with AirNow observations for all CONUS regions.

The NACC-CMAQ PM_2.5_ diurnal pattern is also more consistent with AirNow for most CONUS regions (Fig. 15k–t), which is supported by improved *R* and IOA (Fig. 14c–d). There are, however, some regions (e.g., the northeast, south, and northwest) that the prior NAQFC shows better diurnal performance in this case.

Overall performance evaluations of hourly O_3_ in each CONUS region show predominantly improved statistics for NACC-CMAQ, with increased *R* and IOA for all regions (Table 3). Comparisons of the NMB, NME, and *R* against statistical benchmark values for photochemical models based on Emery et al. (2017) indicate that both the prior NAQFC and NACC-CMAQ are within specified criteria for hourly O_3_ in most regions, except for relatively large NMB values in the west and northwest regions. The increased hourly O_3_ under-predictions in NACC-CMAQ degrades the NMB to fail to meet the benchmark in the west but improves the NMB to fall within criteria in the northwest region.

The higher MDA8 O_3_ in NACC-CMAQ degrades its regional NMB, NME, and *R* performance slightly compared to the prior NAQFC (Table 4), but *R* and IOA illustrate improvements for most regions, in some cases substantially for *R* (e.g., northeast, southeast, the upper Midwest, and the central Great Plains). The higher daytime O_3_ overpredictions by NACC-CMAQ in much of CONUS result in higher NMB and NME values that fall outside of the Emery et al. (2017) benchmark criteria. These remain a concern for both the prior NAQFC and NACC-CMAQ, and efforts are underway to address the persistent daytime O_3_ overprediction in the summer, particularly in the eastern US (see Fig. 14a–b and further discussion in Sect. 5).

There are substantial improvements in the overall statistical PM_2.5_ performance for NACC-CMAQ, especially for *R* and IOA in most CONUS regions. In many regions where the prior NAQFC falls outside of photochemical criteria values (Emery et al., 2017), NACC-CMAQ shows significant improvement to fall within the criteria. This demonstrates a substantial improvement in the accuracy of the NACC-CMAQ system for PM_2.5_ predictions (outside of major wildfires), attributed to the scientific advancements described above.

The day 2 forecast comparisons of the prior NAQFC and NACC-CMAQ regional statistics are similar to day 1, and the day 3 forecast extension for NACC-CMAQ has utility as its O_3_ and PM_2.5_ statistics predominantly fall within the benchmark criteria in most regions (Tables S5–S10).

## 5 Conclusions and path forward

An advanced National Air Quality Forecasting Capability (NAQFC) was developed and evaluated using NOAA’s FV3-based Global Forecast System version 16 (GFSv16) as the driving meteorology for a state-of-the-science Community Multiscale Air Quality (CMAQ) model version 5.3.1. A key component of this new system is the development of the NOAA-EPA Atmosphere Chemistry Coupler (NACC), which forms the bridge between the GFSv16 meteorological fields and the CMAQ inputs for improved chemical predictions (i.e., NACC-CMAQ). Such advancements of the NACC-CMAQ system include high-resolution satellite vegetation inputs, with a rapid-refresh VIIRS greenness vegetation fraction and VIIRS climatological leaf area index, as well as additional soil data inputs to an improved windblown dust (FENGSHA) algorithm in CMAQ. The anthropogenic, biogenic, and wildfire emissions in NACC-CMAQ are also updated compared to the prior NAQFC, and for the first time the forecasting model calculates inline bidirectional NH_3_ fluxes. NACC-CMAQ also ingests novel smoke and dust aerosols at its lateral boundaries dynamically from the NOAA operational GEFS-Aerosols model. Finally, the NACC-CMAQ system extends the air quality forecast from 48 to 72 h and provides scientific advances in atmospheric chemistry modeling to state and local forecasters out to 3 d. The additional day of forecast guidance could aid decision makers to prepare citizens for localized air quality conditions that could adversely affect public health.

Results of the NACC-CMAQ system during recent late summer (September 2020) and winter (January 2021) months show significant changes in both meteorological and chemical predictions compared to the prior NAQFC. The GFSv16 for NACC-CMAQ has a persistently large dry bias (lower Q2) and larger RMSE across much of CONUS in late summer compared to NMMB (i.e., prior NAQFC), which likely stems from excessively dry soil conditions in GFS. GFS is generally cooler in the east and warmer in the west for surface temperature (TEMP2) compared to NMMB, but the overall MB and RMSE are more similar between the models compared to that for Q2. The GFS has a relatively similar planetary boundary layer height (PBLH) at night, but the PBLH in GFSv16 (NACC-CMAQ) is consistently deeper during the daytime peak hours compared to the prior NAQFC.

The differences in surface characteristics, meteorology, and both anthropogenic and natural emissions are driving factors for distinct atmospheric composition differences, where NACC-CMAQ generally outperforms the prior NAQFC for both hourly O_3_ and PM_2.5_, especially with improved correlation (*R*) and IOA. This agrees well with significant improvements in the diurnal O_3_ and PM_2.5_ patterns for NACC-CMAQ, with distinct improvements in the day-to-night O_3_ slope and cutoff. While similar overall, the maximum daily 8 h average (MDA8) O_3_ is predominantly higher for NACC-CMAQ compared to prior NAQFC, which leads to some forecast degradation due to larger overpredictions of the daytime max O_3_.

The NACC-CMAQ model became the next operational version of the NAQFC at NWS/NOAA on 20 July 2021 and is available on GitHub for continuous integration, future code updates, and potential community research applications. An ongoing comparison and evaluation of the GFSv16/NACC-CMAQ output with a GFSv16-downscaled Weather Research and Forecasting (WRF) version 4 (Skamarock et al., 2019) and CMAQ application will highlight the potential of NACC-CMAQ to serve as an additional community research tool for air quality applications.

While there are substantial advancements in NACC-CMAQ compared to the prior NAQFC, challenges and limitations remain. One need is to bridge the gap from using a VIIRS LAI climatology to a rapid-refresh methodology, i.e., dynamic methodology (similar to the GVF method here), in NACC-CMAQ. There is also a need to consider shifting the paradigm from using “big-leaf” (i.e., homogeneous single layer of phytomass) assumptions that strongly affect the biosphere–atmosphere exchange processes pivotal to both meteorological and chemical model predictions (refer to Bonan et al., 2021). Simple multilayer canopies have been shown to reduce overpredictions of ground-level surface O_3_ in the summer due to photolysis attenuation and modified vertical turbulence (Makar et al., 2017), which have significant implications for the daytime O_3_ overpredictions in the current and future versions of NAQFC (Figs. 14a–b and S26). We are currently working on similar canopy effects in NACC-CMAQ to reduce the summer O_3_ overpredictions in the east and southeast and parts of western CONUS, where there are relatively continuous vegetation structures and canopies (Fig. 14a–b). Other advancements that are important to improving the future versions of the NAQFC include dynamically updated (and weather-dependent) anthropogenic emission sources and improved treatments of mobile sources (e.g., vehicle-induced turbulence; Makar et al., 2021). Further refinements to the inline windblown dust emissions, wildfire smoke emissions, and other process-based natural emissions sources (e.g., lightning NO) are also needed.

Other future directions include migrating the advanced science in the offline 12 km resolution NACC-CMAQ model to a next-generation, high-resolution (e.g., 3 km) inline modeling framework that fits within NOAA’s strategy for the Unified Forecast System (UFS; https://ufscommunity.org/, last access: 5 April 2022). This model system aims to improve integration of atmospheric composition changes with weather predictions, better resolve finer-scale processes, and advance the rapid-refresh techniques for emissions and surface–atmosphere exchange processes. At this time, NACC-CMAQ also does not use dynamic lateral boundary conditions for trace gases and only has dynamically ingested smoke and dust aerosols at its lateral boundaries from the NOAA operational GEFS-Aerosols model. Current work is underway to use next-generation UFS-based global model systems as updated lateral boundary conditions for trace gases in the future of the NAQFC.

Development and implementation of the NACC-CMAQ model is an important step to (i) advance the NAQFC closer to the state of the science for regional air quality forecasting, (ii) improve community applications of NOAA’s FV3GFS-driven atmospheric composition models, and (iii) facilitate the future development of regional high-resolution inline air quality forecasting systems within the UFS framework at NOAA.

## Supplementary Material

Supplement1

## Figures and Tables

**Table 1.** GFSv16 and NACC-CMAQv5.3.1 model components and configurations. The abbreviation n/a stands for not applicable in this table.

Model attribute	Configuration	Reference

Domain	Conterminous US; Centered on 40° N, 97° W	n/a

Horizontal resolution	12 km	n/a

Vertical resolution	35 Layers from near the surface to about 14 km (∼ 60 hPa)	n/a

Meteorological initial and boundary conditions	FV3GFSv 16	https://nws.weather.gov/ (last access: 5 April 2022)

Chemical ICs and BCs	2006 GEOS-Chem simulation & GEFS-Aerosol dynamic smoke and dust aerosol CLBCs	http://acmg.seas.harvard.edu/geos/ (last access: 5 April 2022) Tang et al. (2021)

Anthropogenic emissions	NEIC 2016v1 platform	NEIC (2019)

Biogenic emissions	Inline BEISv3.6.1 & BELD5	Vukovich and Pierce (2002); Schwede et al. (2005)

Wildfire emissions and plume rise	GBBEPxv3/inline Briggs	https://www.ospo.noaa.gov/Products/land/gbbepx (last access: 5 April 2022); Briggs (1965)

Microphysics	GFDL six-category cloud microphysics scheme	Lin et al. (1983); Lord et al. (1984); Krueger et al. (1995); Chen and Lin (2011, 2013)

PBL physics scheme	sa-TKE-EDMF	Han and Bretherton (2019)

Shallow and deep cumulus parameterization	SAS scheme	Han and Pan (2011); Han et al. (2017)

Shortwave and longwave Radiation	RRTMg	Mlawer et al. (1997); Clough et al. (2005); Iacono et al. (2008)

Land surface model	Noah land surface model	Chen and Dudhia (2001); Ek et al. (2003); Tewari et al. (2004)

Surface layer	Monin–Obukhov	Monin and Obukhov (1954); Grell et al. (1994); Jimenez et al. (2012)

Gas-phase chemistry	CB6	Yarwood et al., 2010

Aqueous-phase chemistry	CMAQ AQCHem updates	Martin and Good (1991); Alexander et al. (2009); Sarwar et al. (2011)

Aerosol module and size	AERO7	Appel et al. (2021)

Other model attributes	– Inline photolysis	Binkowski et al. (2007)
	– Inline bi-directional NH_3_ exchange	Nemitz et al. (2000); Cooter et al. (2010); Massad et al. (2010); Pleim and Ran (2011); Bash et al. (2010, 2013); Pleim et al. (2013, 2019)
	– Inline FENGSHA windblown dust emissions	Fu et al. (2014); Huang et al. (2015); Dong et al. (2016)
	– Inline sea salt emissions	Kelly et al. (2010); Gantt et al. (2015)

**Table 2.** September and January emissions totals (Tg) for the NAQFC CONUS domain.

Emission species	NEI2014v2	NEIC2016v1	Percentage difference

September total (Tg)			

CO	4.69	4.27	−8.9
NO*_x_*	0.92	0.75	−18.1
SO_2_	0.54	0.37	−31.2
NH_3_	0.48	0.59	23.9
AVOC	215.58	195.60	−9.3
POC	0.07	0.05	−26.8
PEC	0.03	0.02	−23.9
PMC	2.03	0.82	−59.3

January total (Tg)			

CO	3.70	3.28	−11.2
NO*_x_*	0.78	0.64	−18.5
SO_2_	0.58	0.38	−34.7
NH_3_	0.10	0.12	18.4
AVOC	182.02	174.05	−4.4
POC	0.08	0.07	−10.8
PEC	0.02	0.02	−16.7
PMC	1.27	0.24	−80.8

**Table 3.** Average September 2020 hourly O_3_ evaluation of the operational NAQFC and NACC-CMAQ day 1 forecasts against the AirNow network in different CONUS regions (based on https://www.epa.gov/aboutepa/regional-and-geographic-offices, last access: 5 April 2022). Statistical benchmark values based on Emery et al. (2017) are also shown for comparison. Following Emery et al. (2017), a > 40 ppb (i.e., daytime) cutoff for hourly O_3_ is applied for the mean observations, mean models, mean bias, and the calculated values of NMB and NME but not for the correlation value (*r*) or index of agreement (IOA). The total number of observation–model pairs is based on all values (i.e., no cutoff). Bold font indicates statistical values outside of the Emery et al. (2017) criteria. Italic font indicates improved NACC-CMAQ performance. Tables S5–S10 provide day 2 and day 3 (NACC-CMAQ only) forecast evaluations.

Day 1 Forecasts	Total no. of pairs	Mean obs (ppb)	Mean mod (ppb)	Mean bias (ppb)	NMB (%)	NME (%)	Corr (*r*)	IOA

Benchmark: Emery et al. (2017)	–	–	–	–	Goal: < ±5 %; criteria: < ±15 %	Goal: < 15 %; criteria: < 25 %	Goal: > 0.75; criteria: > 0.50	–

Region 1 (northeast)								

NAQFC	35 983	46.85	43.55	−3.31	−7.06	15.04	0.61	0.71
NACC-CMAQ			43.44	−3.42	−7.29	15.14	*0.70*	*0.81*

Region 2 (NY–NJ)								

NAQFC	22944	46.68	42.90	−3.77	−8.09	17.88	0.59	0.72
NACC-CMAQ			45.18	*−1.50*	−3.21	*14.27*	*0.72*	*0.81*

Region 3 (mid-Atlantic)								

NAQFC	89069	46.66	44.29	−2.37	−5.09	12.84	0.65	0.73
NACC-CMAQ			45.81	*−0.85*	*−1.83*	13.48	*0.74*	*0.82*

Region 4 (southeast)								

NAQFC	105 858	44.62	45.93	1.31	2.93	13.37	0.61	0.65
NACC-CMAQ			47.99	3.37	7.55	14.91	*0.74*	*0.75*

Region 5 (upper Midwest)								

NAQFC	109 744	46.61	43.84	−2.77	−5.94	13.28	0.69	0.77
NACC-CMAQ			46.59	*−0.03*	*−0.05*	*10.69*	*0.77*	*0.83*

Region 6 (south)								

NAQFC	84 005	48.17	47.18	−0.99	−2.06	13.17	0.68	0.75
NACC-CMAQ			47.81	*−0.36*	*−0.75*	*12.80*	*0.75*	*0.81*

Region 7 (central Great Plains)								

NAQFC	27139	44.98	44.84	−0.14	−0.31	10.45	0.76	0.81
NACC-CMAQ			47.18	2.20	4.90	*9.54*	*0.82*	*0.86*

Region 8 (northern Great Plains)								

NAQFC	51 759	48.97	44.64	−4.32	−8.83	13.89	0.71	0.82
NACC-CMAQ			45.08	*−3.89*	*−7.95*	14.00	*0.72*	*0.85*

Region 9 (west)								

NAQFC	124 051	55.44	50.29	−5.15	−9.29	18.37	0.69	0.79
NACC-CMAQ			46.37	−9.07	**−16.37**	21.78	*0.71*	*0.83*

Region 10 (northwest)								

NAQFC	14139	48.41	39.37	−9.03	**−18.66**	21.59	0.61	0.72
NACC-CMAQ			41.70	−6.71	*−13.86*	*19.91*	*0.66*	*0.81*

**Table 4.** The same as in Table 3 but for MDA8 O_3_. Note that, as discussed in Emery et al. (2017), cutoff values are not applied for MDA8 O_3_.

Day 1 forecasts	Total no. of pairs	Mean obs (ppb)	Mean mod (ppb)	Mean bias (ppb)	NMB (%)	NME (%)	Corr (*r*)	IOA

Benchmark: Emery et al. (2017)	–	–	–	–	Goal: < ±5 %; criteria: < ±15 %	Goal: < 15 %; criteria: < 25 %	Goal: > 0.75; criteria: > 0.50	–

Region 1 (northeast)								

NAQFC	1680	33.05	38.45	5.40	16.35	22.60	0.66	0.73
NACC-CMAQ			38.60	5.55	16.81	21.57	*0.73*	*0.75*

Region 2 (NY–NJ)								

NAQFC	1158	32.79	37.07	4.29	13.08	21.38	0.66	0.76
NACC-CMAQ			39.22	6.44	19.63	23.65	*0.74*	0.75

Region 3 (mid-Atlantic)								

NAQFC	4243	33.85	39.35	5.50	16.24	20.75	0.74	0.77
NACC-CMAQ			41.31	7.46	22.05	24.54	*0.76*	0.75

Region 4 (southeast)								

NAQFC	5076	31.01	40.30	9.29	29.95	31.83	0.64	0.64
NACC-CMAQ			41.06	10.05	32.41	33.40	*0.74*	*0.67*

Region 5 (upper Midwest)								

NAQFC	5210	34.08	37.88	3.80	11.16	18.51	0.75	0.82
NACC-CMAQ			39.89	5.81	17.06	19.94	*0.82*	0.82

Region 6 (south)								

NAQFC	3901	35.65	42.37	6.72	18.84	23.91	0.74	0.77
NACC-CMAQ			43.01	7.35	20.63	24.35	*0.78*	*0.78*

Region 7 (central Great Plains)								

NAQFC	1256	33.37	37.83	4.46	13.36	17.99	0.78	0.82
NACC-CMAQ			39.36	6.00	17.97	19.86	*0.85*	*0.84*

Region 8 (northern Great Plains)								

NAQFC	2379	44.18	43.51	−0.47	−1.07	12.84	0.74	0.85
NACC-CMAQ			44.95	0.78	1.76	*11.78*	*0.79*	*0.88*

Region 9 (west)								

5757	51.03	51.26	0.23	0.44	17.84	0.70	0.82	
NACC-CMAQ			48.03	−3.00	−5.88	18.73	0.68	0.79

Region 10 (northwest)								

NAQFC	698	33.13	35.46	2.33	7.03	25.11	0.63	0.72
NACC-CMAQ			36.66	3.53	10.67	25.58	0.59	*0.74*

**Table 5.** The same as in Table 3 but for 24 h average PM_2.5_. Note that, as discussed in Emery et al. (2017), cutoff values are not applied for 24 h average PM_2.5_.

Day 1 forecasts	Total no. of pairs	Mean obs (ppb)	Mean mod (ppb)	Mean bias (ppb)	NMB (%)	NME (%)	Corr (*r*)	IOA

Benchmark: Emery et al. (2017)	–	–	–	–	Goal: < ±10 %; criteria: < ±30 %	Goal: *<* 35 %; criteria: < 50%	Goal: > 0.70; criteria: > 0.40	–

Region 1 (northeast)								

NAQFC	1261	7.43	8.47	1.04	13.98	42.57	0.77	0.85
NACC-CMAQ			9.39	1.95	26.30	46.17	0.75	0.83

Region 2 (NY–NJ)								

NAQFC	598	8.54	15.39	6.85	80.25	89.21	0.72	0.55
NACC-CMAQ			10.84	2.30	*26.90*	*47.60*	*0.77*	*0.74*

Region 3 (mid-Atlantic)								

NAQFC	1897	9.16	11.95	2.79	30.43	42.57	0.81	0.84
NACC-CMAQ			10.16	1.00	*10.96*	*33.24*	*0.83*	*0.89*

Region 4 (southeast)								

NAQFC	3621	8.45	9.67	1.23	14.53	40.44	0.41	0.62
NACC-CMAQ			7.86	*−0.59*	*−6.98*	*37.19*	*0.48*	*0.67*

Region 5 (upper Midwest)								

NAQFC	3270	9.61	9.79	0.19	1.93	38.09	0.58	0.75
NACC-CMAQ			9.65	*0.04*	*0.46*	*31.42*	*0.72*	*0.84*

Region 6 (south)								

NAQFC	2101	8.39	7.95	−0.44	−5.19	46.68	0.28	0.57
NACC-CMAQ			6.39	−2.00	−23.82	43.30	*0.36*	*0.59*

Region 7 (central Great Plains)								

NAQFC	926	8.67	9.83	1.16	13.41	49.67	0.32	0.58
NACC-CMAQ			8.79	*0.12*	*1.40*	*32.13*	*0.68*	*0.82*

Region 8 (northern Great Plains)								

NAQFC	1790	7.66	4.36	−3.30	−43.13	60.51	0.33	0.55
NACC-CMAQ			4.89	−2.77	*−36.20*	*52.68*	*0.49*	*0.67*

Region 9 (west)								

NAQFC	4118	10.09	7.04	−3.05	−30.27	46.97	0.61	0.74
NACC-CMAQ			7.98	*−2.11*	*−20.89*	50.69	0.56	0.73

Region 10 (northwest)								

NAQFC	3922	7.93	6.86	−1.07	−13.54	78.99	0.20	0.46
NACC-CMAQ			6.33	−1.60	−20.19	*71.73*	*0.23*	*0.49*

## Data Availability

The NACC code is publicly available at https://doi.org/10.5281/zenodo.5507489 (Campbell, 2021a) and via GitHub at https://github.com/noaa-oar-arl/NACC.git (last access: 5 April 2022). The modified version of CMAQv5.3.1 used in the advanced NACC-CMAQ model for the next operational NAQFC is available at https://doi.org/10.5281/zenodo.5507511 (Campbell, 2021b) and via GitHub at https://github.com/noaa-oar-arl/NAQFC (last access: 5 April 2022). The 0.25° FV3-driven Global Forecast System version 16 data (cycled 4× per day) are available in GRIB2 format at https://www.nco.ncep.noaa.gov/pmb/products/gfs/ (NOAA/NWS, 2022a). The hourly GFSv16 data in gridded NetCDF (~ 13 × 13 km globally) format and the Gaussian projection that are directly used to drive NACC-CMAQ are also currently being migrated to the Amazon Web Services (AWS) Cloud for improved NOAA community air quality research applications. The advanced NACC-CMAQ data, i.e., the current operational NAQFC version as of 20 July 2021, are available for operational (https://airquality.weather.gov/, NOAA/NWS, 2022b) and interactive (https://digital.mdl.nws.noaa.gov/airquality/#, NOAA/NWS, 2022c) display from NWS/NOAA. The official NOAA/EMC verification and diagnostics for the NAQFC system are found at https://www.emc.ncep.noaa.gov/mmb/aq/verification_diagnostics/cmaq_verf/ (NOAA/NWS, 2022d).
