# Supplementary material for: Development and evaluation of an advanced National Air Quality Forecasting Capability using the NOAA Global Forecast System version 16
Source: Geosci Model Dev. Author manuscript; Available in PMC 2023 Apr 21. (PMC9157742; doi:10.5194/gmd-15-3281-2022)
Supplement: Supplement1 [file NIHMS1806869-supplement-Supplement1.pdf]

Supplement of Geosci. Model Dev., 15, 3281–3313, 2022
https://doi.org/10.5194/gmd-15-3281-2022-supplement
© Author(s) 2022. CC BY 4.0 License.

Geoscientific Model Development
Open Access

Supplement of

Development and evaluation of an advanced National Air Quality Forecasting Capability using the NOAA Global Forecast System version 16

Patrick C. Campbell et al.

Correspondence to: Patrick C. Campbell (patrick.c.campbell@noaa.gov)

The copyright of individual parts of the supplement might differ from the article licence.

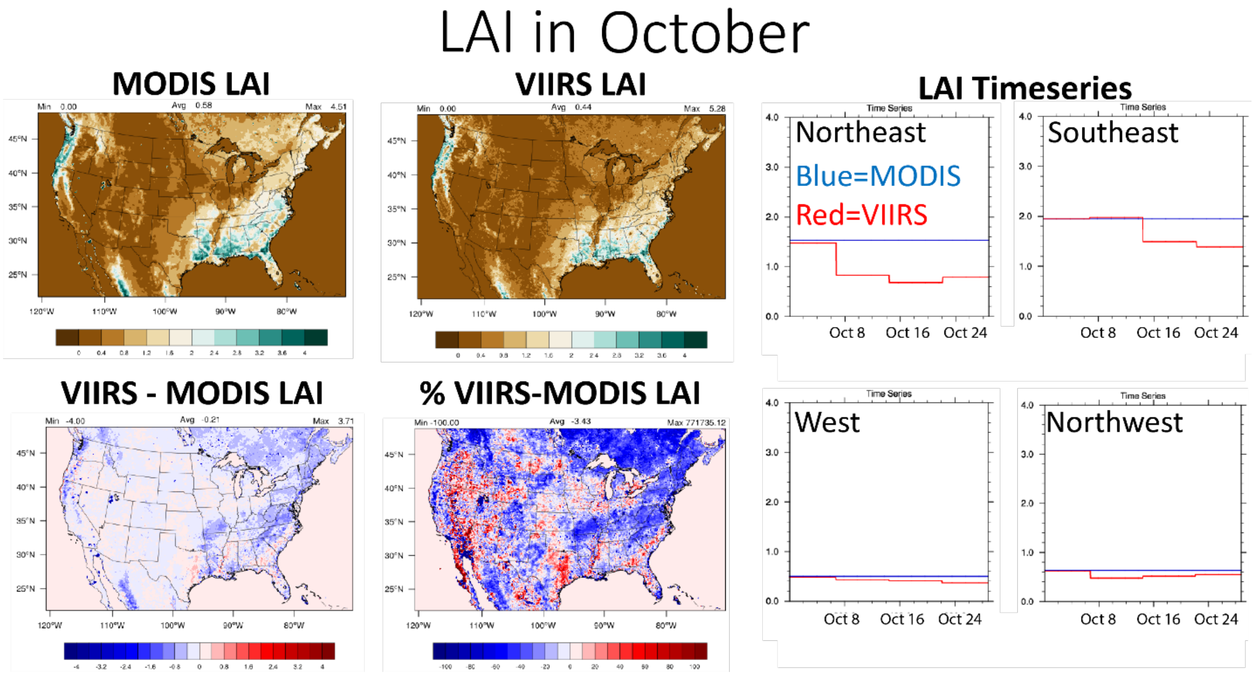

Figure S1. Left: Spatial comparison of October average MODIS-IGBP LAI in 2010 and VIIRS LAI in 2020 (top) and their absolute and relative differences (bottom). Right: Time series comparisons of October average MODIS-IGBP LAI in 2010 and VIIRS LAI 8-day product in 2020 for the northeast, southeast, west, and northwest U.S. regions defined by the U.S. EPA geographic regions.

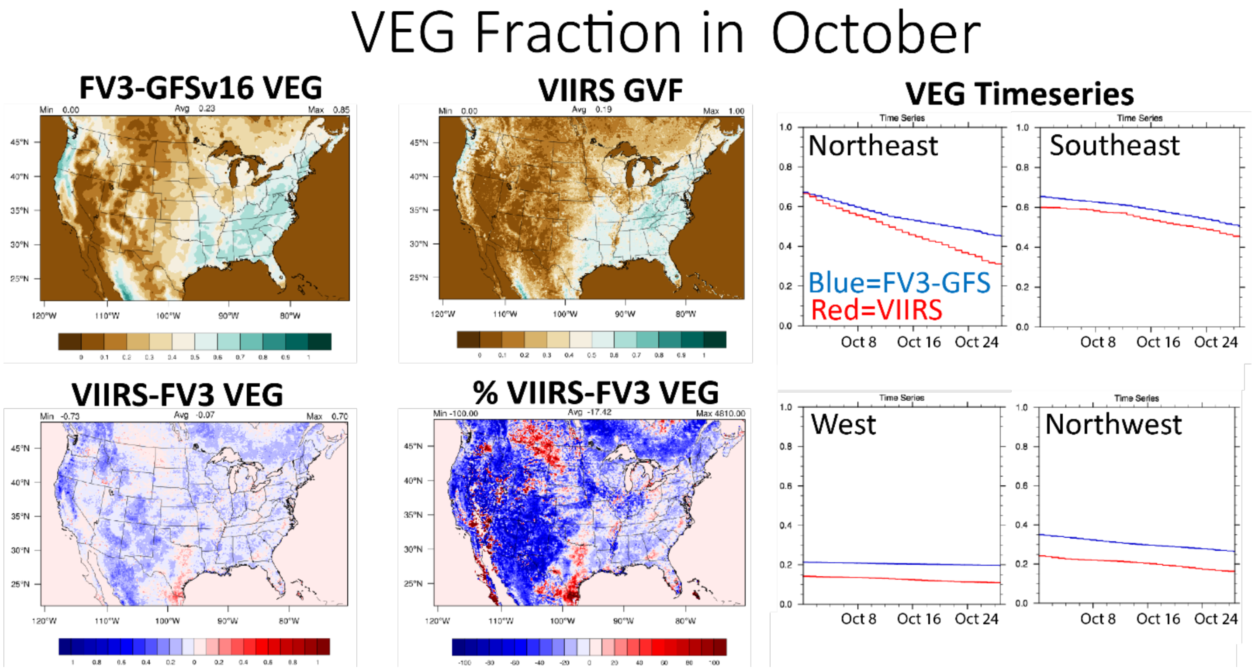

Figure S2. Same as in Figure S1, but for VEG fraction comparisons between GFSv16 and VIIRS average 8-day product.

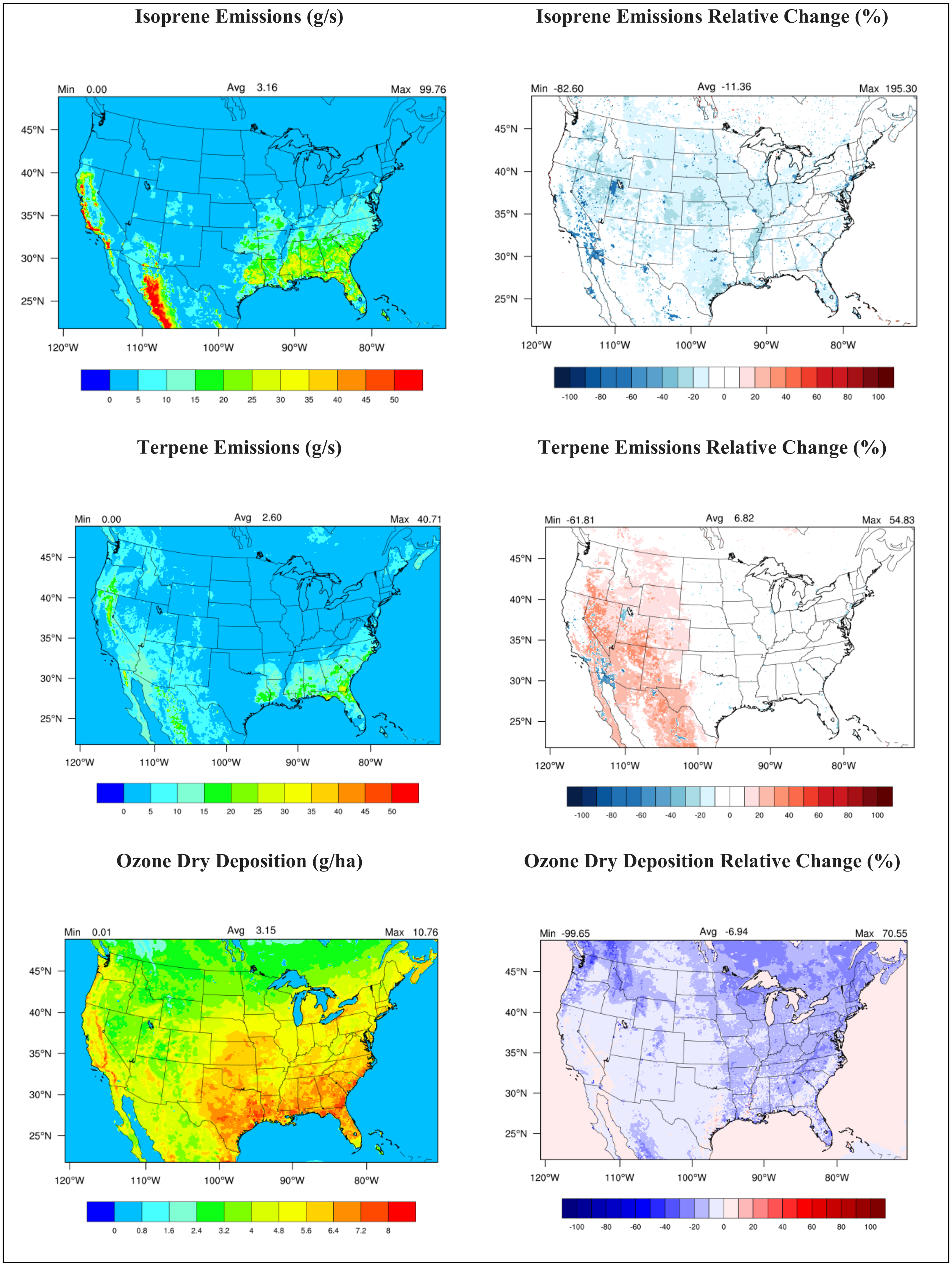
Isoprene Emissions (g/s)
Min 0.00
Avg 3.16
Max 99.76
45°N
40°N
35°N
30°N
25°N
120°W
110°W
100°W
90°W
80°W
0 5 10 15 20 25 30 35 40 45 50
Isoprene Emissions Relative Change (%)
Min -82.60
Avg -11.36
Max 195.30
-100 -80 -60 -40 -20 0 20 40 60 80 100
Terpene Emissions (g/s)
Min 0.00
Avg 2.60
Max 40.71
0 5 10 15 20 25 30 35 40 45 50
Terpene Emissions Relative Change (%)
Min -61.81
Avg 6.82
Max 54.83
-100 -80 -60 -40 -20 0 20 40 60 80 100
Ozone Dry Deposition (g/ha)
Min 0.01
Avg 3.15
Max 10.76
0 0.8 1.6 2.4 3.2 4 4.8 5.6 6.4 7.2 8
Ozone Dry Deposition Relative Change (%)
Min -99.65
Avg -6.94
Max 70.55
-100 -80 -60 -40 -20 0 20 40 60 80 100

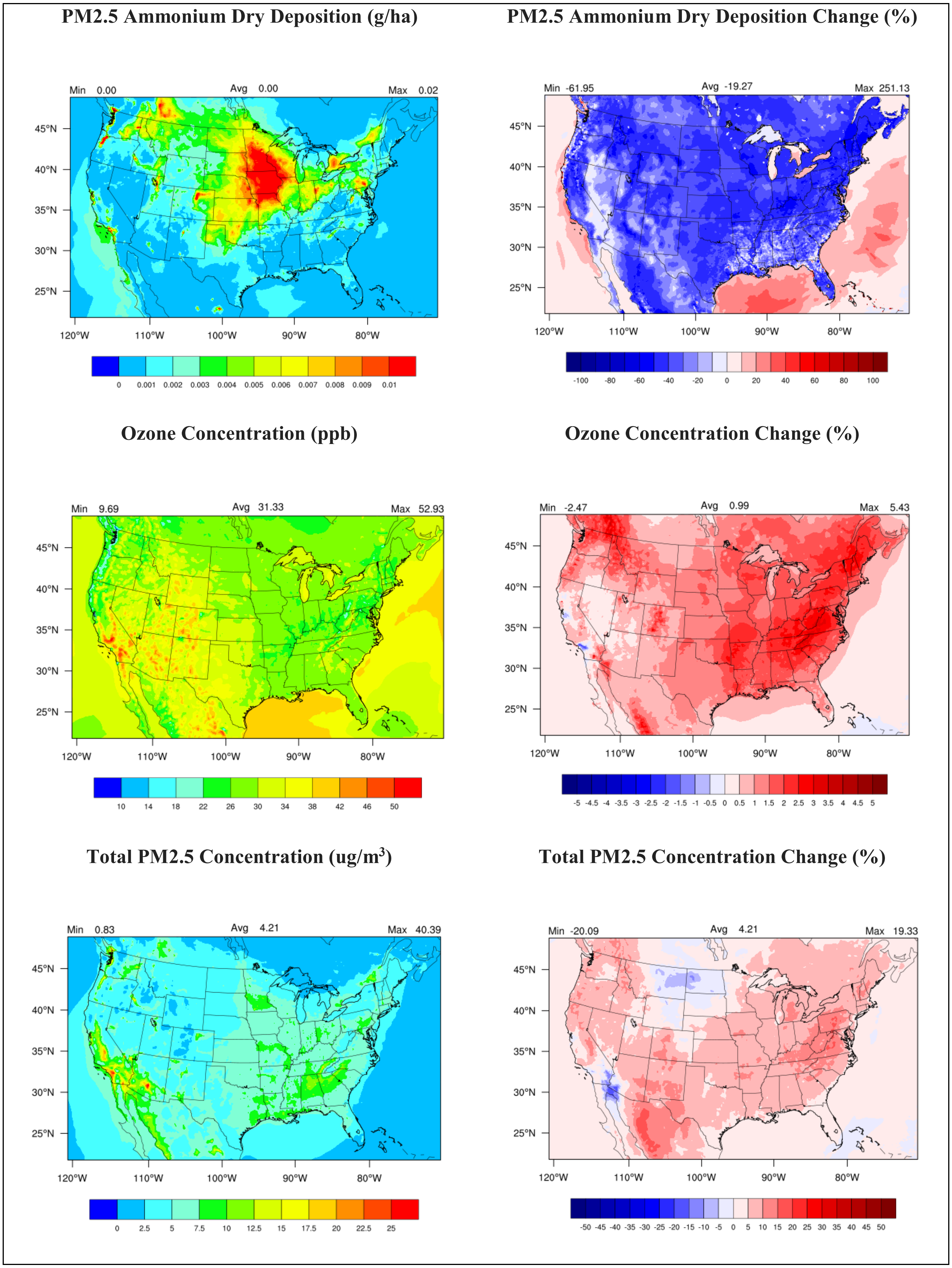

PM2.5 Ammonium Dry Deposition (g/ha)
Min 0.00
Avg 0.00
Max 0.02
45°N
40°N
35°N
30°N
25°N
120°W
110°W
100°W
90°W
80°W
0 0.001 0.002 0.003 0.004 0.005 0.006 0.007 0.008 0.009 0.01
PM2.5 Ammonium Dry Deposition Change (%)
Min -61.95
Avg -19.27
Max 251.13
-100 -80 -60 -40 -20 0 20 40 60 80 100
Ozone Concentration (ppb)
Min 9.69
Avg 31.33
Max 52.93
10 14 18 22 26 30 34 38 42 46 50
Ozone Concentration Change (%)
Min -2.47
Avg 0.99
Max 5.43
-5 -4.5 -4 -3.5 -3 -2.5 -2 -1.5 -1 -0.5 0 0.5 1 1.5 2 2.5 3 3.5 4 4.5 5
Total PM2.5 Concentration (ug/m³)
Min 0.83
Avg 4.21
Max 40.39
0 2.5 5 7.5 10 12.5 15 17.5 20 22.5 25
Total PM2.5 Concentration Change (%)
Min -20.09
Avg 4.21
Max 19.33
-50 -45 -40 -35 -30 -25 -20 -15 -10 -5 0 5 10 15 20 25 30 35 40 45 50

Figure S3. Spatial comparison plots of average October 2020 NACC-CMAQ simulation tests with an LAI=4 (left) and relative difference plots (right) with inclusion of VIIRS rapid-refresh (8-day product) of LAI and GVF for isoprene emissions (row 1), terpene emissions (row 2), ozone dry deposition (row 3), fine particulate ($PM_{2.5}$) ammonium dry deposition (row 4), ozone concentration (row 5), and total $PM_{2.5}$ concentration.

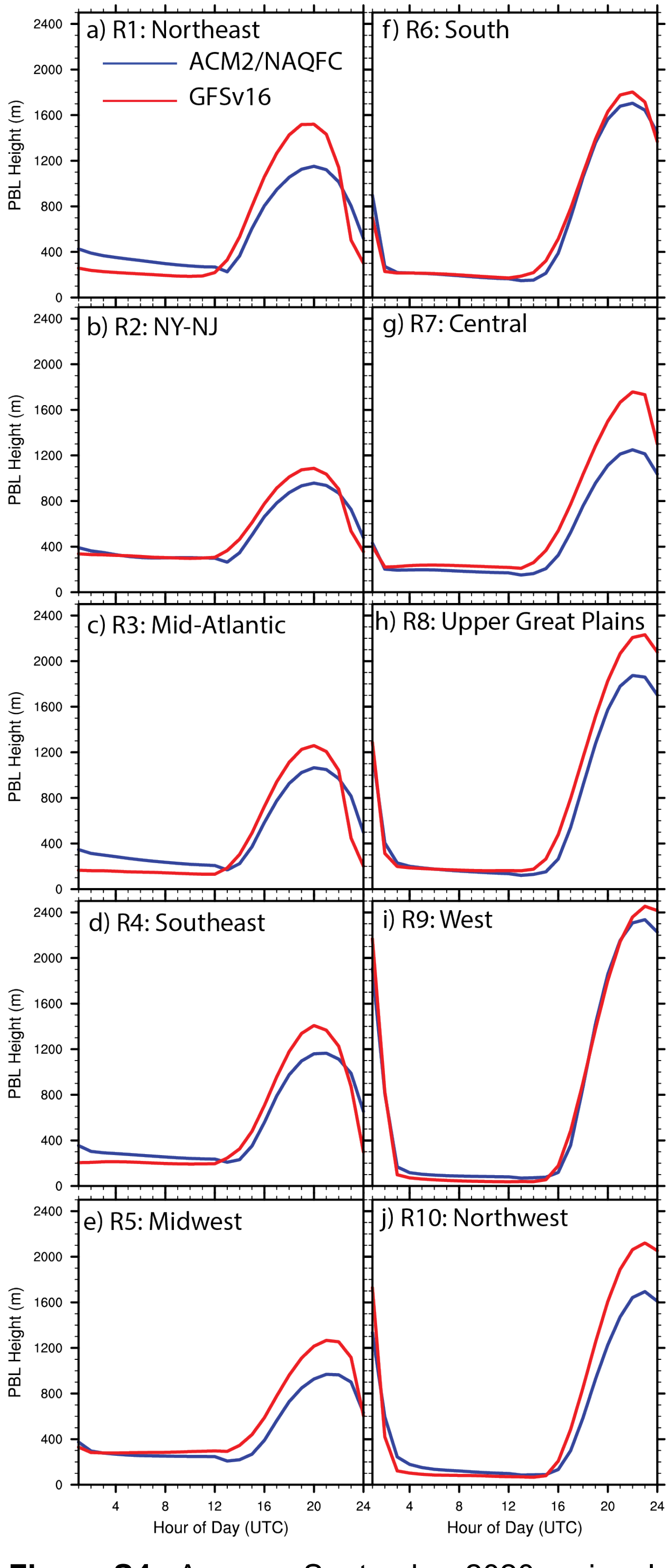

Figure S4. Average September 2020 regional diurnal patterns of PBLH (m) for ACM2/NAQFC (blue) and GFSv16 (red) over the CONUS. The regions are based on https://www.epa.gov/aboutepa/regional-and-geographic-offices.

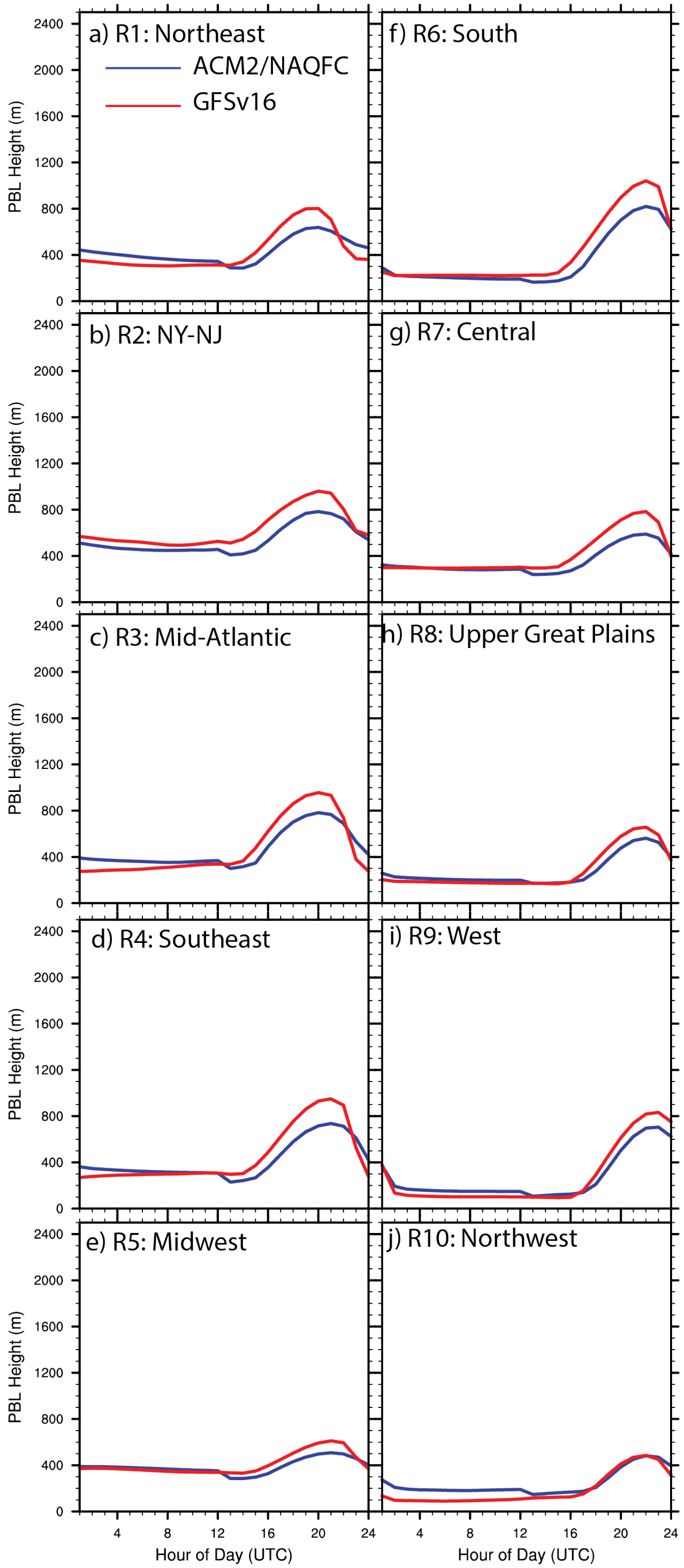

Figure S5. Same as Figure S4, but for January 2021.

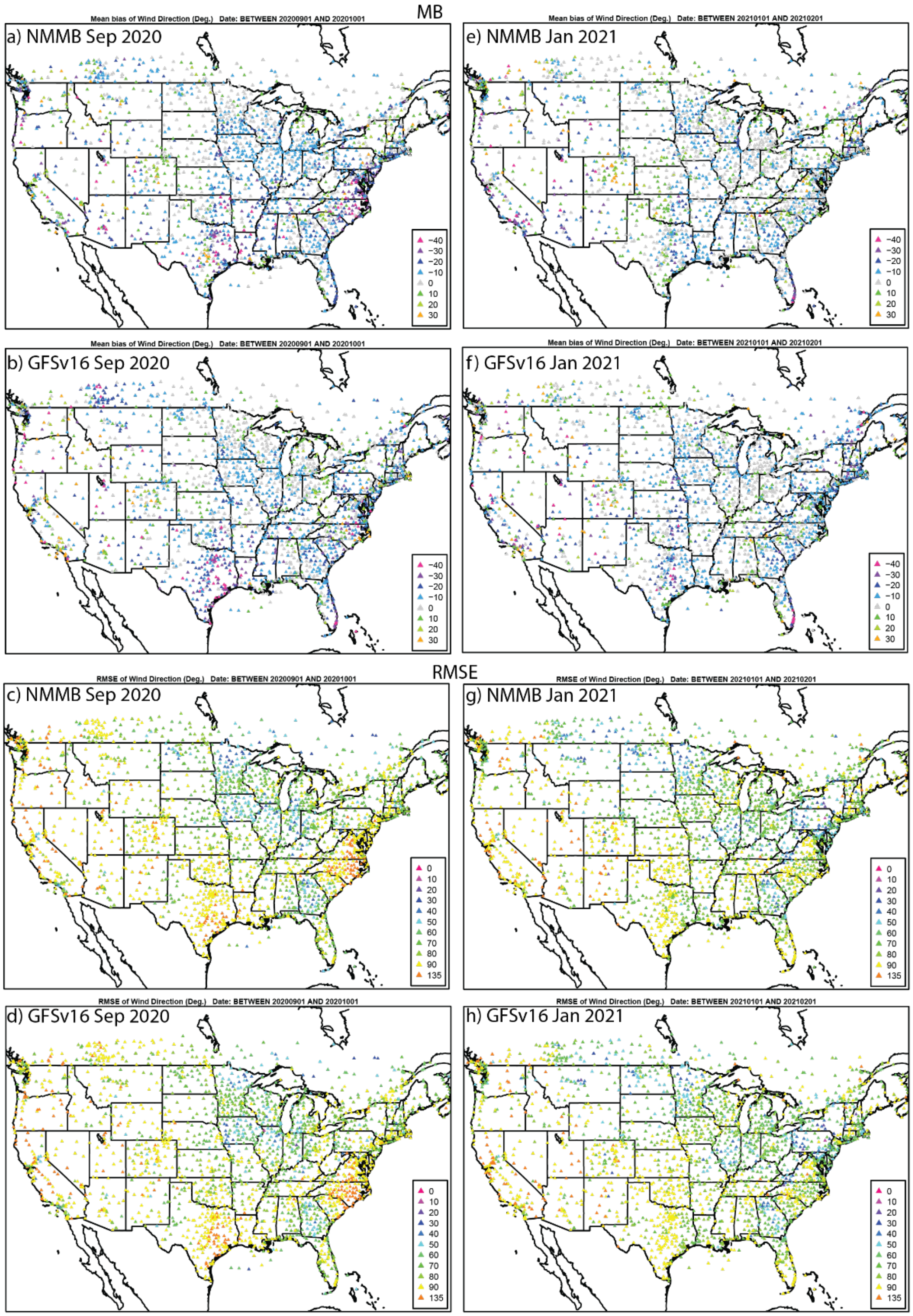

Figure S6. Average day 1 (0-24 hr) forecasted WDIR10 MB (degrees) and RMSE (degrees) for NMMB and GFSv16 during a)-d) September 2020 and e)-h) January 2021 compared to METAR-MADIS observations.

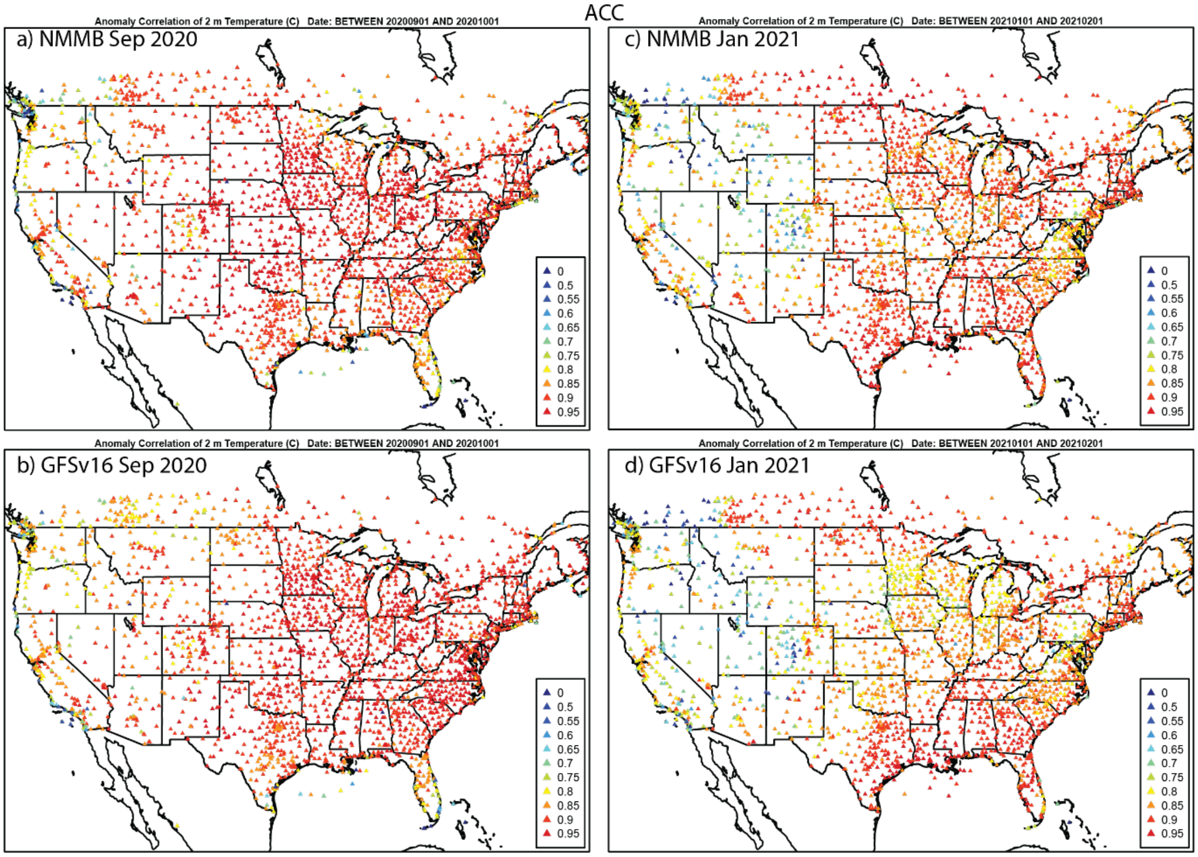

Figure S7. Average day 1 (0-24 hr) forecasted TEMP2 Anomaly Correlation Coefficient (ACC) for NMMB and GFSv16 during a)-d) September 2020 and e)-h) January 2021 compared to METAR-MADIS observations.

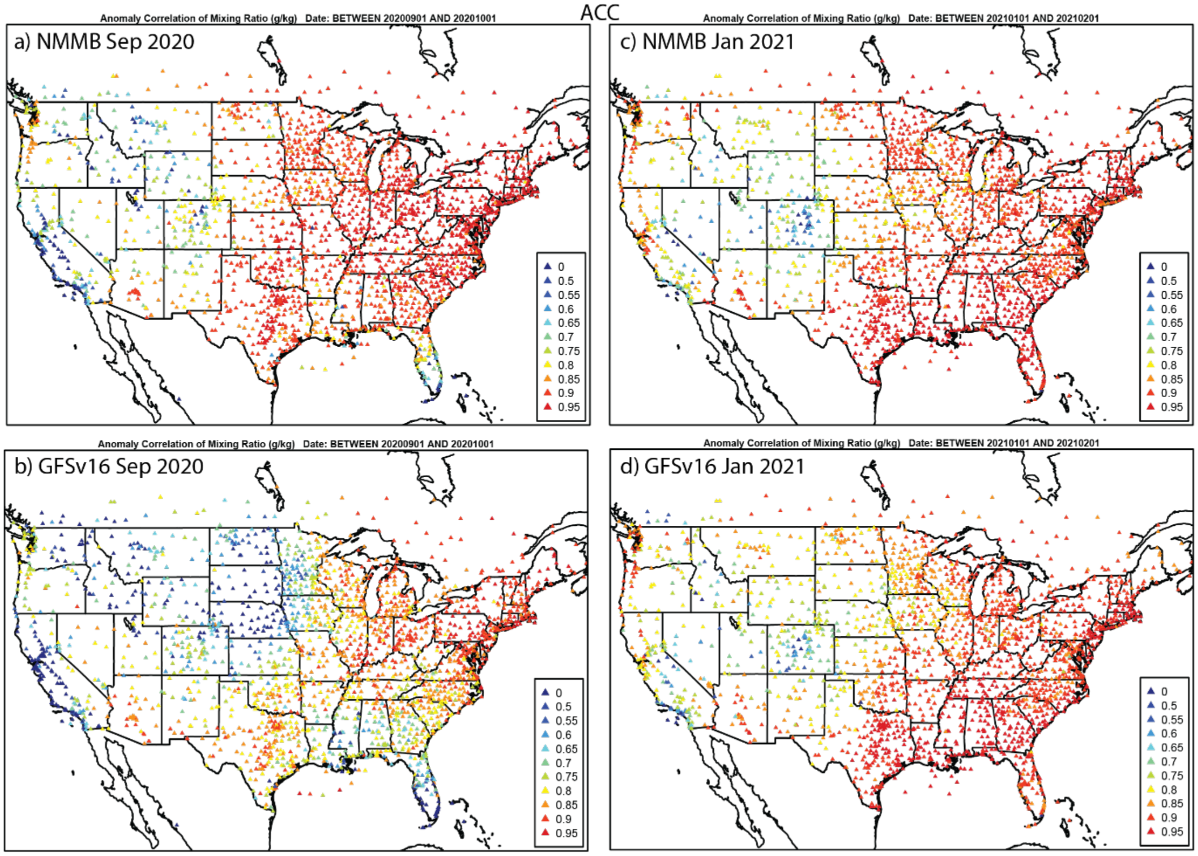

Figure S8. Same as in Figure S7, but for Q2.

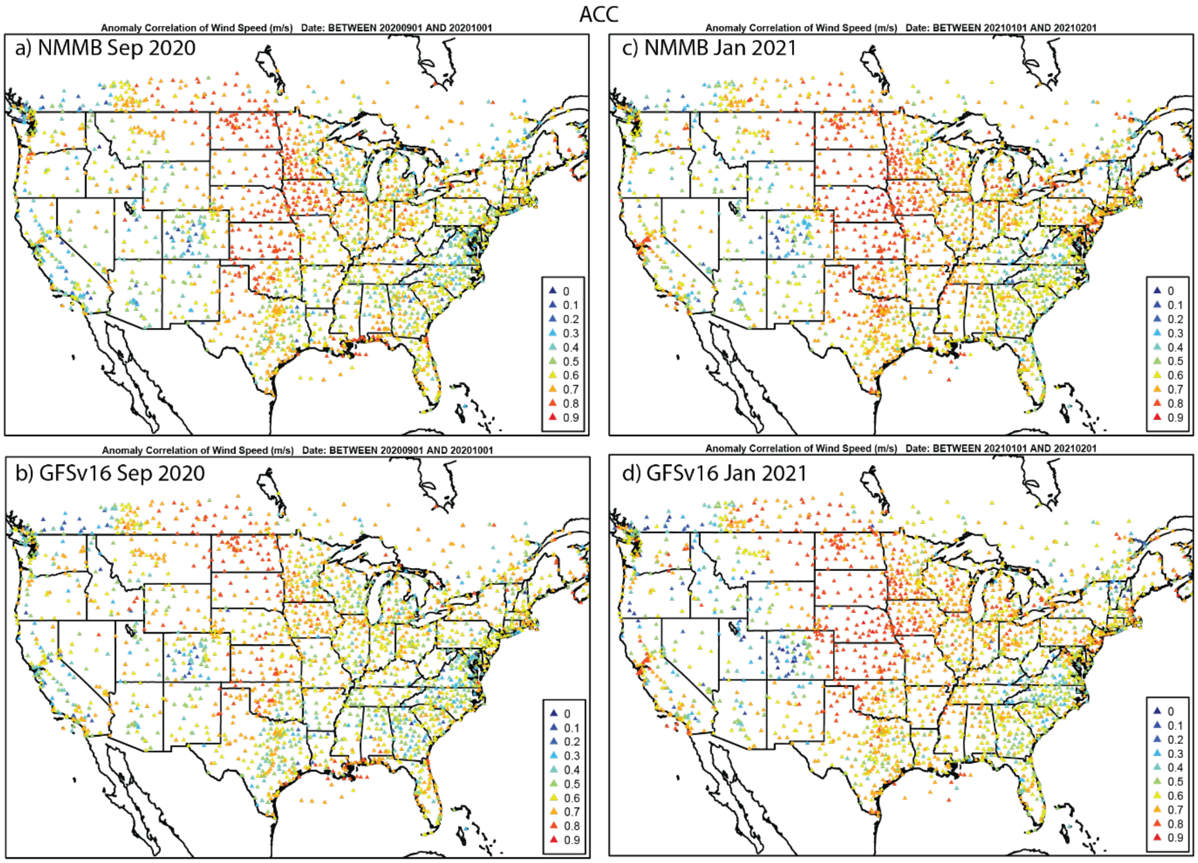

Figure S9. Same as in Figure S7, but for WSPD10.

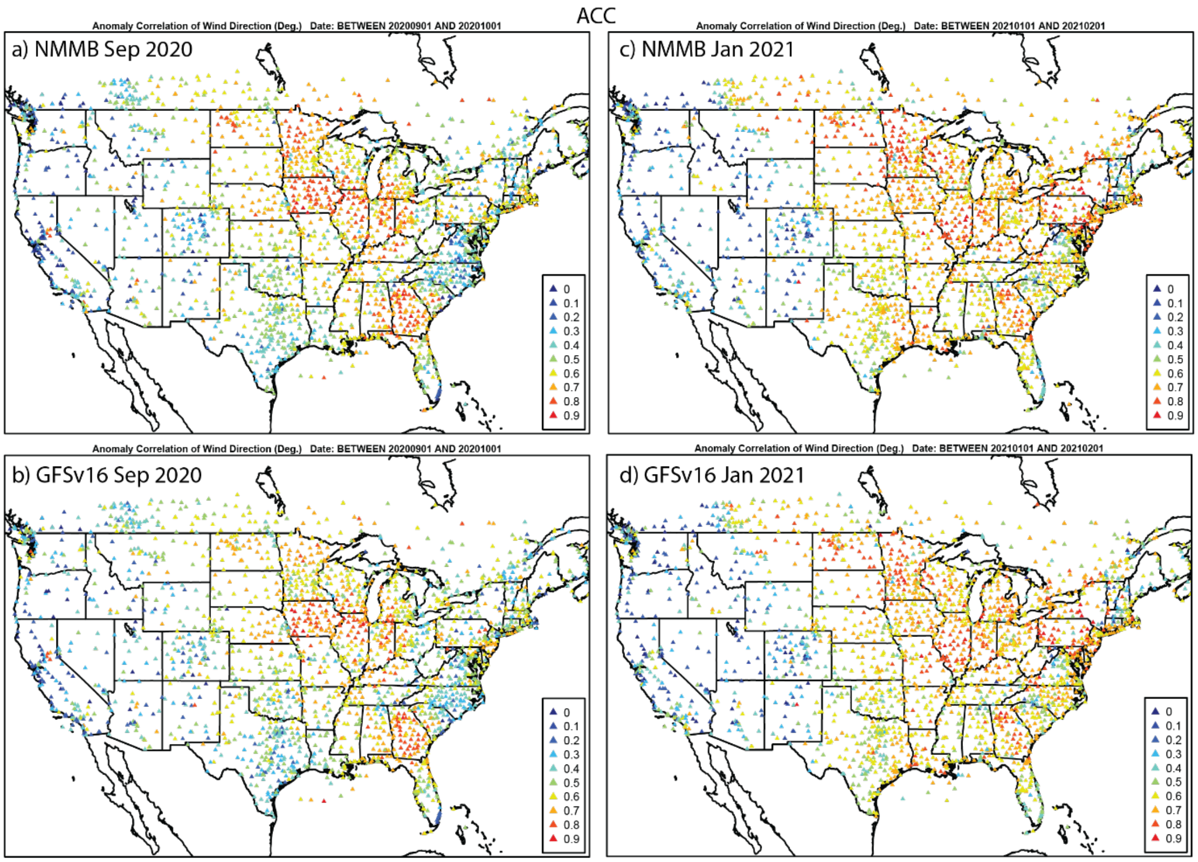

Figure S10. Same as in Figure S7, but for WDIR10.

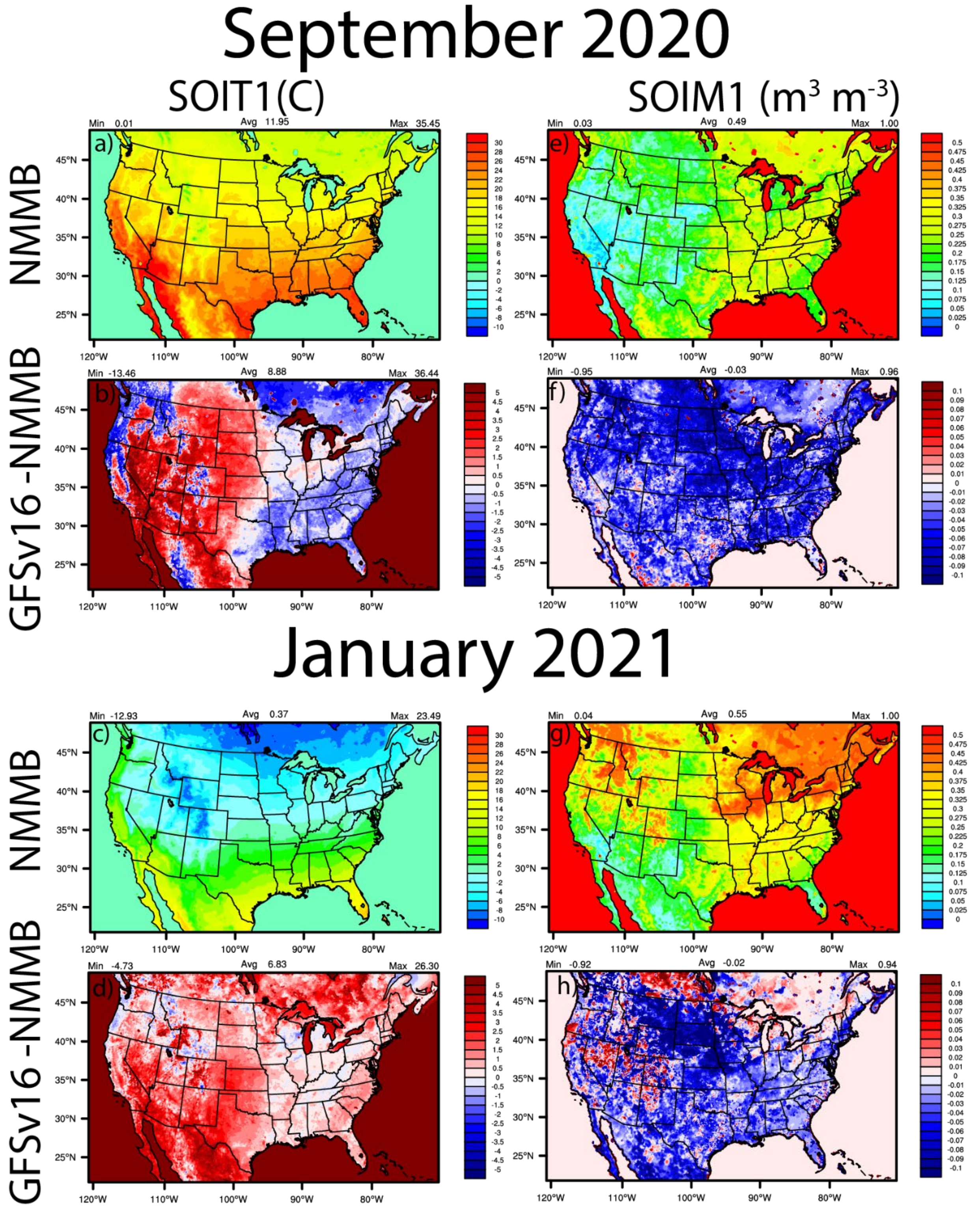

Figure S11. September 2020 and January 2021 spatial average plots for NMMB, and the absolute differences for GFSv16 - NMMB for the top layer soil temperature (SOIT1) and soil moisture (SOIM1).

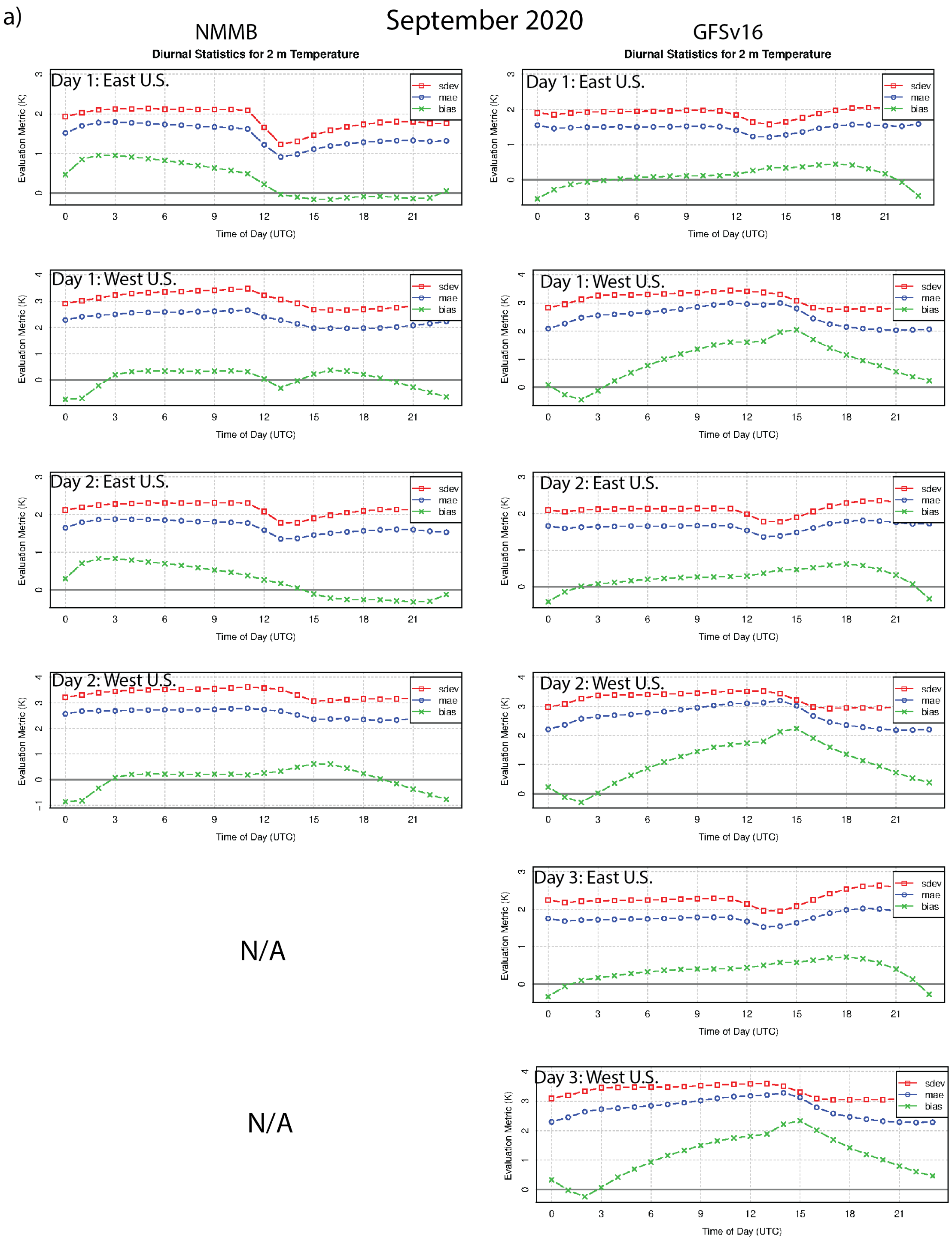

Figure S12a. September 2020 average diurnal TEMP2 statistics (red = standard deviation; blue = mean absolute error; green = bias) for day 1, 2, and 3 (GFSv16) forecasts in the east (< 100° W) and west CONUS (> 100° W).

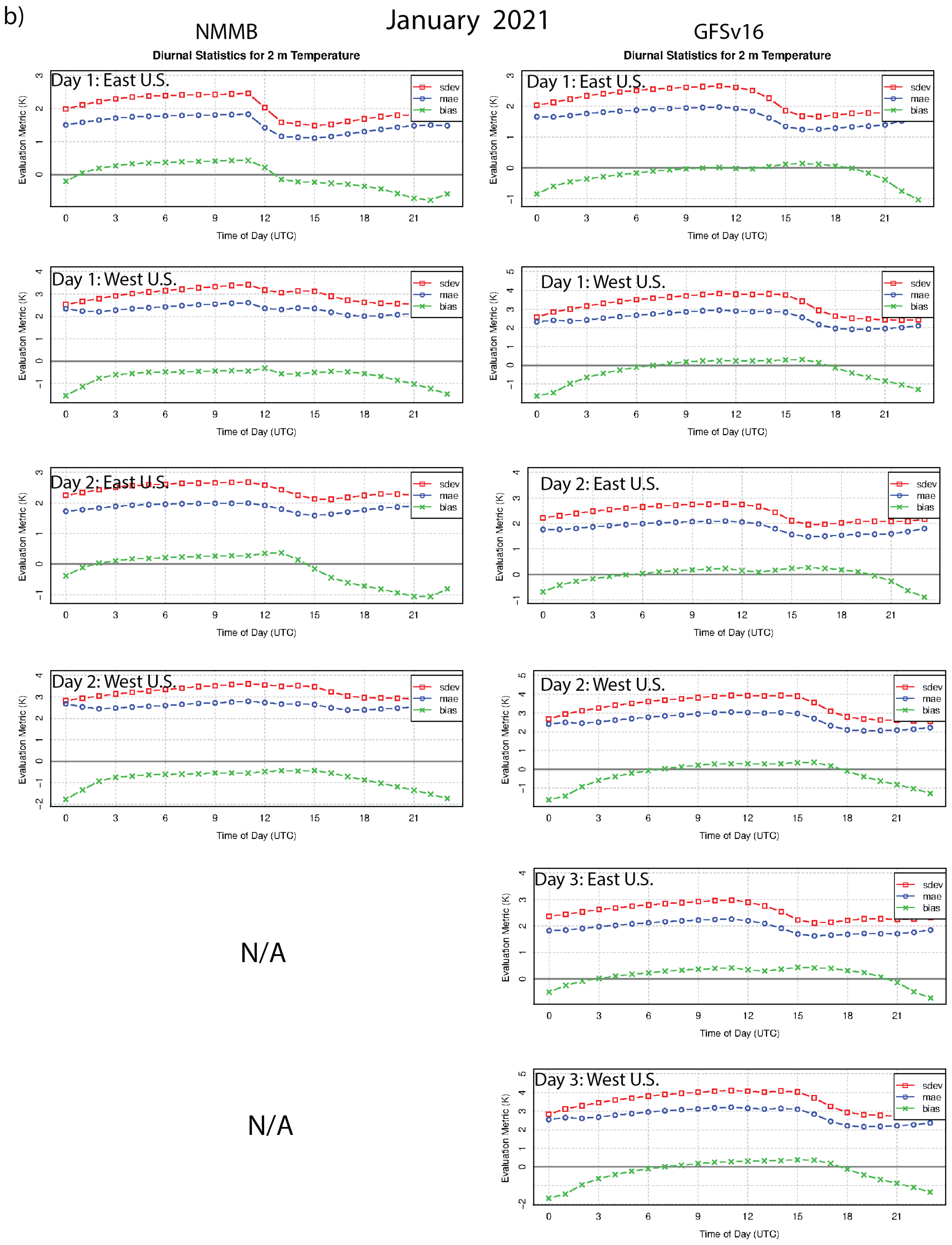

Figure S12b. Same as in Figure S12a, but for January 2021.

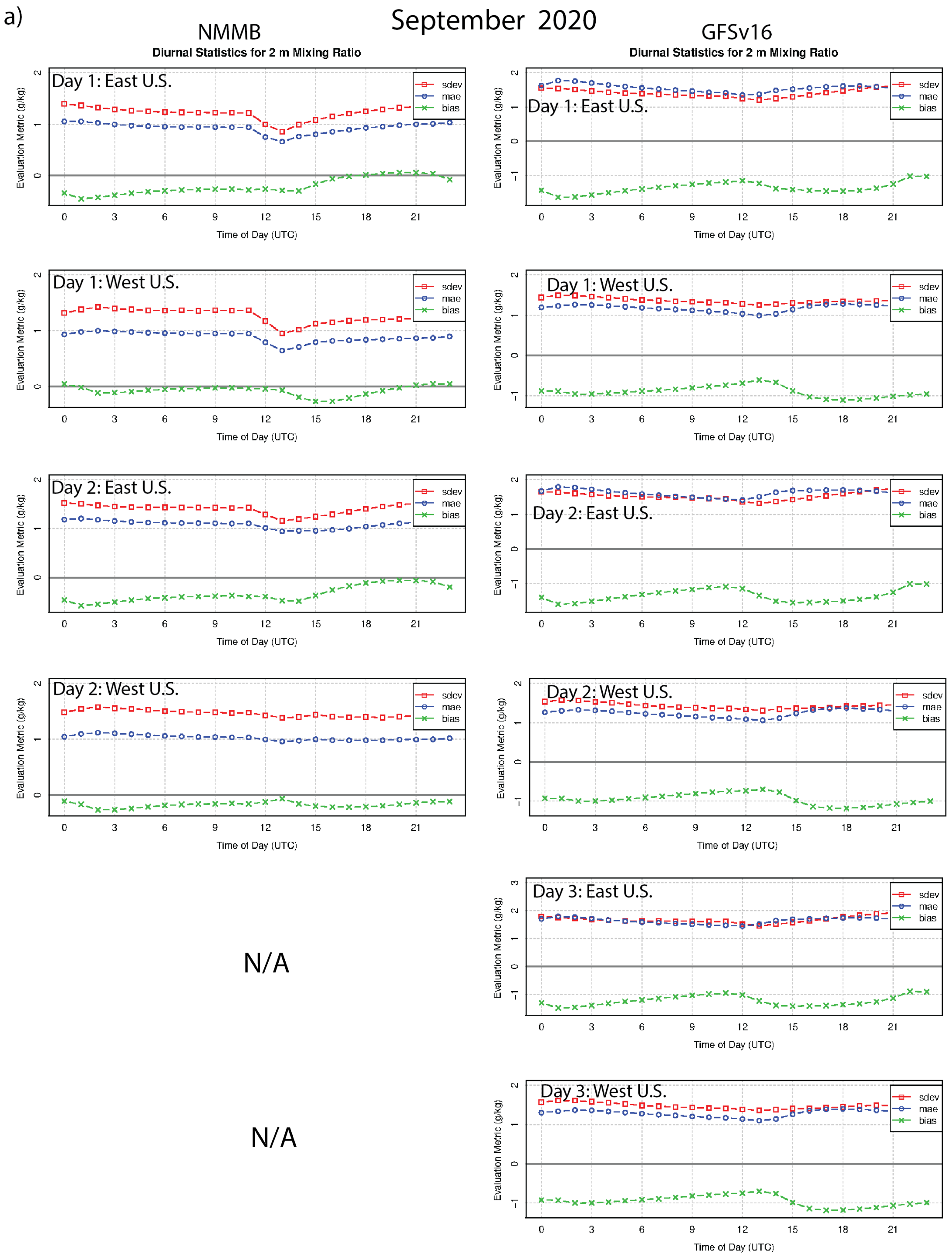

Figure S13a. September 2020 average diurnal Q2 statistics (red = standard deviation; blue = mean absolute error; green = bias) for day 1, 2, and 3 (GFSv16) forecasts in the east (< 100° W) and west CONUS (> 100° W).

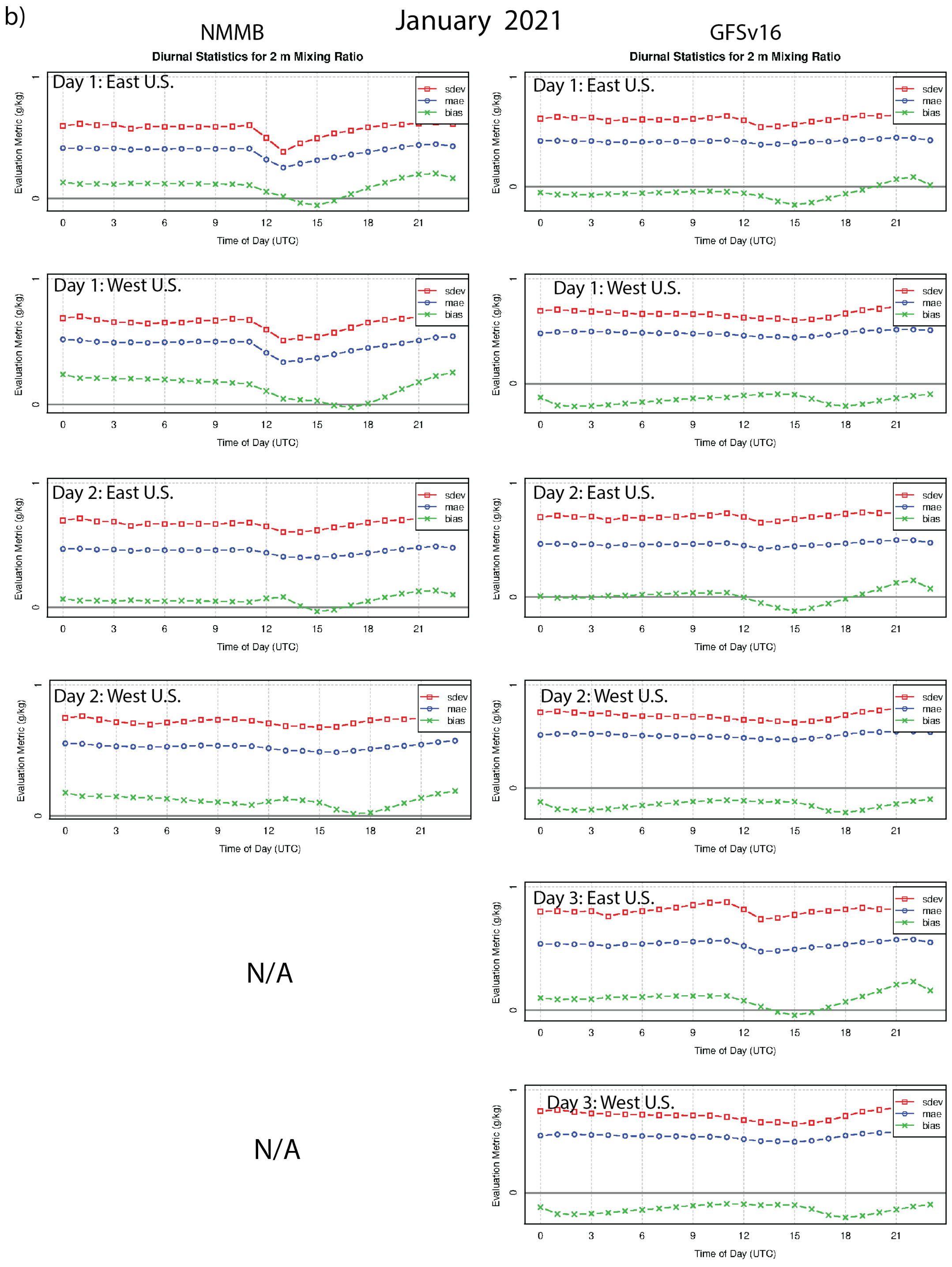

Figure S13b. Same as in Figure S13a, but for January 2021.

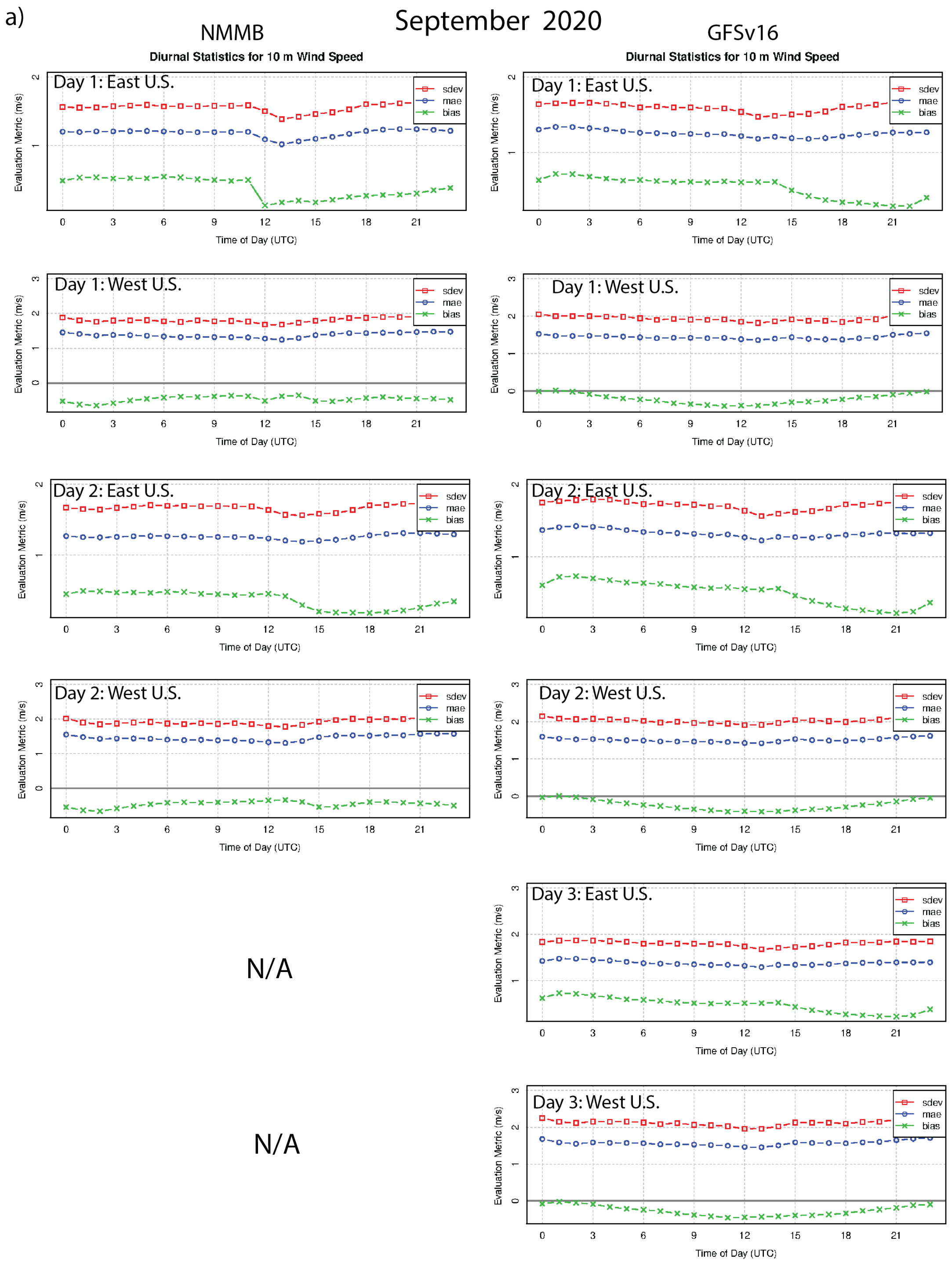

Figure S14a. September 2020 average diurnal WSPD10 statistics (red = standard deviation; blue = mean absolute error; green = bias) for day 1, 2, and 3 (GFSv16) forecasts in the east (< 100° W) and west CONUS (> 100° W).

b)

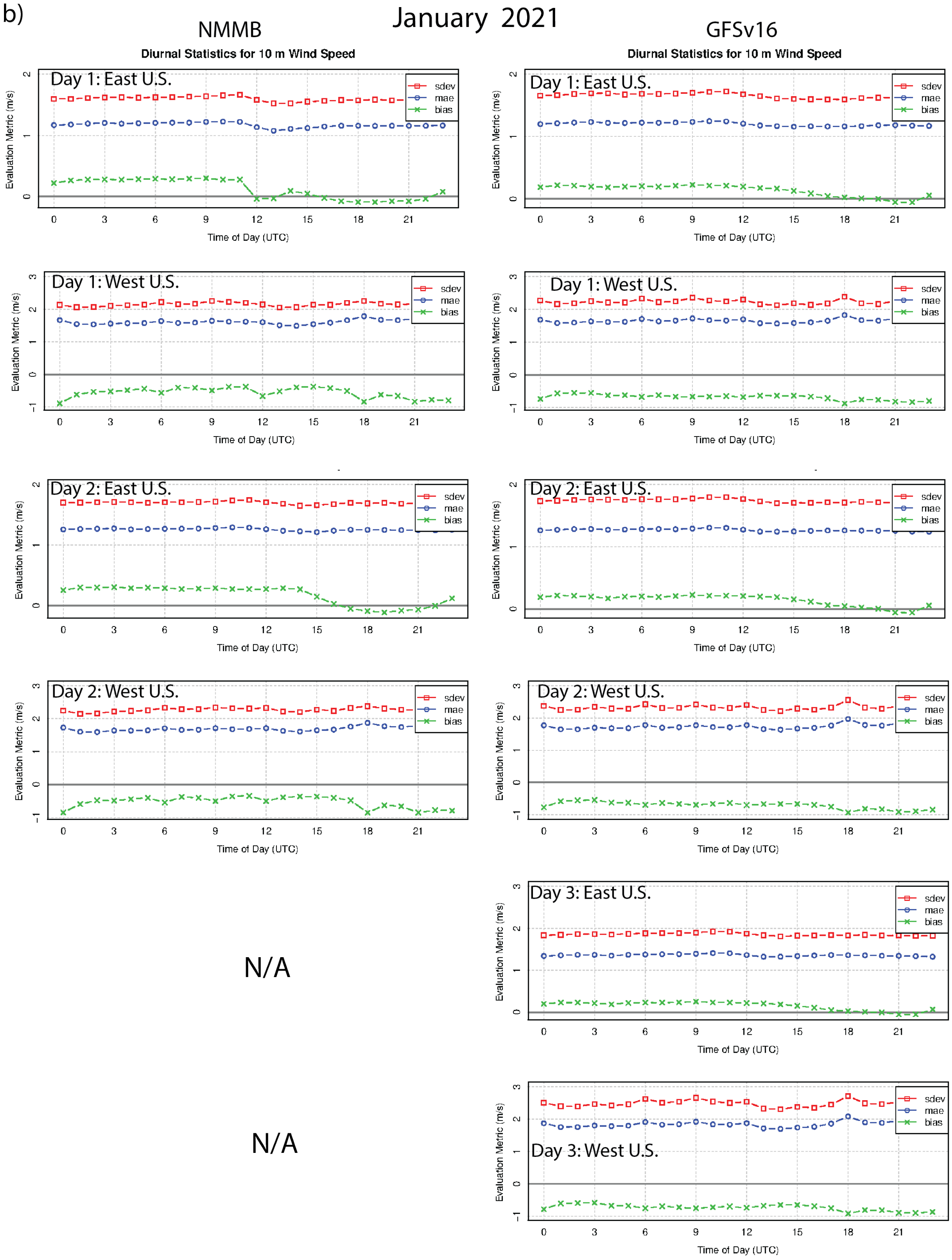

Figure S14b. Same as in Figure S14a, but for January 2021.

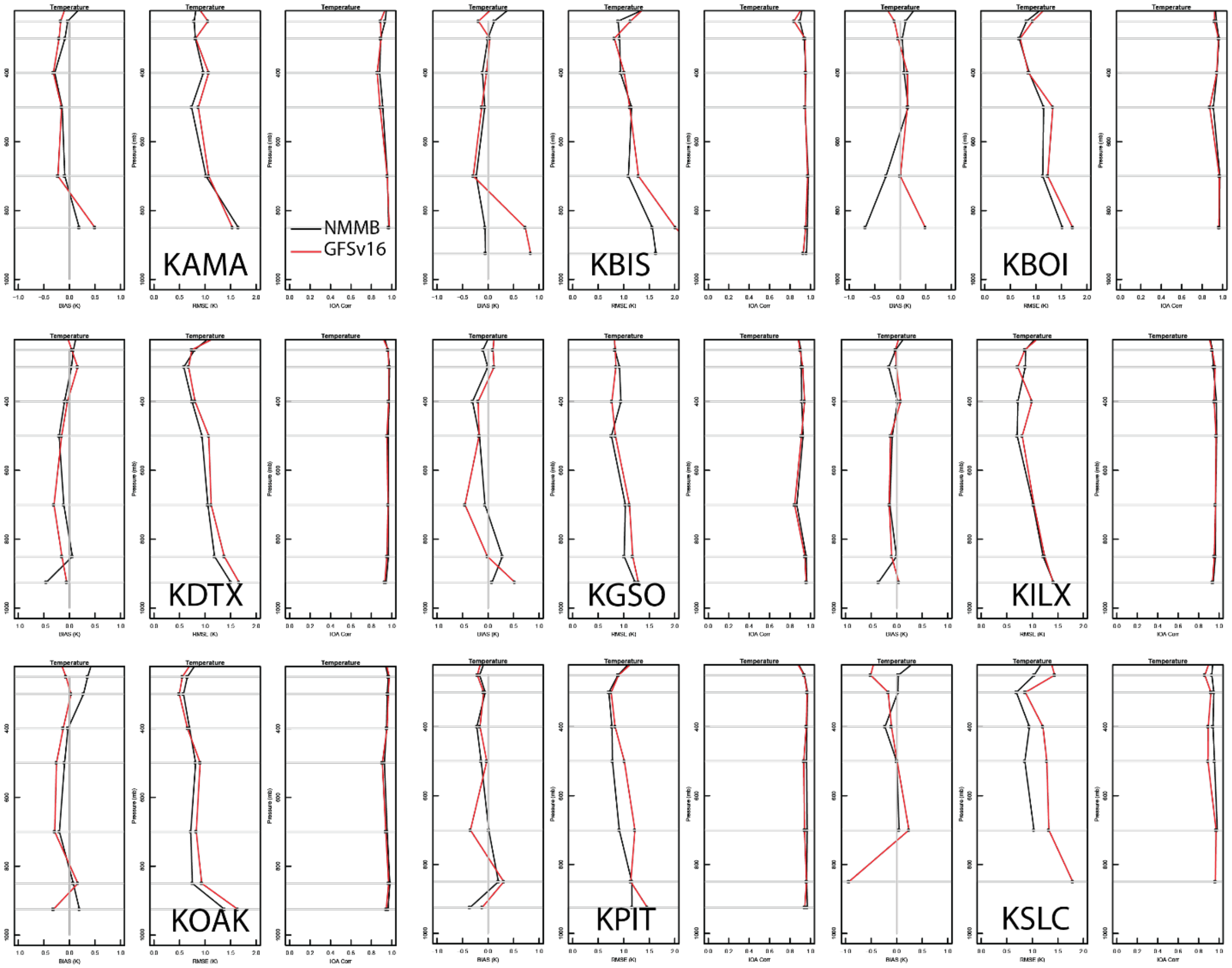

Figure S15a. September 2020 vertical (1000 – 250 mb) temperature (TEMP) statistics (MB, RMSE, and IOA) for NMMB (black) and GFSv16 (red) against the following select RAOB sites in CONUS: South (KAMA; Amarillo, TX), west-north central (KBIS; Bismark, ND), northwest (KBOI; Boise, ID), Midwest (KDTX; Detroit, MI), southeast (KGSO; Greensboro, NC), central (KILX), west (KOAK; Oakland, CA), northeast (KPIT; Pittsburgh, PA), and southwest (KSLC; Salt Lake City, UT). The relative locations of the RAOB sites are shown in Supporting Figures S18-S19.

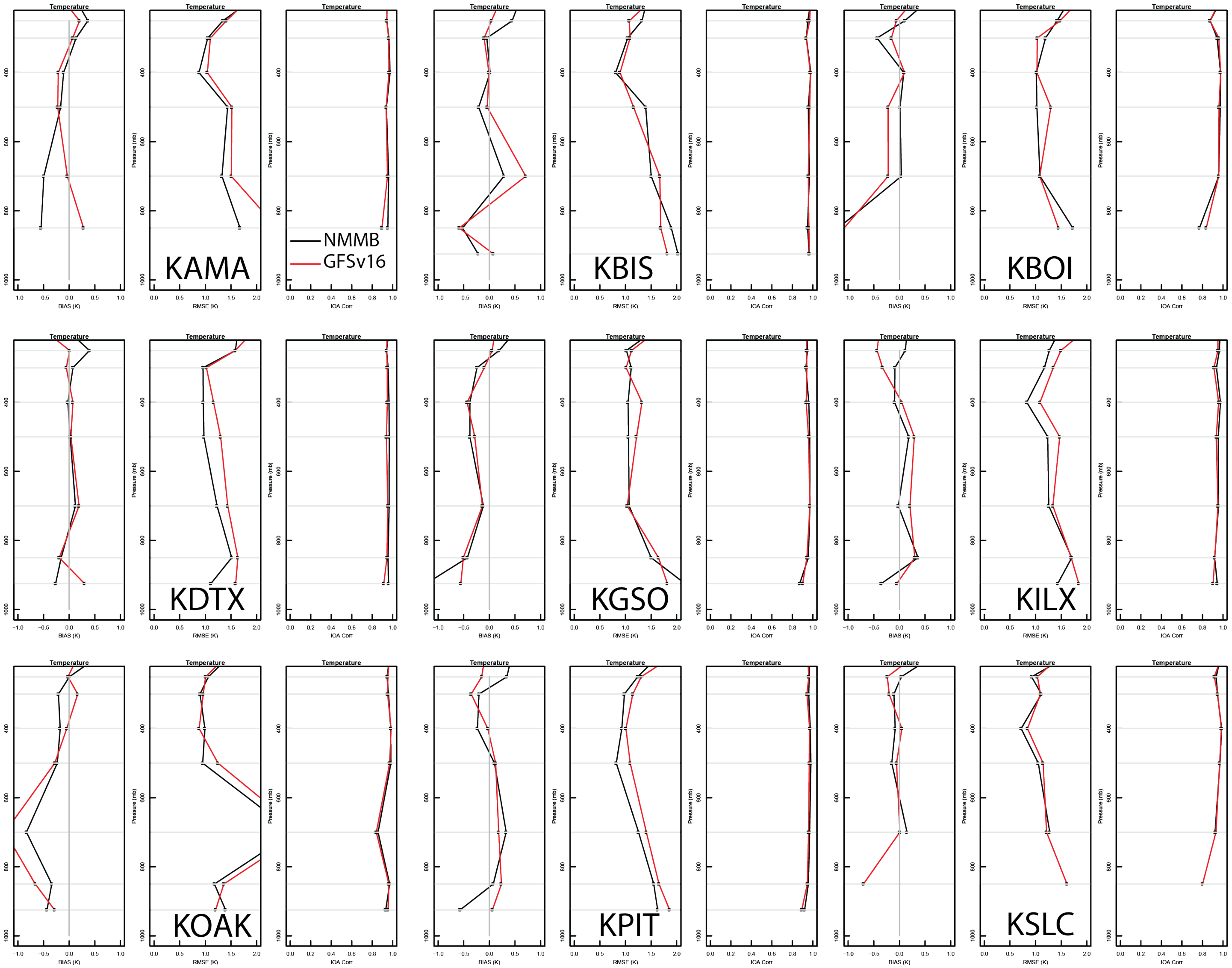

Figure S15b. Same as in Figure S15a, but for January 2021.

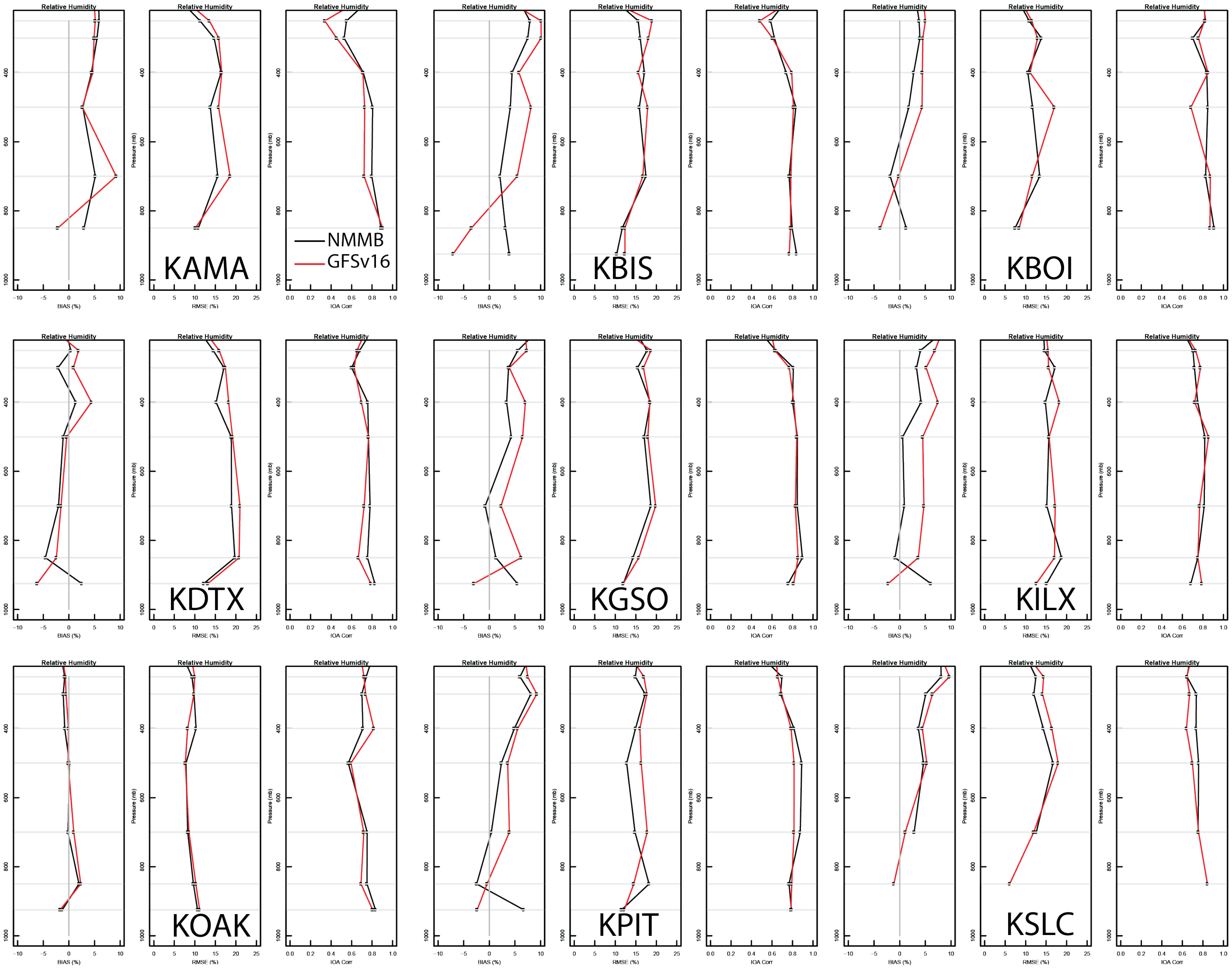

Figure S16a. September 2020 vertical (1000 – 250 mb) relative humidity (RH) statistics (MB, RMSE, and IOA) for NMMB (black) and GFSv16 (red) against select RAOB sites in CONUS. The RAOB sites are listed in Supporting Figure S15a, and their relative locations are shown in Supporting Figures S18-S19.

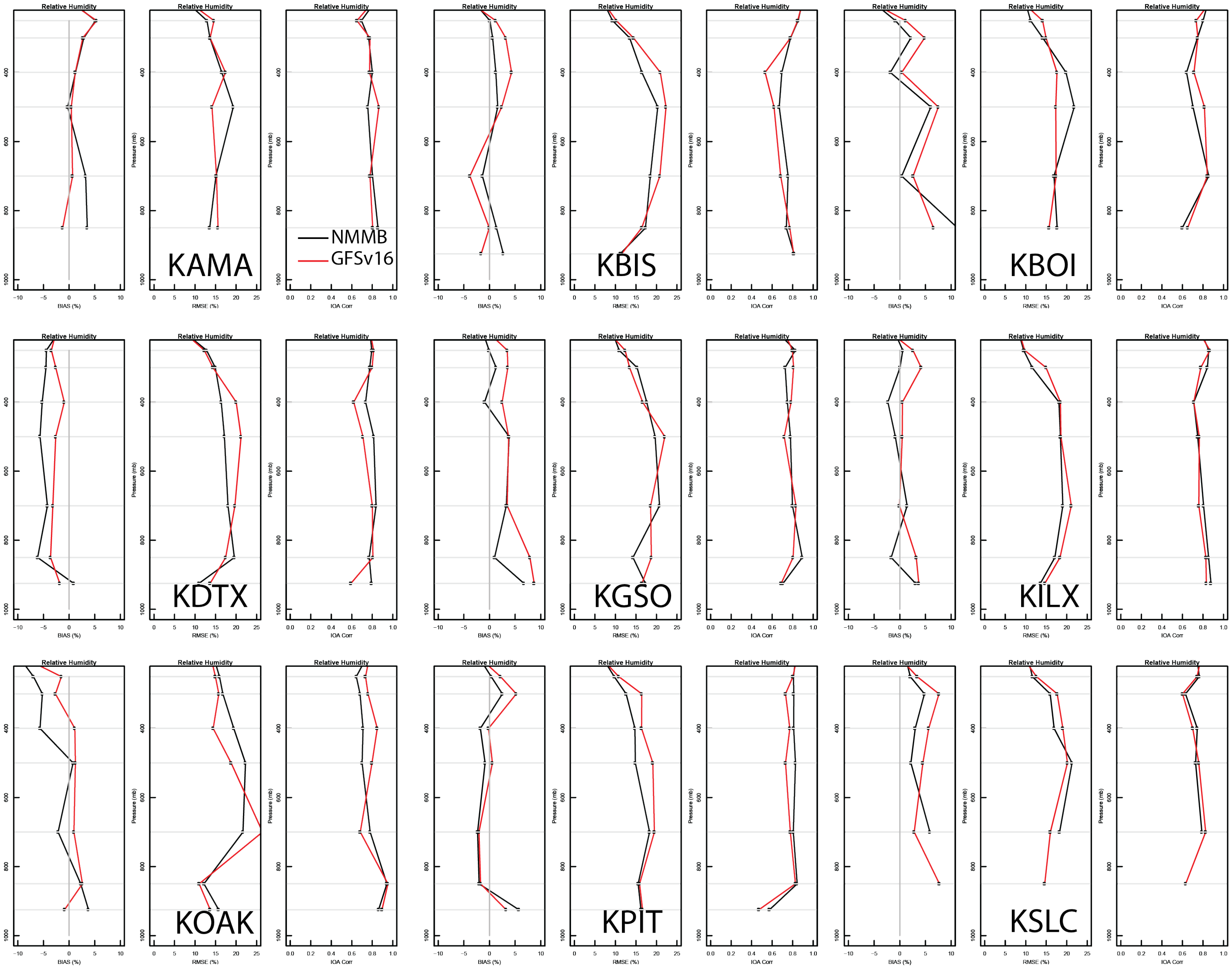

Figure S16b. Same as in Figure S16a, but for January 2021.

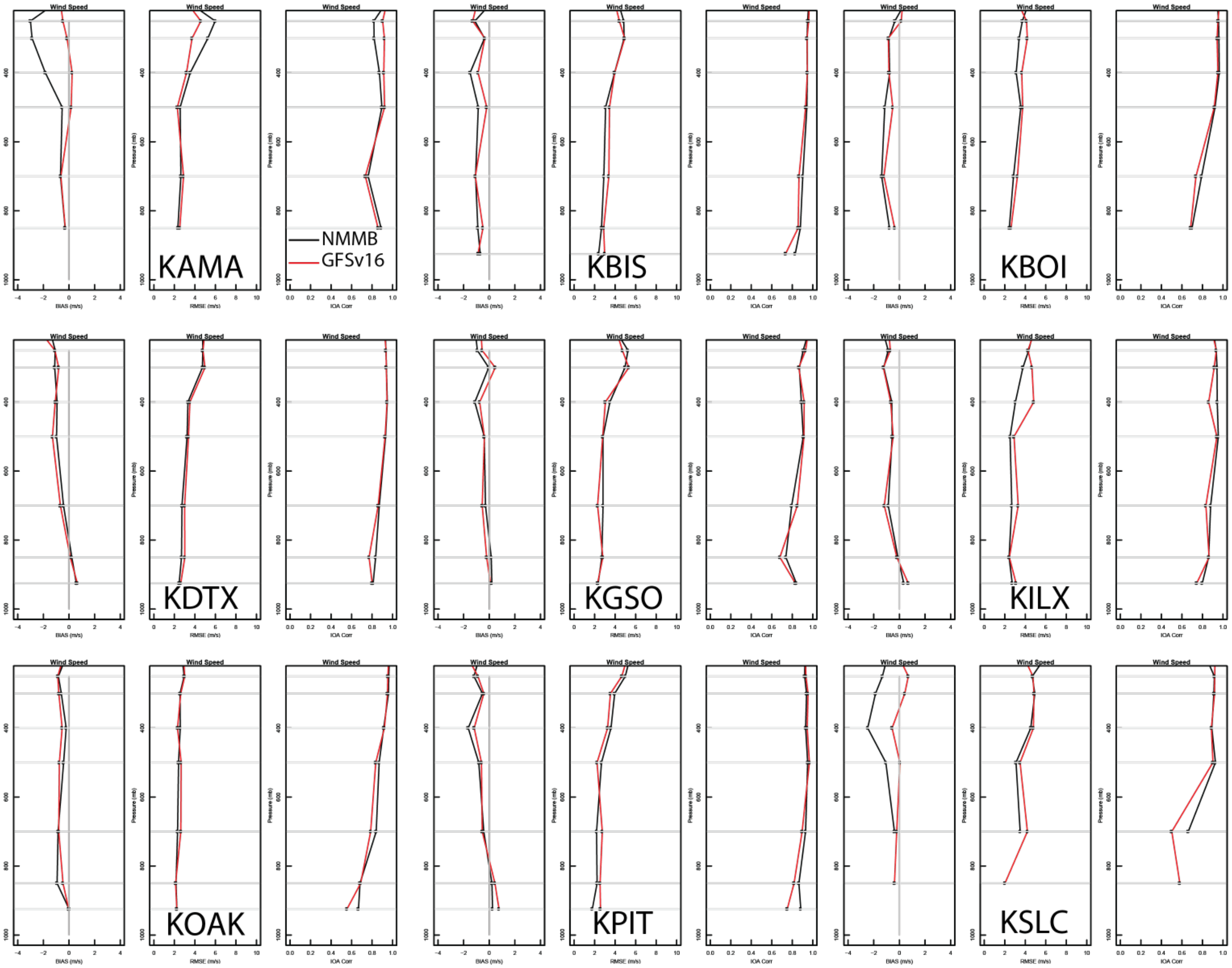

Figure S17a. September 2020 vertical (1000 – 250 mb) wind speed (WSPD) statistics (MB, RMSE, and IOA) for NMMB (black) and GFSv16 (red) against select RAOB sites in CONUS. Sites are listed in Supporting Figure S15a, and their relative locations are shown in Supporting Figures S18-S19.

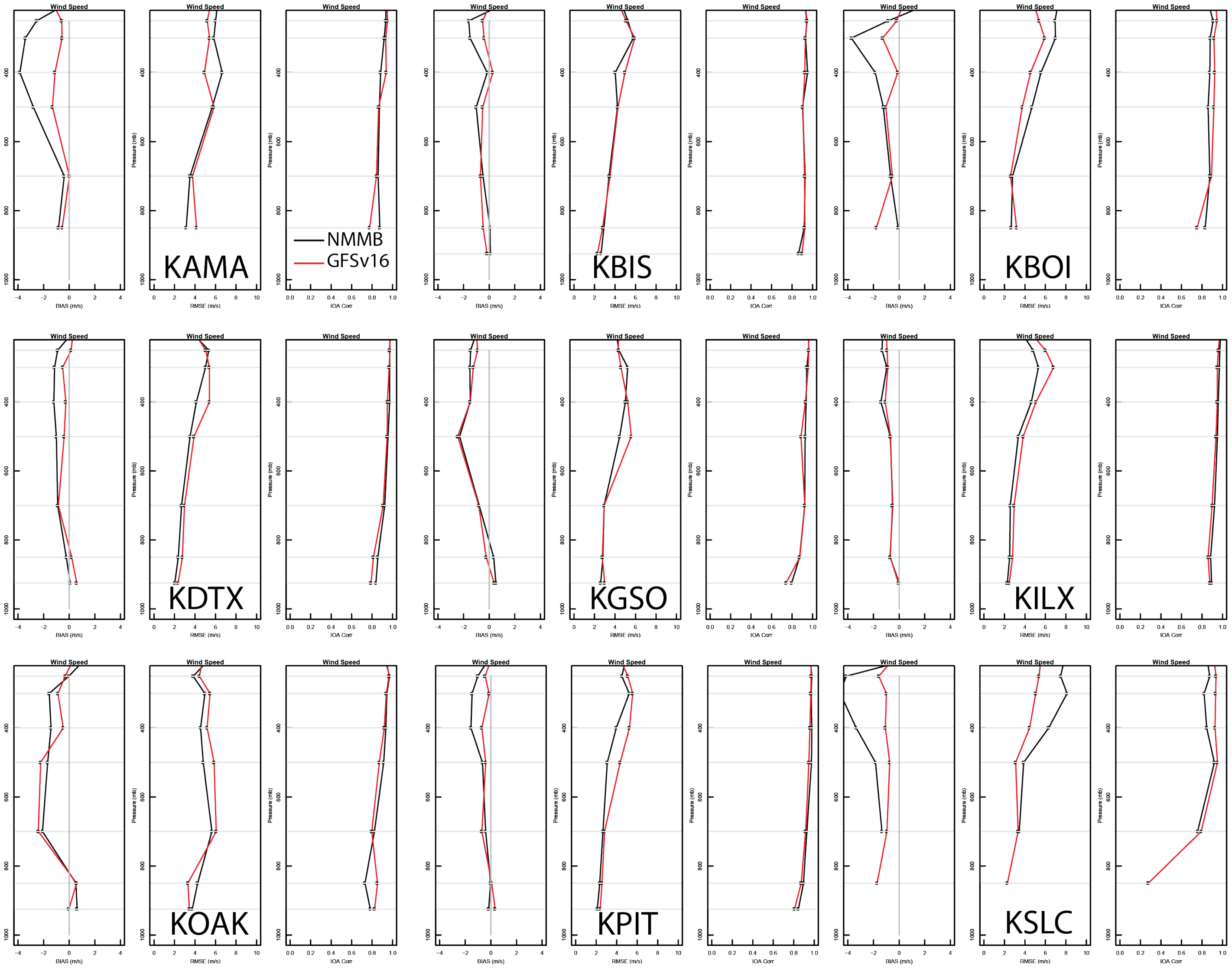

Figure S17b. Same as in Figure S17a, but for January 2021.

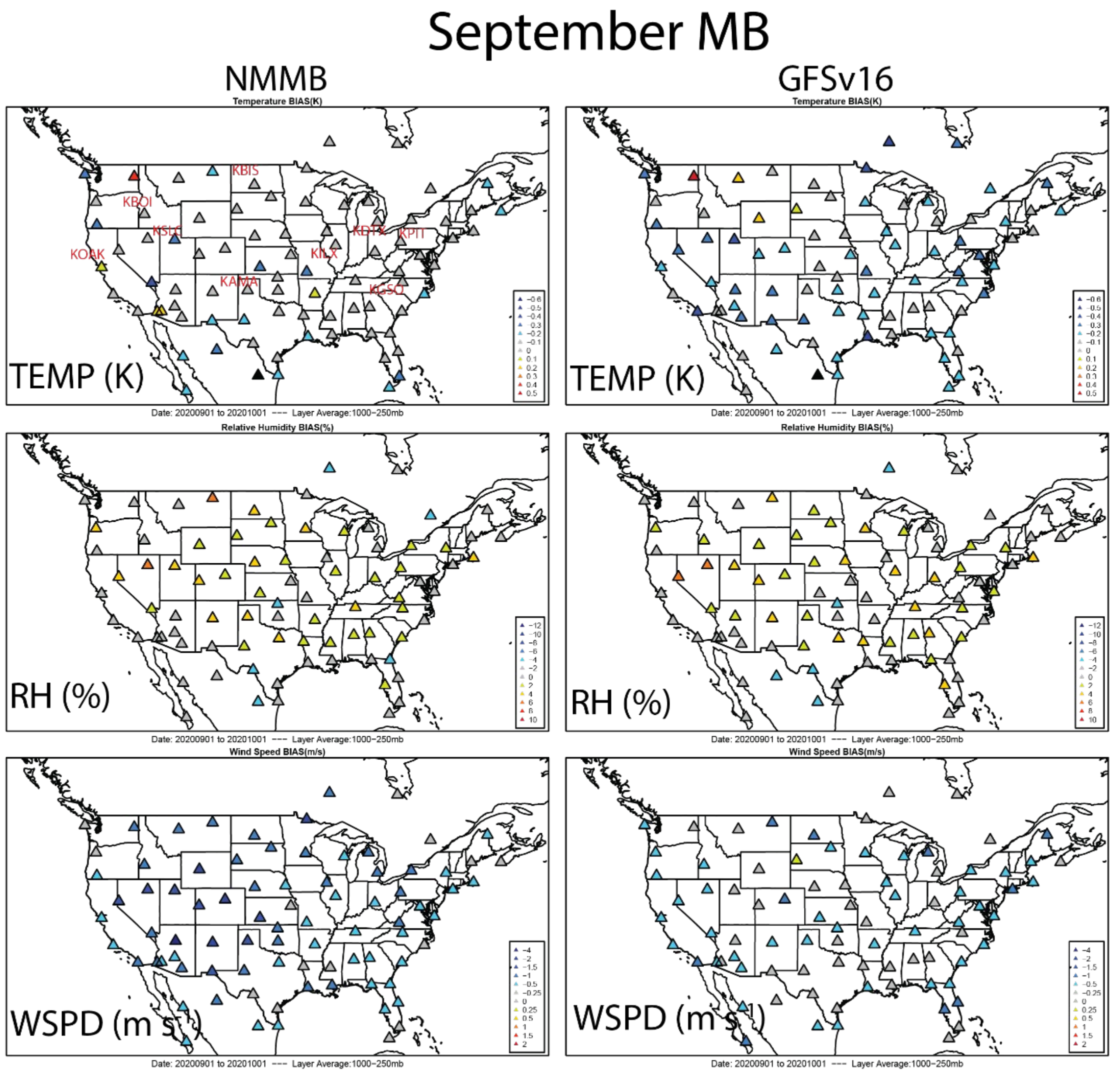

Figure S18a. Average September 2020 spatial mean bias (MB) comparison of the 1000-250 layer average NMMB (left) and GFSv16 (right) modeled temperature (TEMP; top), relative humidity (RH; middle), and wind speed (WSPD; bottom) compared against RAOB sites in CONUS. RAOB sites labeled in the top left panel pertain to the profile comparisons in the main text and Supporting Figures S15-S17.

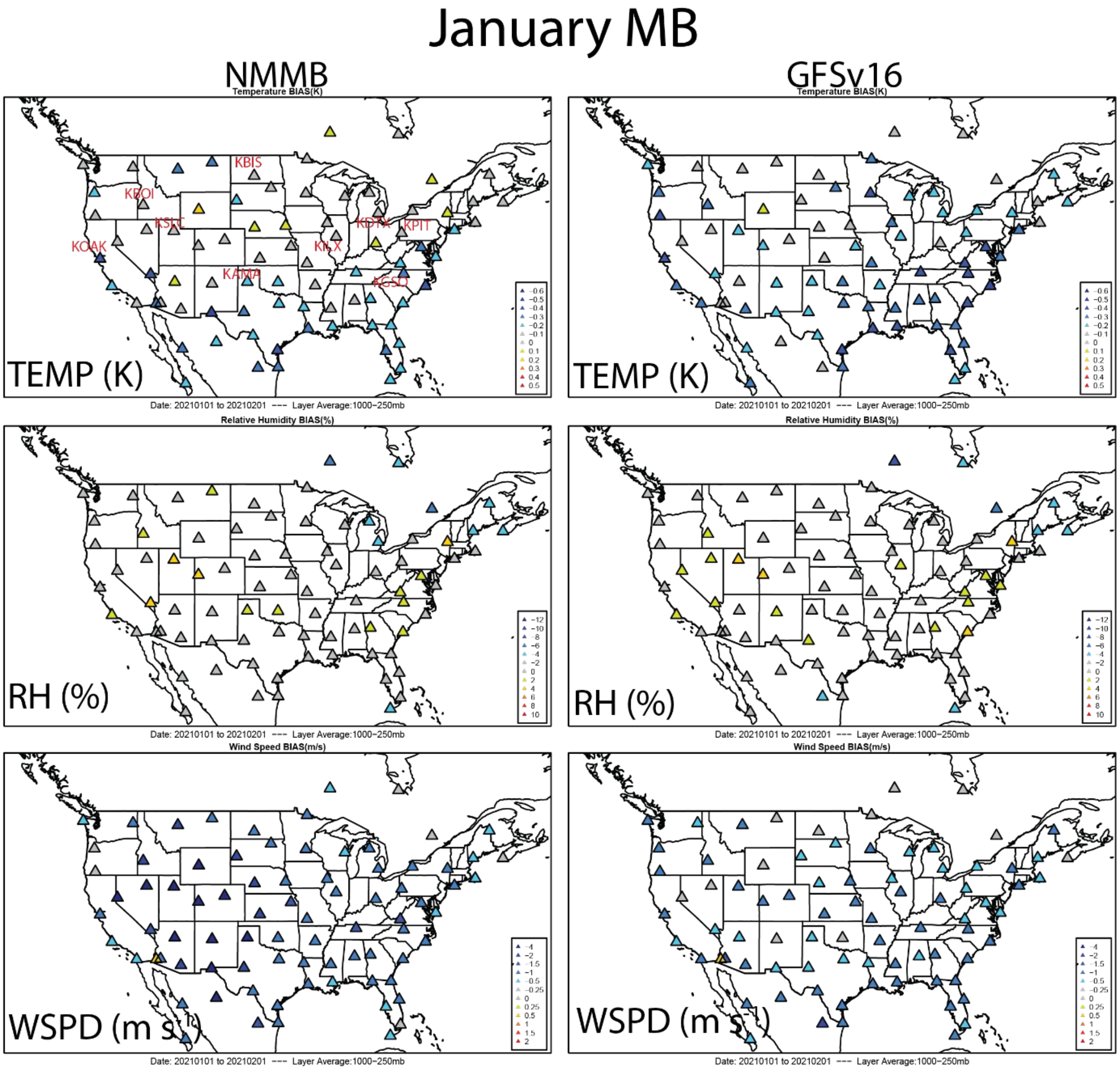

Figure S18b. Same as in Figure S18a, but for January 2021.

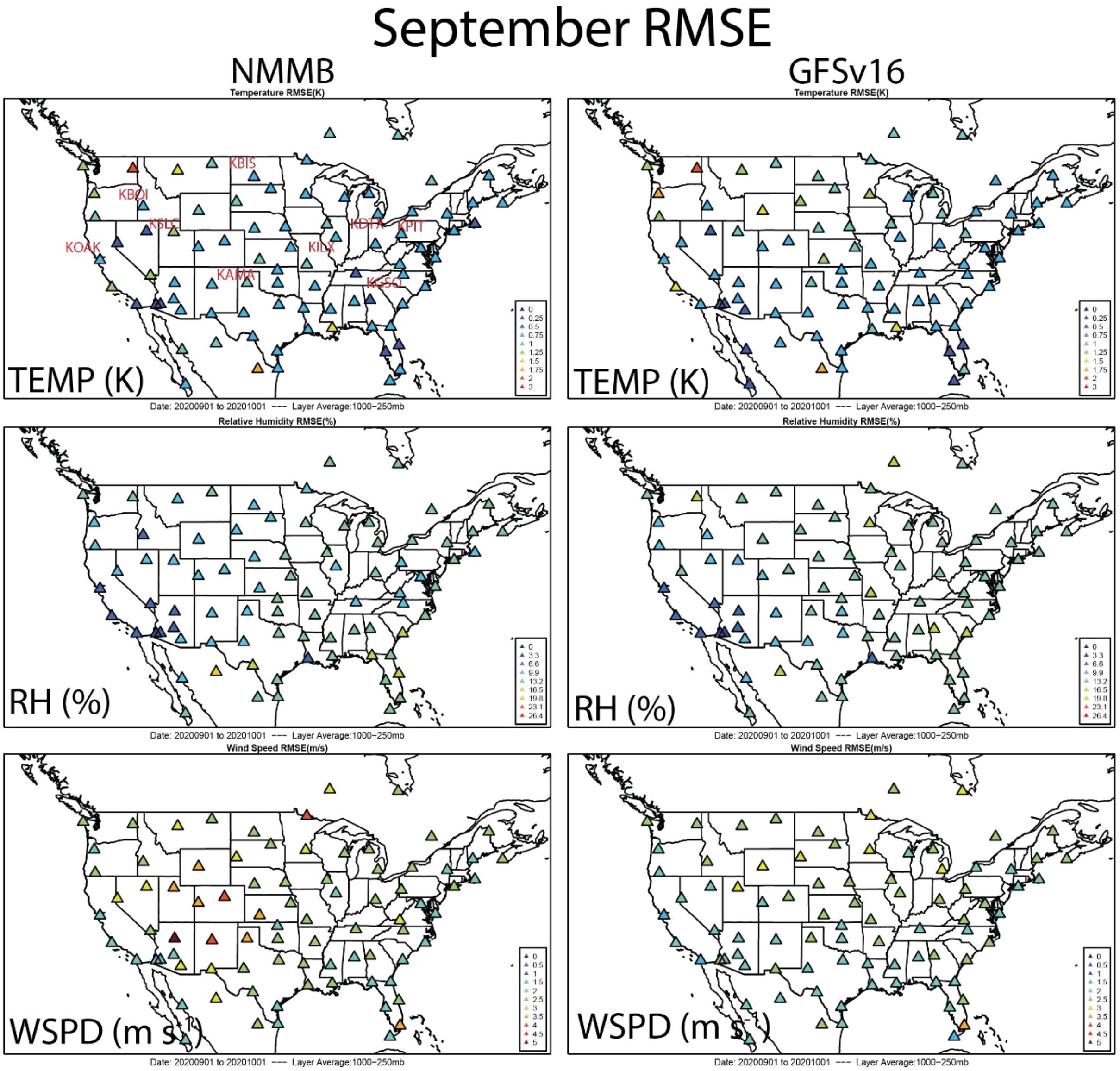

Figure S19a. Average September 2020 spatial root mean square (RMSE) comparison of the 1000-250 layer average NMMB (left) and GFSv16 (right) modeled temperature (TEMP; top), relative humidity (RH; middle), and wind speed (WSPD; bottom) compared against RAOB sites in CONUS. RAOB sites labeled in the top left panel pertain to the profile comparisons in the main text and Supporting Figures S15-S17.

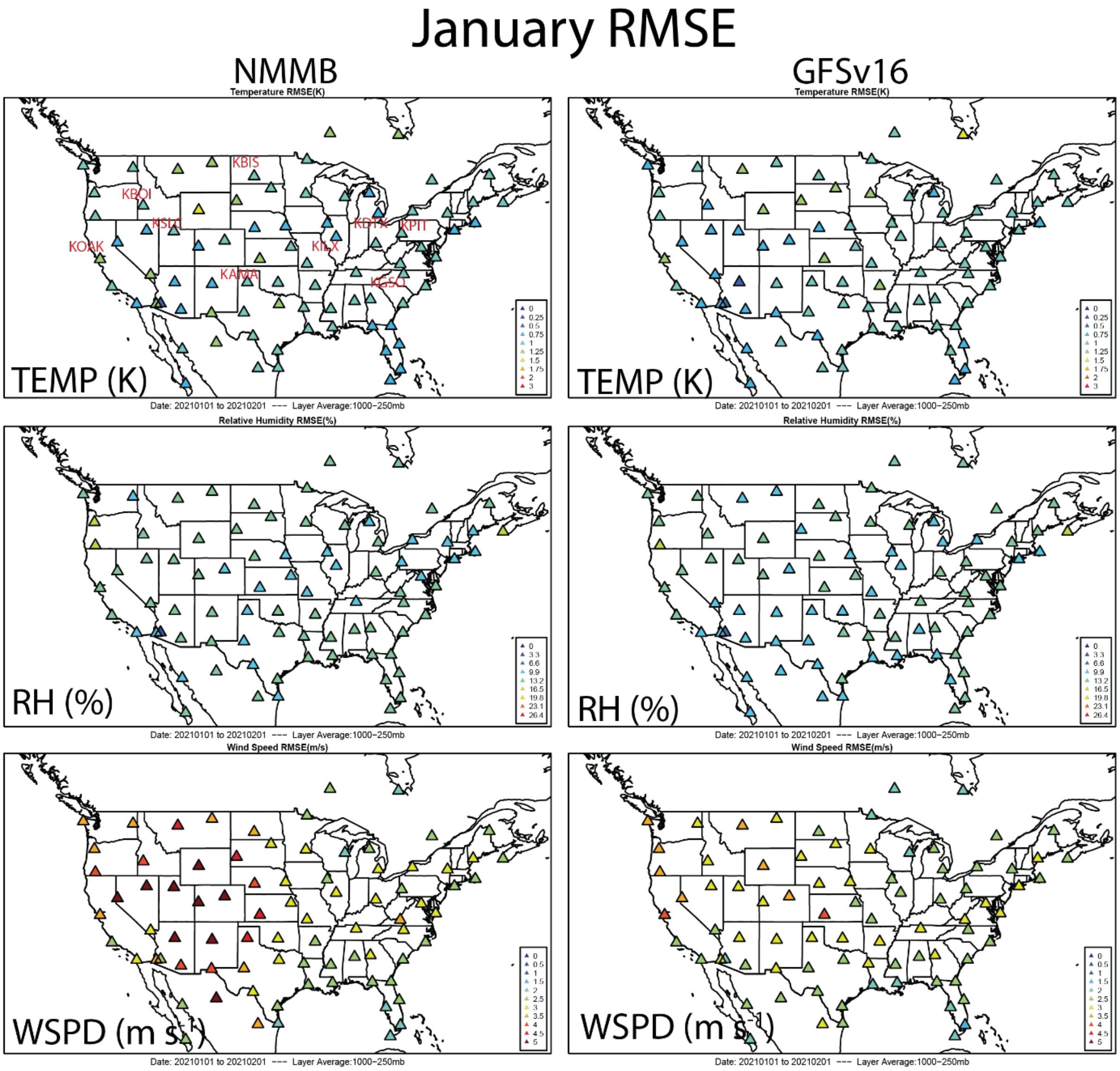

Figure S19b. Same as in Figure S19a, but for January 2021.

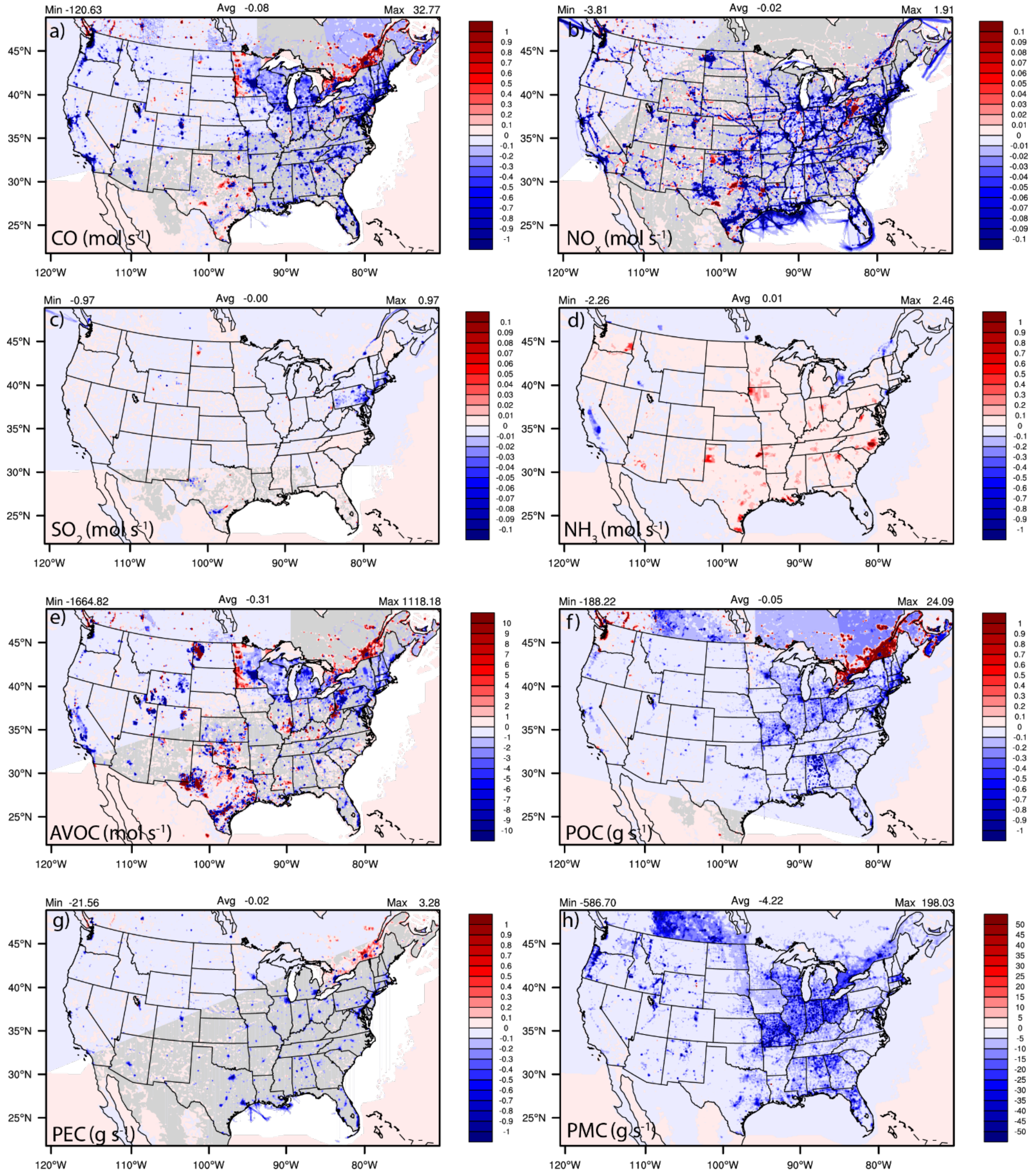

Figure S20. September 2020 average spatial difference plots for NEI 2016v1-NEI2014v2 combined 2D area/mobile emissions. Figure S20 shows the same analysis for January 2021.

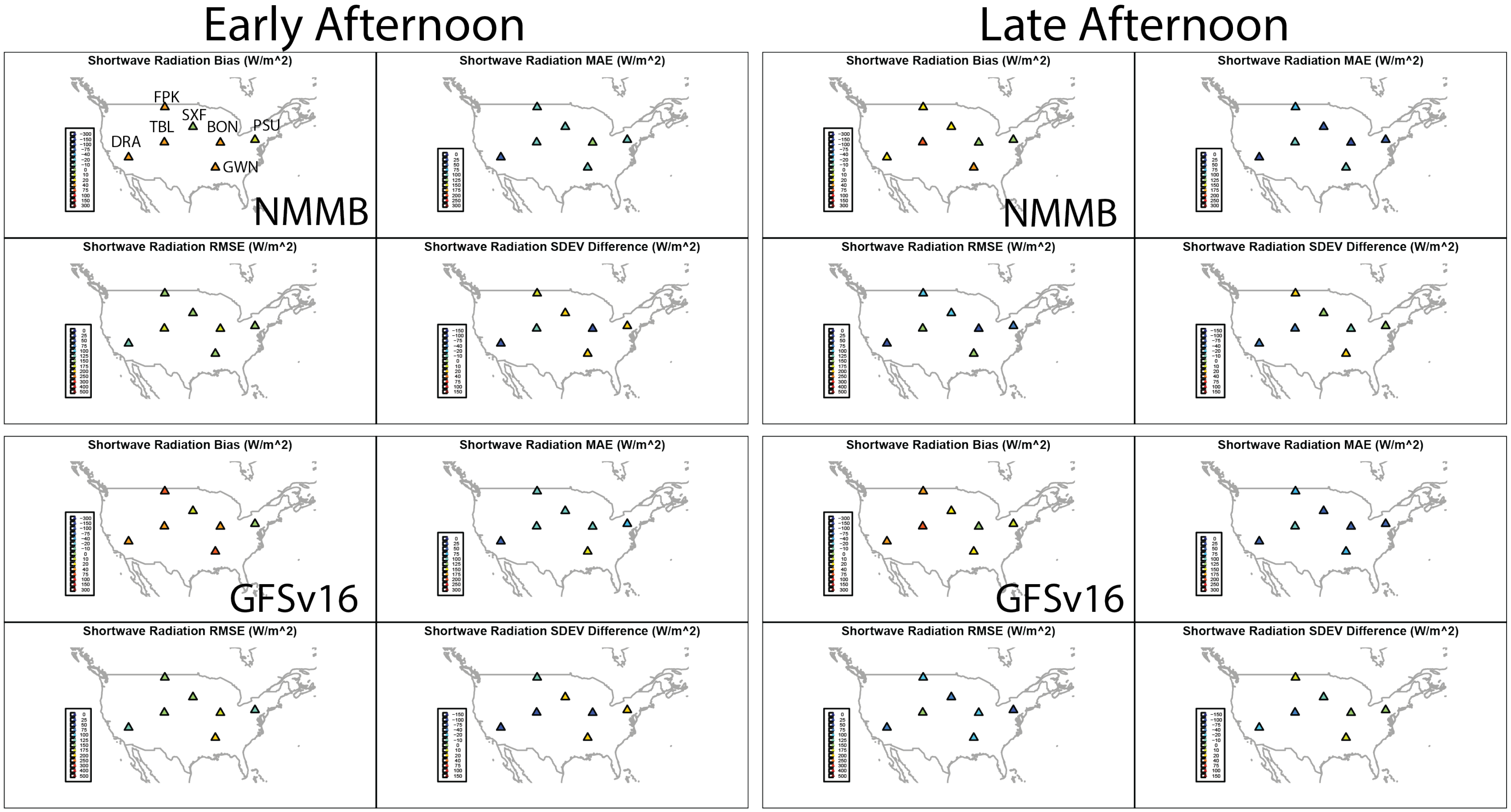

Figure S21. September 2020 spatial mean shortwave radiation bias, mean absolute error (MAE), root mean square error (RMSE), and standard deviation (SDEV) for early afternoon (left) and late afternoon (right) hours for NMMB (top) and GFSv16 (bottom) against the available eight BSRN-SURFRAD sites in CONUS. The SURFRAD site locations are labeled in the top left panel.

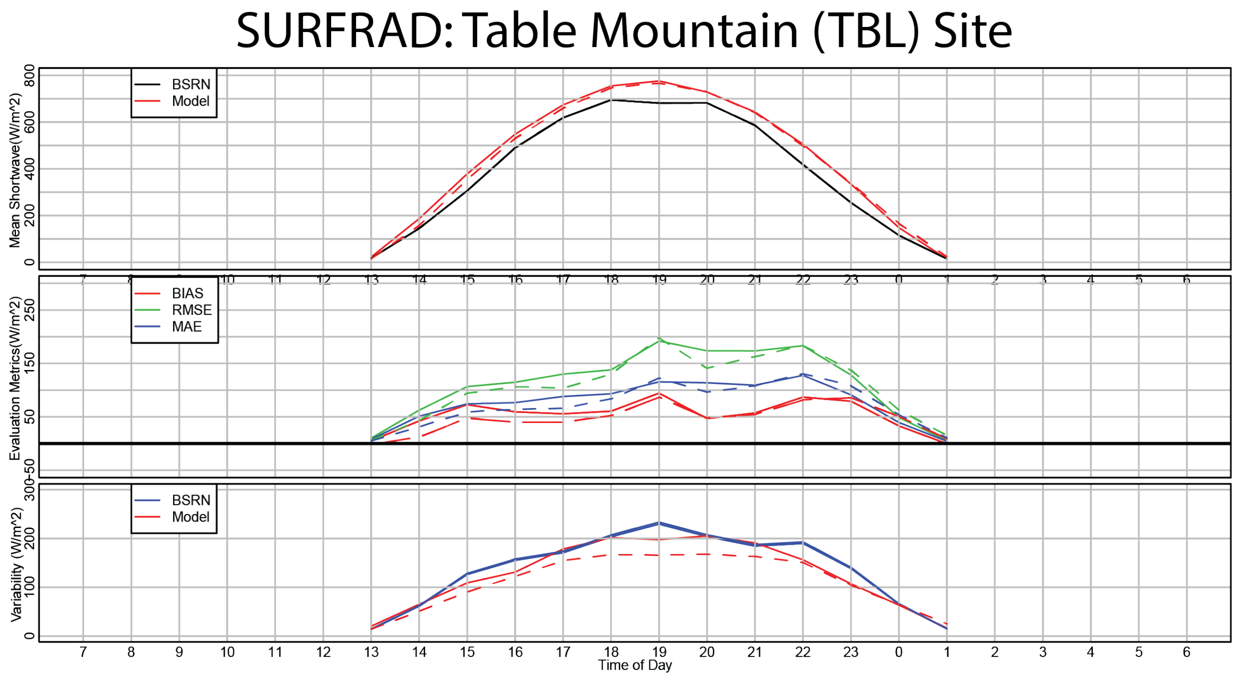

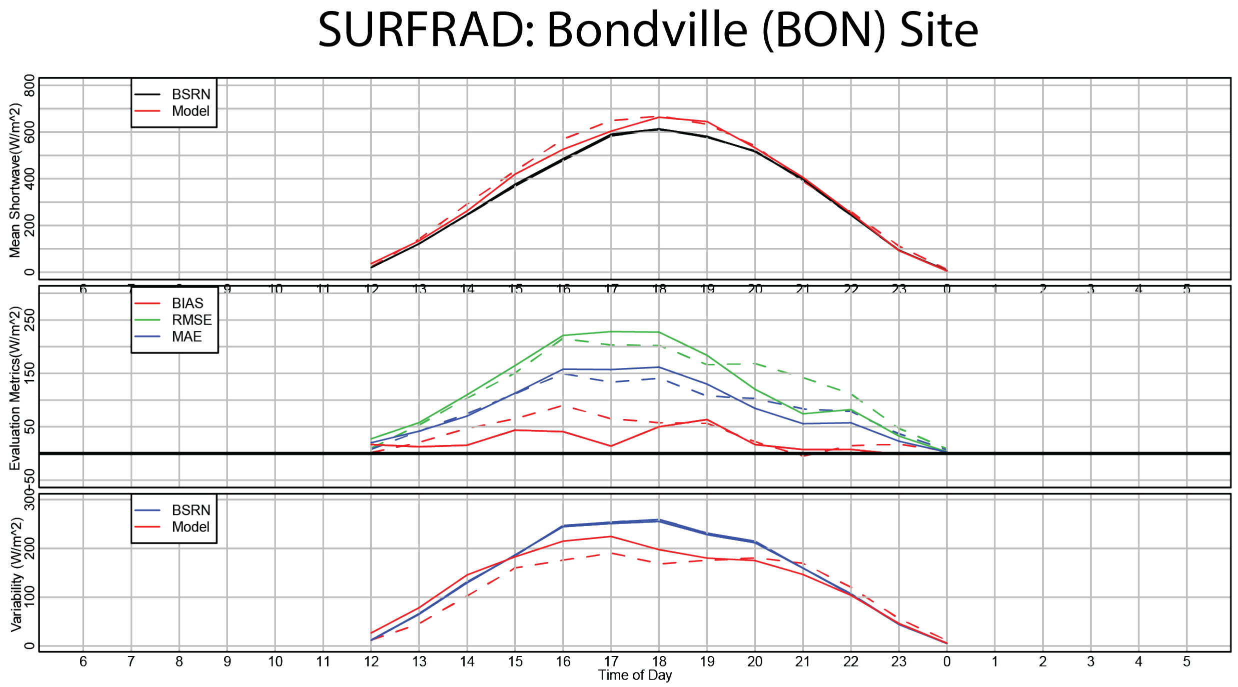

Figure S22. September 2020 diurnal mean shortwave radiation (top), evaluation metrics (middle), and variability (bottom) for the NMMB (solid) and GFSv16 (dashed) compared to the SURFRAD TBL and BON sites. Site locations are shown in Figure S21.

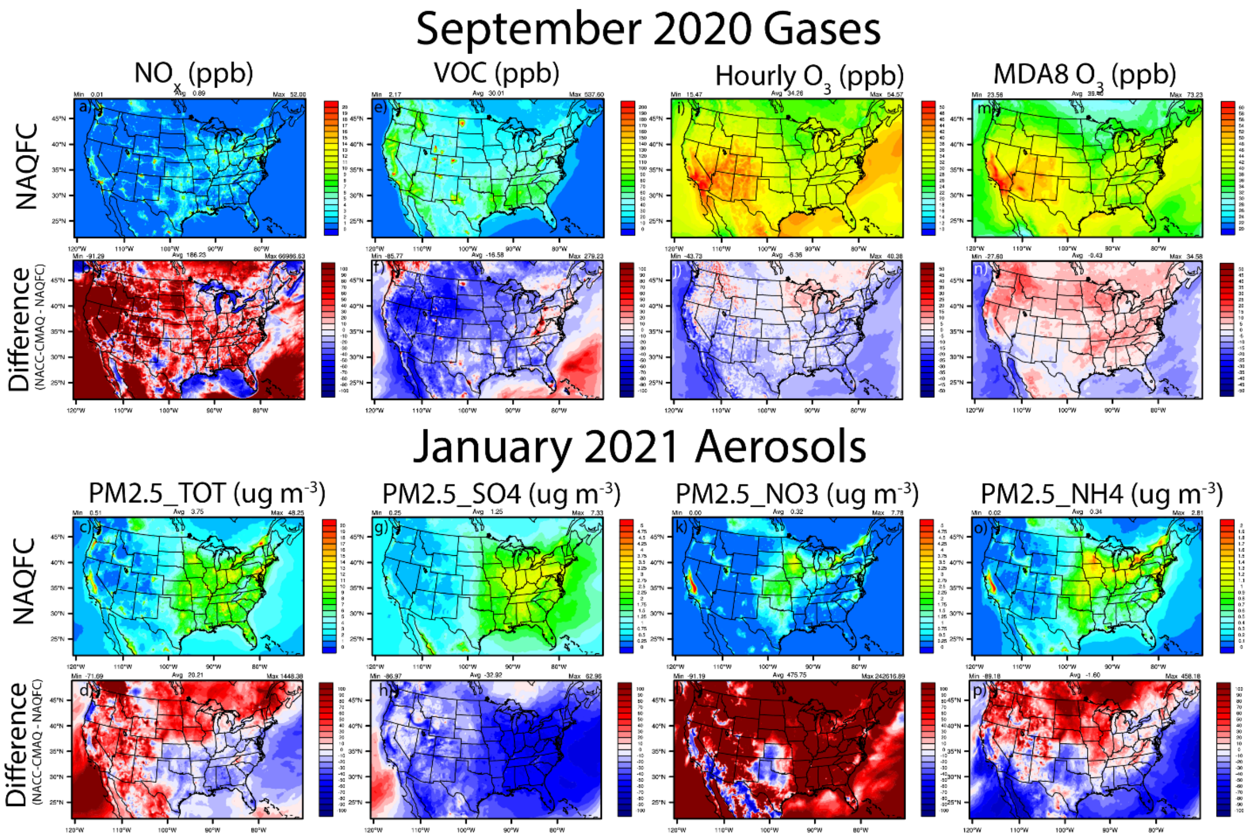

Figure S23. Average September 2020 NOx, total VOC, hourly O3, and MDA8 O3 and January 2021 PM2.5_TOT, PM2.5_SO4, PM2.5_NO3, and PM2.5_NH4 spatial plots for the operational NAQFC, and the relative (%) differences for NACC-CMAQ - NAQC.

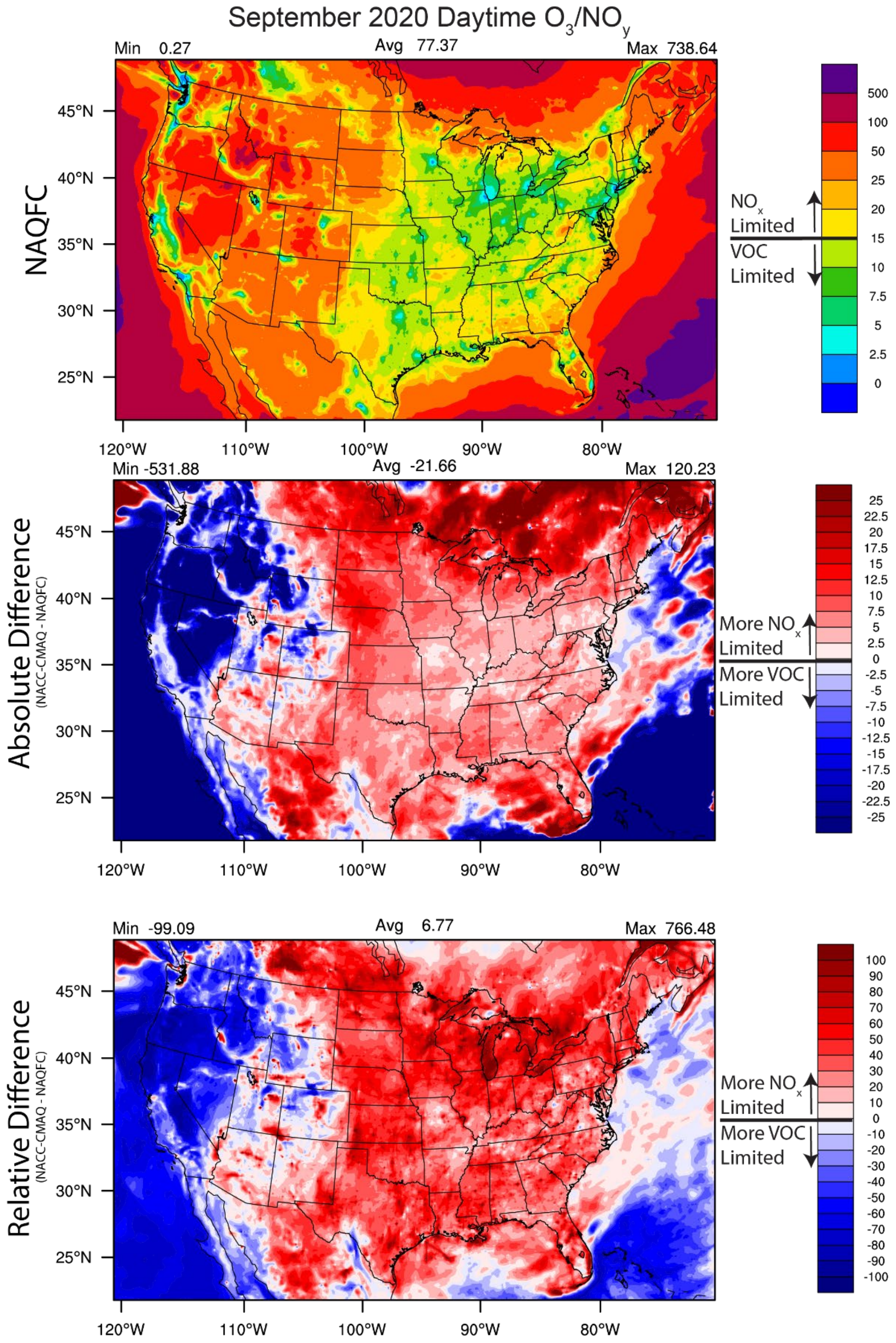

Figure S24. NAQFC simulated daytime (estimated using 17 – 23 UTC hours only) average O_3/NO_Y indicator ratio for September 2020, and the absolute and relative (5) change for NACC-CMAQ – NAQFC.

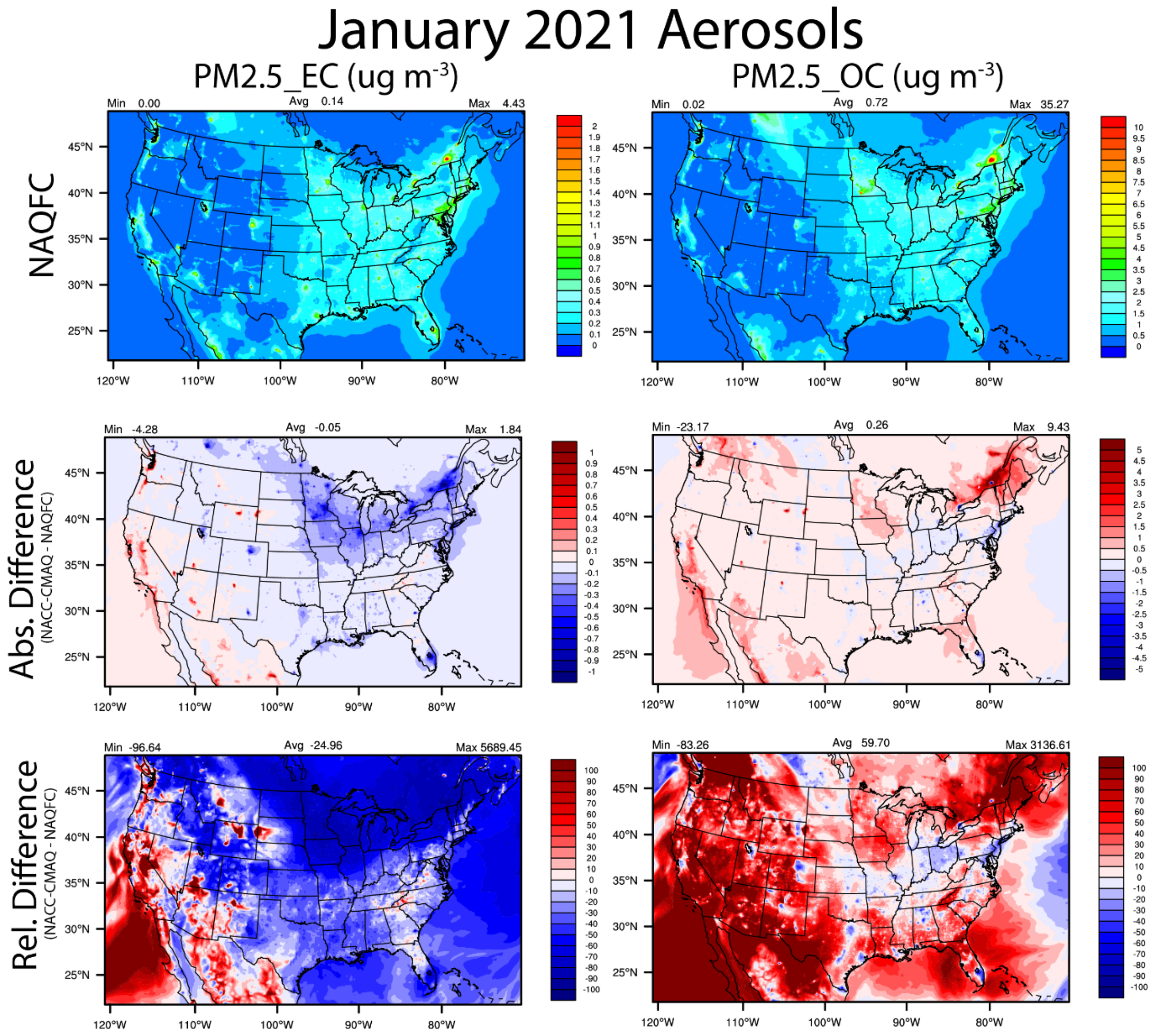

Figure S25. Average January 2021 PM25_EC and PM25_OC spatial plots for operational NAQFC and the absolute and relative (%) differences for NACC-CMAQ-NAQFC.

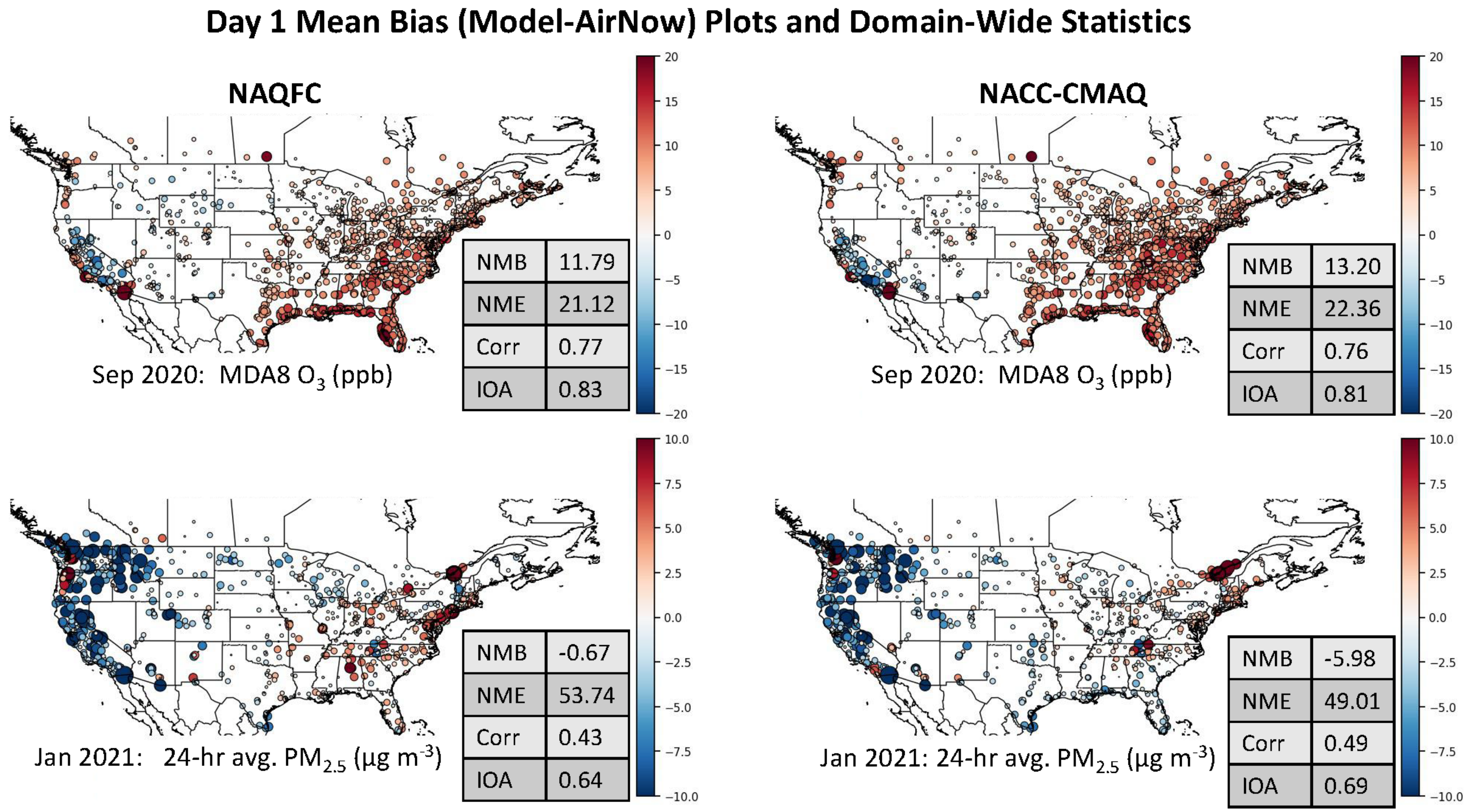

Figure S26. Day 1 forecast mean bias plots (model-AirNow) for the current operational NAQFC (left) and NACC-CMAQ (right) MDA8 O_3 (top) and 24-hr average $PM_{2.5}$ (bottom) in a)-b) September 2020 and c)-d) January 2021. Average domain-wide statistics are shown in the tables on the bottom left of each panel.

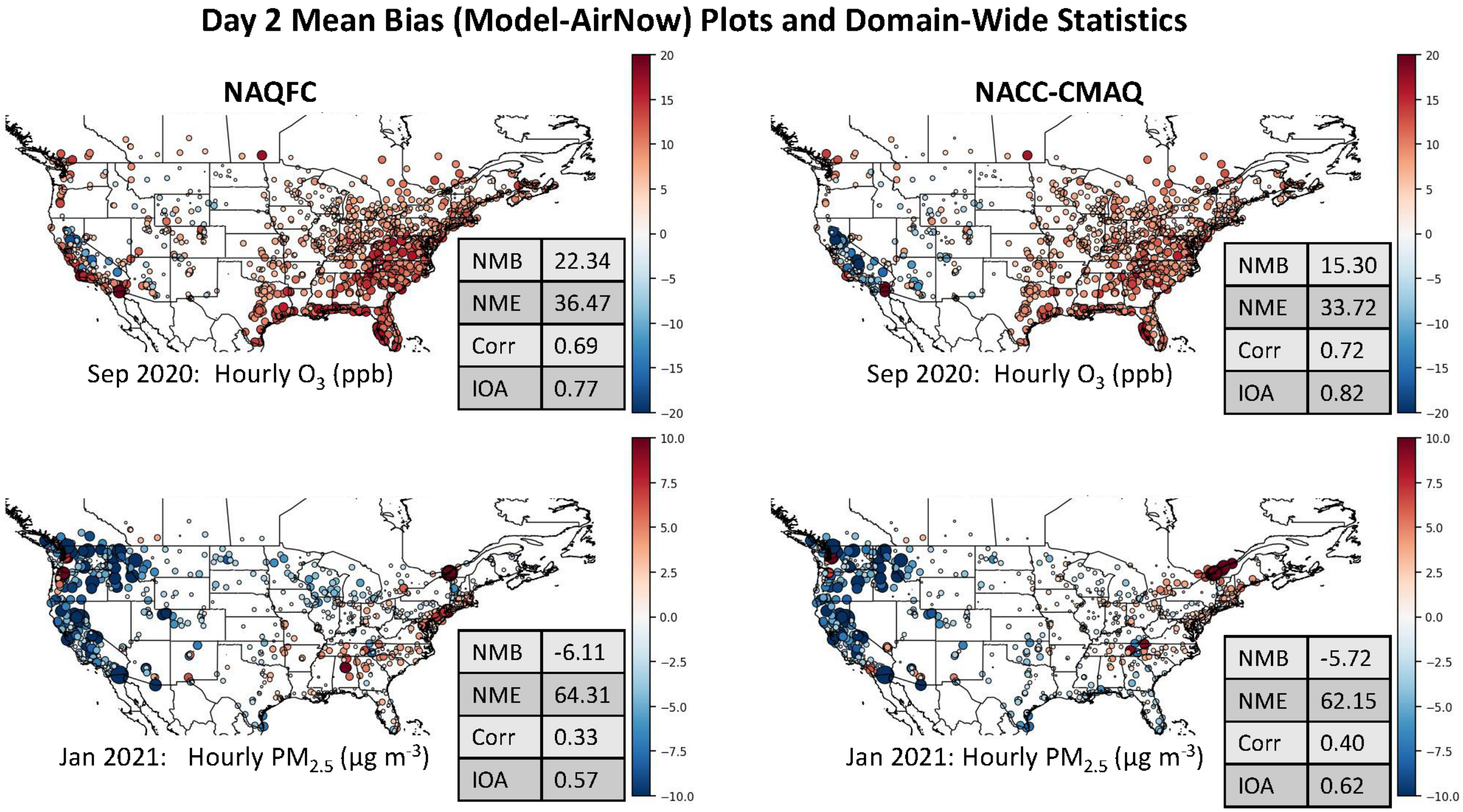

Figure S27. Same as in Figure S26, but for Day 2 forecasted hourly O_3 and $PM_{2.5}$

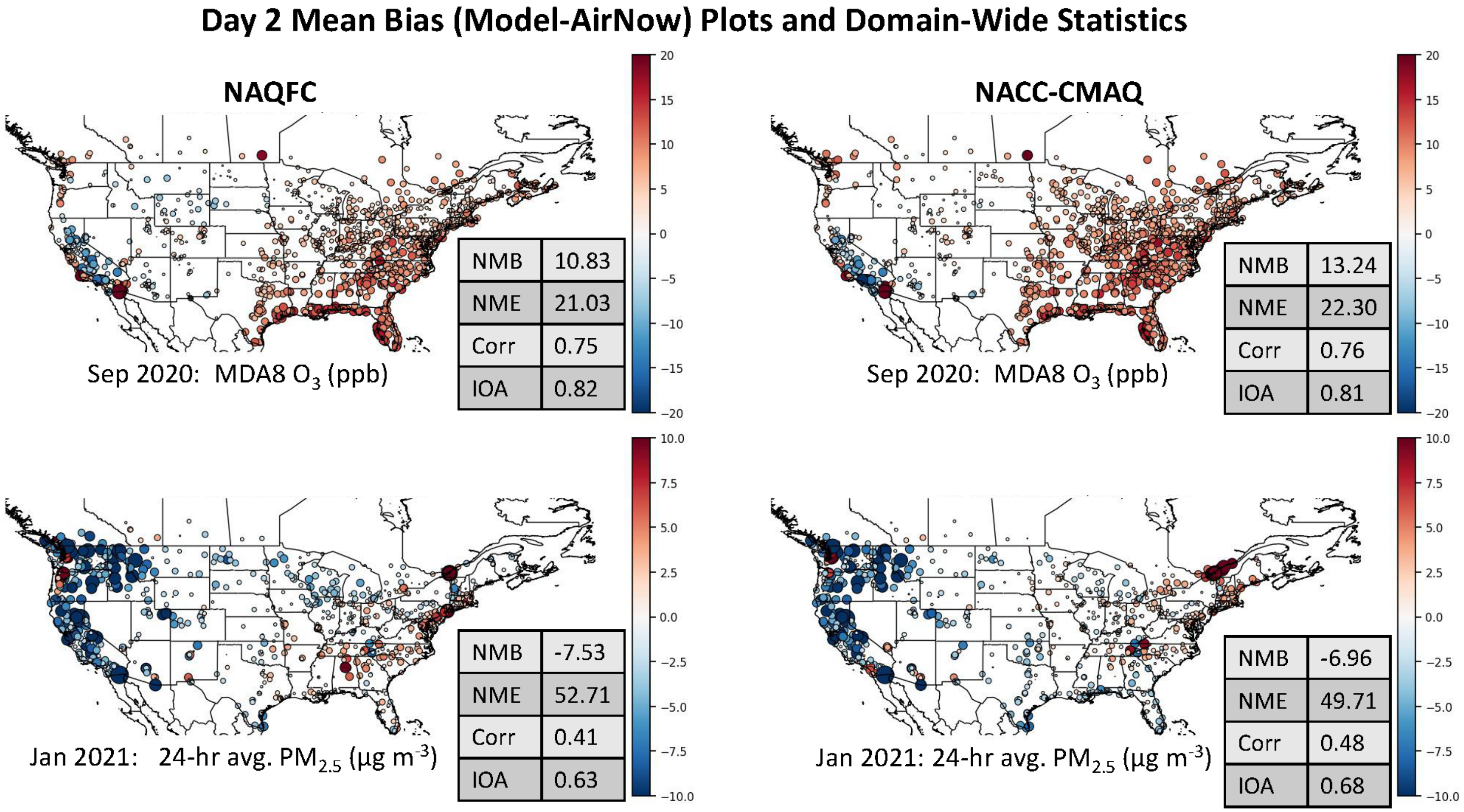

Figure S28. Same as in Figure S27, but for MDA8 O_3 and 24-hr average $PM_{2.5}$.

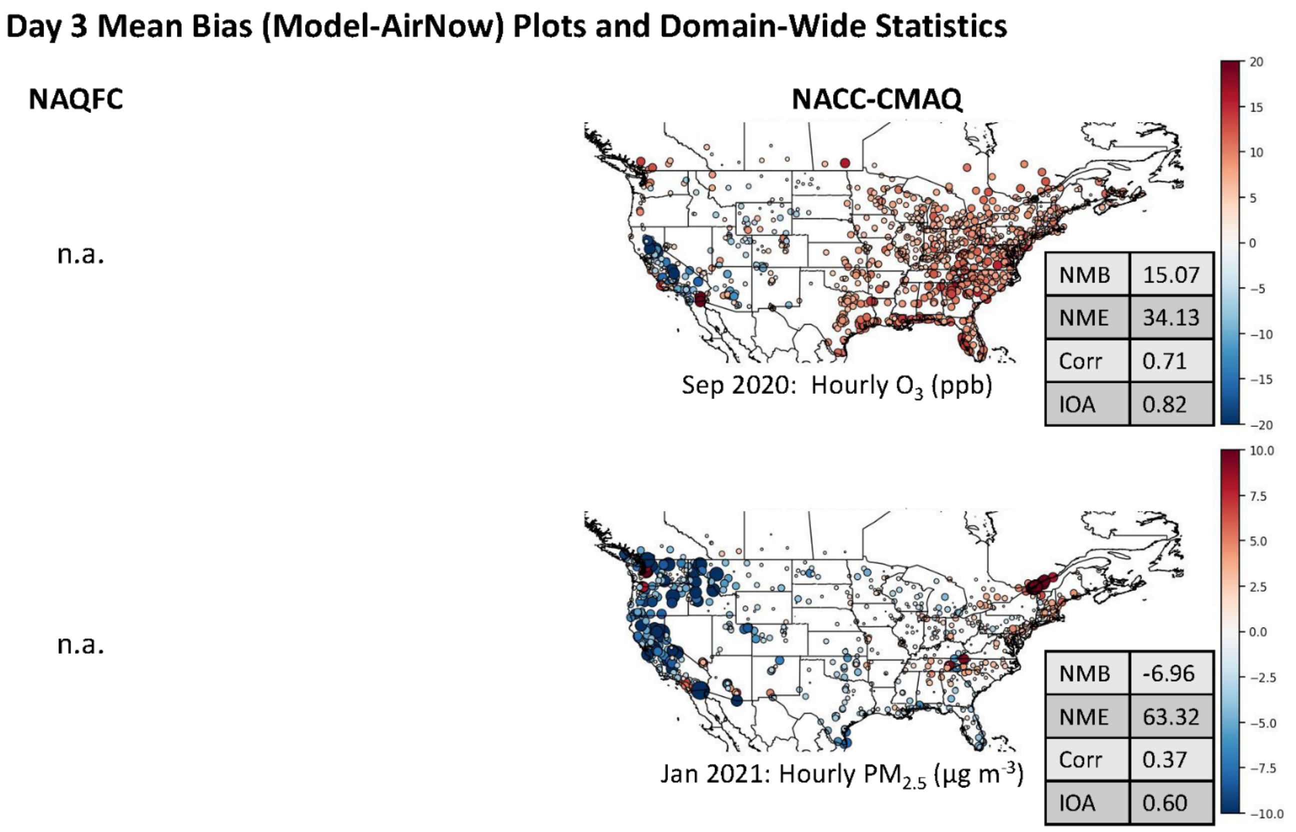

Figure S29. Same as in Figure S27, but for Day 3 forecasts (NACC-CMAQ only).

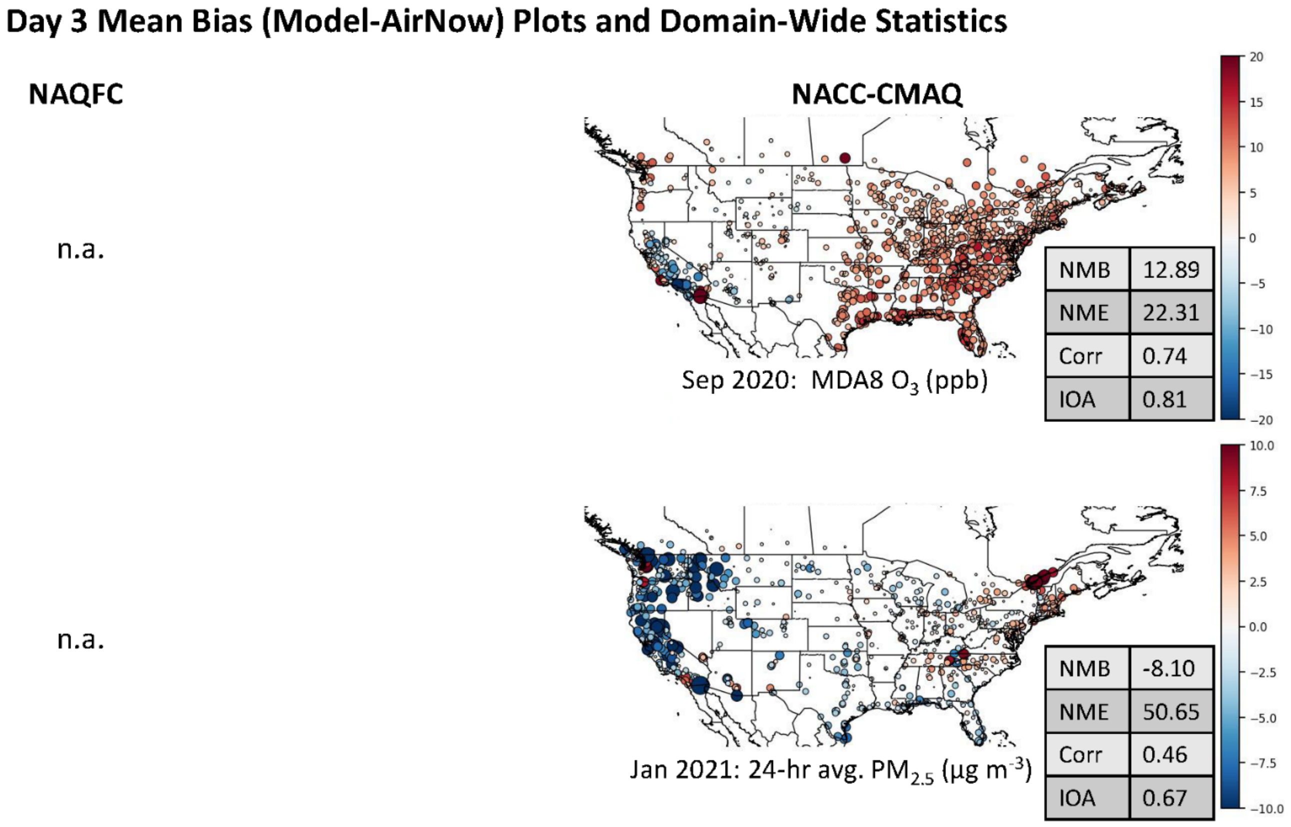

Figure S30. Same as in Figure S29, but for MDA8 O_3 and 24-hr average $PM_{2.5}$.

Table S1a. December 2020 average statistical performance of hourly ozone for the base simulation without vegetation frost switch (BASE), with frost switch and BELD3 (FROST3), and with frost switch and BELD5 (FROST5). The regions are defined by the U.S. EPA geographic regions (https://www.epa.gov/aboutepa/regional-and-geographic-offices).

Regions	R1	R2	R3	R4	R5	R6	R7	R8	R9	R10
**Normalized Mean Bias (%)**										
**BASE**	+11.3	+14.2	+11.5	+14.6	+23.5	+4.3	+16.3	+11.1	+5.1	-6.6
**FROST3**	+10.7	+13.4	+10.5	+14.3	+22.2	+3.8	+14.9	+9.9	+5.0	-6.8
**FROST5**	+9.8	+11.8	+8.4	+13.2	+19.3	+1.6	+12.2	+8.0	+2.3	-7.5
**Normalized Mean Error (%)**										
**BASE**	25.6	27.5	26.9	28.9	32.8	28.8	34.9	29.2	37.1	31.3
**FROST3**	25.4	27.1	26.5	28.8	31.9	28.6	34.0	28.8	37.1	31.3
**FROST5**	25.1	26.7	26.3	28.4	31.0	28.7	33.9	28.2	36.8	31.3
**Correlation**										
**BASE**	0.65	0.70	0.64	0.69	0.66	0.71	0.64	0.56	0.64	0.73
**FROST3**	0.66	0.71	0.64	0.69	0.66	0.71	0.65	0.57	0.64	0.73
**FROST5**	0.66	0.71	0.64	0.69	0.65	0.70	0.63	0.57	0.64	0.73
**Index of Agreement**										
**BASE**	0.78	0.81	0.78	0.79	0.75	0.83	0.77	0.74	0.80	0.85
**FROST3**	0.78	0.82	0.78	0.79	0.76	0.83	0.78	0.74	0.80	0.85
**FROST5**	0.79	0.82	0.79	0.79	0.76	0.83	0.78	0.75	0.80	0.85

Table S1b. Same as in Table S1a, but for hourly $PM_{2.5}$.

Regions	R1	R2	R3	R4	R5	R6	R7	R8	R9	R10
**Normalized Mean Bias (%)**										
**BASE**	+30.0	+55.3	+28.9	+7.9	+21.1	-1.1	+28.5	-17.4	-14.9	-6.2
**FROST3**	+27.4	+53.3	+26.9	+7.0	+19.1	-1.9	+26.7	-19.1	-15.3	-7.0
**FROST5**	+24.4	+50.2	+23.1	+0.8	+15.6	-9.0	+21.6	-27.4	-24.3	-14.1
**Normalized Mean Error (%)**										
**BASE**	67.9	88.3	60.7	50.9	53.1	61.2	59.5	66.1	63.7	81.5
**FROST3**	66.6	86.9	59.3	50.5	52.1	60.8	58.5	65.7	63.6	81.1
**FROST5**	65.1	85.0	57.7	48.2	50.6	58.6	56.4	64.6	61.7	79.9
**Correlation**										
**BASE**	0.61	0.53	0.63	0.49	0.62	0.32	0.57	0.34	0.39	0.30
**FROST3**	0.61	0.53	0.63	0.49	0.63	0.32	0.57	0.34	0.39	0.30
**FROST5**	0.61	0.52	0.62	0.49	0.62	0.32	0.56	0.34	0.40	0.28
**Index of Agreement**										
**BASE**	0.72	0.54	0.72	0.68	0.75	0.56	0.71	0.57	0.61	0.54
**FROST3**	0.73	0.55	0.72	0.68	0.76	0.56	0.71	0.57	0.61	0.54
**FROST5**	0.73	0.55	0.72	0.68	0.76	0.55	0.71	0.56	0.60	0.53

Table S2. Operational NAQFC (NMMB-CMAQv5.0.2) model components and configurations (Adapted from Lee et al., 2017). References are found below the table.

Model Attribute	Configuration	Reference
Domain	Contiguous U.S.;<br>Center = 33°N;97°W	n/a
Horizontal Resolution	12 km	n/a
Vertical Resolution	35 Layers from near-surface to about 14 km (~ 60 hPa)	n/a
Meteorological ICs and BCs	NMMB	*Black, 1994; Janjic and Gall, 2012*
Chemical ICs and BCs	2006 GEOS-Chem Simulation<br>&<br>NGAC Dust Ony CLBCs	http://acmg.seas.harvard.edu/geos/<br><br>Tang et al. (2021)
Anthropogenic Emissions	NEI2014v2	*NEI (2014)*
Biogenic Emissions	Inline BEISv3.1.4 & BELD3	*Vukovich and Pierce* (2002); *Schwede et al.* (2005)
Wildfire Emissions/Plume Rise	NOAA/NESDIS Hazard Mapping System-Bluesky Methodology/<br><br>Inline Briggs	*Ruminski et al., 2008; Schroeder et al., 2008; Ruminski and Kondragunta, 2006; O'Neill et al. (2009); Pan et al. (2020)*<br>*Briggs* (1965)
Microphysics	Ferrier-Aligo	*Aligo et al. (2014)*
PBL Physics Scheme	Mellor-Yamada-Janjic (MYJ)<br>ACM2 (CMAQ recalculated)	*Janjic et al. (2001)*<br>*Pleim (2007a;2007b)*
Shallow/Deep Cumulus Parameterization	Betts-Miller-Janjic	*Janjic (2000)*
Shortwave and Longwave Radiation	RRTMg	*Mlawer et al. (1997); Clough et al.* (2005); *Iacono et al.* (2008)
Land Surface Model	Noah Land Surface Model	*Chen and Dudhia (2001), Ek et al. (2003),Tewari et al. (2004)*
Surface Layer	Monin-Obukhov	*Monin-Obukhov* (1954); *Grell et al.* (1994); *Jimenez et al.* (2012)
Gas-phase Chemistry	CB05	*Yarwood et al., 2005*
Aqueous-phase Chemistry	CMAQ AQCHem Updates	*Martin and Good* (1991)*; Alexander et al.* (2009)*; Sarwar et al.* (2011)
Aerosol Module/Size	AERO4	*Binkowski and Roselle (2003)*
Other Model Attributes	-In-line Photolysis<br>-Offline FENGSHA Wind-Blown Dust Emissions<br>-In-line Sea-salt Emissions	*Binkowski et al.* (2007)<br>Fu et al., 2014; Huang et al., 2015; Dong et al., 2016<br>*Kelly et al.* (2010)

References:

- Alexander, B., Park, R. J., Jacob, D. J., and Gong, S.: Transition metal-catalyzed oxidation of atmospheric sulfur: global implications for the sulfur budget, *J. Geophys. Res.*, 114, D02309. https://doi.org/10.1029/2008JD010486. 2009.
- Aligo, E., Ferrier, B.S., Rogers, E., Pyle, M., Weiss, S. J., and Jirak, I. L.: Modified microphysics for use in high-resolution NAM forecasts. Proc. 27th Conf. on Severe Local Storms, Madison, WI, Amer. Meteor. Soc., 16A.1. [Available online at https://ams.confex.com/ams/27SLS/webprogram/Paper255732.html]. 2014.
- Binkowski, F. S. and Roselle, S. J.: Models-3 Community Multiscale Air Quality (CMAQ) model aerosol component 1. Model description. *J. Geophys. Res*., 108, 4183, https://doi.org/10.1029/2001JD001409. 2003.
- Binkowski, F. S, Arunachalam, S., Adelman, Z., and Pinto, J.: Examining photolysis rates with a prototype on-line photolysis module in CMAQ. *J. Appl. Meteor. and Clim*. 46, 1252-1256. https://doi.org/10.1175/JAM2531.1. 2007.
- Black, T. L.: The new NMC meso-scale Eta Model: description and forecast examples. Weather and Forecasting. 9, 265-278. https://doi.org/10.1175/1520-0434(1994)009<0265:TNNMEM>2.0.CO;2. 1994.
- Briggs, G. A.: A plume rise model compared with observations. Journal of the Air Pollution Control Association. 15 (9), 433-438. https://doi.org/10.1080/00022470.1965.10468404. 1965.

- Chen, F. and Dudhia, J.: Coupling an advanced land surface-hydrology model with the Penn State-NCAR MM5 modeling system. Part I: Model implementation and sensitivity. *Monthly Weather Review*, 129(4), 569–585. https://doi.org/10.1175/1520-0493(2001)129<0569:CAALSH>2.0.CO;2. 2001.
- Clough, S. A., Shephard, M. W., Mlawer, J. E., Delamere, J. S., Iacono, M. J., Cady-Pereira, K. et al.: Atmospheric radiative transfer modeling: A summary of the AER codes. Journal of Quantitative Spectroscopy & Radiative Transfer, 91(2), 233–244. https://doi.org/10.1016/j.jqsrt.2004.05.058 2005.
- Dong, X., Fu, J. S., Huang, K., Tong, D., and Zhuang, G.: Model development of dust emission and heterogeneous chemistry within the Community Multiscale Air Quality modeling system and its application over East Asia. *Atmos. Chem. Phys*., 16, 8157-8180. https://doi.org/10.5194/acp-16-8157-2016. 2016.
- Ek, M., Mitchell, B. K. E., Lin, Y., Rogers, E., Grunmann, P., Koren, V. et al.: Implementation of Noah land surface model advances in the National Centers for Environmental Prediction operational mesoscale Eta model. *Journal of Geophysical Research*, 108(D22), 8851. https://doi.org/10.1029/2002JD003296. 2003.
- Fu X, Wang, S., Cheng, Z., Xing, J., Zhao, B., Wang, J., and Hao, J.: Source, transport and impacts of a heavy dust event in the Yangtze River Delta, China, in 2011. Atmos. Chem. Phys. 14, 1239–54. https://doi.org/10.5194/acp-14-1239-2014.
- Grell, G. A., Dudhia, J., and Stauffer, D. R.: A description of the fifth-generation Penn State/NCAR Mesoscale Model (MM5). NCAR tech. Note NCAR TN-398-1-STR, 117 pp. 1994.
- Huang, M., Tong, D., Lee, P., Pan, L., Tang, Y., Stajner, I. et al.: Toward enhanced capability for detecting and predicting dust events in the western United States: The Arizona case study. *Atmos. Chem. Phys*., 15, 12 595–12 610. https://doi.org/10.5194/acp-15-12595-2015. 2015.
- Iacono, M. J., Delamere, J. S., Mlawer, E. J., Shephard, M. W., Clough, S. A., and Collins, W. B.: Radiative forcing by long-lived greenhouse gases: Calculations with the AER radiative transfer models. *Journal of Geophysical Research*, 113, D13103. https://doi.org/10.1029/2008JD009944 2008.
- Janjić, Z. I.: Comments on “Development and evaluation of a convection scheme for use in climate models.” J. Atmos. Sci., 57, 3686. https://doi.org/10.1175/1520-0469(2000)057<3686:CODAEO>2.0.CO;2. 2000.
- Janjic, Z., and Gall, R. L.: Scientific documentation of the NCEP nonhydrostatic multiscale model on the B grid (NMMB). Part 1 Dynamics (No. NCAR/TN-489+STR). University Corporation for Atmospheric Research. https://doi.org/10.5065/D6WH2MZX. 2012.
- Janjić, Z. I., Gerrity Jr., J. P., and Nickovic, S.: An alternative approach to nonhydrostatic modeling. Mon. Wea. Rev., 129, 1164–1178, https://doi.org/10.1175/1520-0493(2001)129<1164:AAATNM>2.0.CO;2. 2001.
- Jimenez, P. A., Dudhia, J., Gonzalez-Rouco, J. F., Navarro, J., Montavez, J. P., and Garcia-Bustamante, E.: A revised scheme for the WRF surface layer formulation. *Monthly Weather Review*, 140(3), 898–918. https://doi.org/10.1175/MWR-D-11-00056.1. 2012.
- Kelly, J. T., Bhave, P. V., Nolte, C. G., Shankar, U., and Foley, K. M.: Simulating emission and chemical evolution of coarse sea-salt particles in the Community Multiscale Air Quality (CMAQ) model. *Geosci. Model Dev*., 3, 257–273. https://doi.org/10.5194/gmd-3-257-2010. 2010.
- Martin, R. L. and Good, T. W.: Catalyzed oxidation of sulfur dioxide in solution: the iron-manganese synergism. *Atmospheric Environment*. 25A, 2395-2399. https://doi.org/10.1016/0960-1686(91)90113-L. 1991.
- Mlawer, E. J., Taubman, S. J., Brown, P. D., Iacono, M. J., and Clough, S. A.: Radiative transfer for inhomogeneous atmosphere: RTTM, a validated correlated-k model for the longwave. J. Geophys. Res., 102, 16 663–16 682. https://doi.org/10.1029/97JD00237. 1997.
- Monin, A. S., and Obukhov, A. M.: Basic laws of turbulent mixing in the surface layer of the atmosphere (in Russian). *Tr. Akad. Nauk SSSR Geophiz. Inst.* 24(151), 151, 163–187. 1954.
- National Emissions Inventory (NEI).: [Data set]. Retrieved February 26, 2020, from https://edap.epa.gov/public/extensions/nei_report_2014/dashboard.html#sector-db. 2014.
- O’Neill, S. M., Larkin, N. K., Hoadley, J., Mills, G., Vaughan, J. K., Draxler, R. R. et al.: Regional real-time smoke prediction systems. Wildland Fires and Air Pollution, A. Bytnerowicz et al., Eds., Developments in Environmental Science, Vol. 8, Elsevier, 499–534.2009.
- Pan, L., Kim, H., Lee, P., Saylor, R., Tang, Y., Tong, D., et. al.: Evaluating a fire smoke simulation algorithm in the National Air Quality Forecast Capability (NAQFC) by using multiple observation data sets during the Southeast Nexus (SENEX) field campaign. Geosci. Model Dev., 13, 2169–2184. https://doi.org/10.5194/gmd-13-2169-2020. 2020.
- Pleim, J. E.: A combined local and nonlocal closure model for the atmospheric boundary layer. Part I: Model description and testing. J. Appl. Meteor. Climatol. 46, 1383–1395, https://doi.org/10.1175/JAM2539.1. 2007a.
- Pleim, J. E.: A combined local and nonlocal closure model for the atmospheric boundary layer. Part II: Application and evaluation in a mesoscale meteorological model. J. Appl. Meteor. Climatol. 46, 1396–1409. https://doi.org/10.1175/JAM2534.1. 2007b.
- Ruminski, M, and Kondragunta, S.: Monitoring fire and smoke emissions with the hazard mapping system. Proceedings Volume 6412. Disaster Forewarning Diagnostic Methods and Management. https://doi.org/10.1117/12.694183. 2006.
- Ruminski, M., Simko, J., Kibler, J., Kondragunta, S., Draxler, R., Davidson, P., and Li, P.: Use of multiple satellite sensors in NOAA's operational near real-time fire and smoke detection and characterization program. Remote Sensing of Fire: Science and Application. https://doi.org/10.1117/12.807507. 2008.
- Sarwar, G., Fahey, K., Napelenok, S., Roselle, S., and Mathur, R.: Examining the impact of CMAQ model updates on aerosol sulfate predictions. The 10th Annual CMAS Models-3 User’s Conference, October, Chapel Hill, NC. 2011.
- Schroeder, W., Ruminski, M., Csiszar, I., Giglio, L., Prins, E., Schmidt, C., and Morisette, J.: Validation analyses of an operational fire monitoring product: The Hazard Mapping System, Int. J. Remote Sens., 29, 6059–6066. https://doi.org/10.1080/01431160802235845. 2008
- Schwede, D., Pouliot, G. A., and Pierce, T.: Changes to the Biogenic Emissions Inventory System Version 3 (BEIS3). In Proceedings of the 4th CMAS Models-3 Users’ Conference, Chapel Hill, NC, 26–28 September 2005. 2005.
- Tang, Y., H. Bian, Z. Tao, L. D. Oman, D. Tong, P. Lee, et al.: Comparison of chemical lateral boundary conditions for air quality predictions over the contiguous United States during pollutant intrusion events. Atmos. Chem. Phys. 21, 2527–2550. https://doi.org/10.5194/acp-21-2527-2021. 2021.

- Tewari, M., F. Chen, W. Wang, J. Dudhia, M. A. LeMone, K. Mitchell, et al.: Implementation and verification of the unified NOAH land surface model in the WRF model. Paper Presented at the 20th Conference on Weather Analysis and Forecasting/16th Conference on Numerical Weather Prediction, Seattle, WA. 2004.
- Vukovich, J. M., and T. Pierce: The Implementation of BEIS3 within the SMOKE modeling framework. Environmental Science. 2002.
- Yarwood, G., Rao, S., Yocke, M., and Whitten, G.: Updates to the carbon bond chemical mechanism: CB05. Final Rep. to the US EPA, RT-0400675. [Available online at http://www.camx.com/publ/pdfs/cb05_final_report_120805.pdf.] 2005

Table S3. September 2020 average statistical summary of TEMP2, Q2, and WSPD10 for NMMB and GFSv16 against the MADIS-METAR network for day 1, 2, and 3 (GFSv16) forecasts in the east (< 100° W) and west CONUS (> 100° W).

**TEMP2: September 2020-East U.S.**					**TEMP2: September 2020-West U.S.**				
**Day 1**	MB (K)	*NMB (%)	RMSE (K)	R	**Day 1**	MB (K)	*NMB (%)	RMSE (K)	R
	NMMB					*NMMB*			
	0.33	0.69	1.94	0.96		0.03	0.04	3.1	0.93
	GFSv16					*GFSv16*			
	0.1	0.2	1.92	0.96		0.93	1.46	3.34	0.92
**Day 2**	MB (K)	*NMB (%)	RMSE (K)	R	**Day 2**	MB (K)	*NMB (%)	RMSE (K)	R
	NMMB					NMMB			
	0.24	0.5	2.2	0.95		0.04	0.06	3.39	0.91
	GFSv16					*GFSv16*			
	0.24	0.48	2.15	0.95		1.08	1.58	3.5	0.92
**Day 3**	MB (K)	NMB (%)	RMSE (K)	R	**Day 3**	MB (K)	NMB (%)	RMSE (K)	R
	NMMB					*NMMB*			
	n/a	n/a	n/a	n/a		n/a	n/a	n/a	n/a
	GFSv16					*GFSv16*			
	0.35	0.71	2.34	0.94		1.15	1.68	3.6	0.92
**Q2: September 2020-East U.S.**					**Q2: September 2020-West U.S.**				
**Day 1**	MB (g/kg)	*NMB (%)	RMSE (g/kg)	R	**Day 1**	MB (g/kg)	*NMB (%)	RMSE (g/kg)	R
	NMMB					*NMMB*			
	-0.2	-0.68	1.25	0.96		-0.07	-0.26	1.27	0.9
	GFSv16					*GFSv16*			
	-1.35	-4.85	1.97	0.95		-0.89	-3.19	1.64	0.88
**Day 2**	MB	*NMB (%)	RMSE	R	**Day 2**	MB (g/kg)	*NMB (%)	RMSE (g/kg)	R
	NMMB					*NMMB*			
	-0.33	-1.12	1.46	0.95		-0.17	-0.61	1.47	0.86
	GFSv16					*GFSv16*			
	-1.33	-4.79	2.05	0.94		-0.94	-3.34	1.72	0.86
**Day 3**	MB (g/kg)	*NMB (%)	RMSE (g/kg)	R	**Day 3**	MB (g/kg)	*NMB (%)	RMSE (g/kg)	R
	NMMB					*NMMB*			
	n/a	n/a	n/a	n/a		n/a	n/a	n/a	n/a
	GFSv16					*GFSv16*			
	-1.21	-4.34	2.09	0.93		-0.94	-3.36	1.76	0.86
**WSPD10: September 2020-East U.S.**					**WSPD10: September 2020-West U.S.**				
**Day 1**	MB (m/s)	*NMB (%)	RMSE (m/s)	R	**Day 1**	MB (m/s)	*NMB (%)	RMSE (m/s)	R
	NMMB					*NMMB*			
	0.36	0.7	1.6	0.7		-0.47	-1.33	1.88	0.69
	GFSv16					*GFSv16*			
	0.52	1	1.69	0.69		-0.21	-0.59	1.96	0.69
**Day 2**	MB (m/s)	*NMB (%)	RMSE (m/s)	R	**Day 2**	MB (m/s)	*NMB (%)	RMSE (m/s)	R
	NMMB					*NMMB*			
	0.36	0.69	1.71	0.65		-0.46	-1.3	1.98	0.66
	GFSv16					*GFSv16*			
	0.49	0.96	1.79	0.65		-0.23	-0.66	2.05	0.67
**Day 3**	MB (m/s)	*NMB (%)	RMSE (m/s)	R	**Day 3**	MB (m/s)	*NMB (%)	RMSE (m/s)	R
	NMMB					NMMB			
	n/a	n/a	n/a	n/a		n/a	n/a	n/a	n/a
	GFSv16					GFSv16			
	0.46	0.9	1.87	0.62		-0.26	-0.74	2.15	0.63

* NMB stats are normalized by observation range.

Table S4. Same as in Table S3, but for January 2021.

**TEMP2: January 2021-East U.S.**					**TEMP2: January 2021-West U.S.**				
**Day 1**	MB (K)	*NMB (%)	RMSE (K)	R	**Day 1**	MB (K)	*NMB (%)	RMSE (K)	R
	NMMB					*NMMB*			
	-0.03	-0.04	2.08	0.97		-0.68	-0.95	3.05	0.93
	GFSv16					*GFSv16*			
	-0.19	-0.27	2.27	0.97		-0.3	-0.41	3.33	0.92
**Day 2**	MB (K)	*NMB (%)	RMSE (K)	R	**Day 2**	MB (K)	*NMB (%)	RMSE (K)	R
	NMMB					NMMB			
	-0.16	-0.23	2.48	0.96		-0.84	-1.19	3.37	0.92
	GFSv16					*GFSv16*			
	-0.04	-0.06	2.44	0.96		-0.27	-0.36	3.45	0.91
**Day 3**	MB (K)	NMB (%)	RMSE (K)	R	**Day 3**	MB (K)	NMB (%)	RMSE (K)	R
	NMMB					*NMMB*			
	n/a	n/a	n/a	n/a		n/a	n/a	n/a	n/a
	GFSv16					*GFSv16*			
	0.14	0.19	2.6	0.95		-0.28	-0.39	3.62	0.91
**Q2: January 2021-East U.S.**					**Q2: January 2021-West U.S.**				
**Day 1**	MB (g/kg)	*NMB (%)	RMSE (g/kg)	R	**Day 1**	MB (g/kg)	*NMB (%)	RMSE (g/kg)	R
	NMMB					*NMMB*			
	0.1	0.51	0.59	0.97		0.14	0.87	0.67	0.92
	GFSv16					*GFSv16*			
	-0.05	-0.28	0.62	0.97		-0.16	-0.98	0.69	0.91
**Day 2**	MB	*NMB (%)	RMSE	R	**Day 2**	MB (g/kg)	*NMB (%)	RMSE (g/kg)	R
	NMMB					*NMMB*			
	0.06	0.3	0.68	0.96		0.11	0.71	0.73	0.89
	GFSv16					*GFSv16*			
	0.01	0.05	0.71	0.96		-0.16	-1.03	0.73	0.9
**Day 3**	MB (g/kg)	*NMB (%)	RMSE (g/kg)	R	**Day 3**	MB (g/kg)	*NMB (%)	RMSE (g/kg)	R
	NMMB					*NMMB*			
	n/a	n/a	n/a	n/a		n/a	n/a	n/a	n/a
	GFSv16					*GFSv16*			
	0.09	0.48	0.82	0.95		-0.16	-1	0.78	0.88
**WSPD10: January 2021-East U.S.**					**WSPD10: January 2021-West U.S.**				
**Day 1**	MB (m/s)	*NMB (%)	RMSE (m/s)	R	**Day 1**	MB (m/s)	*NMB (%)	RMSE (m/s)	R
	NMMB					*NMMB*			
	0.11	0.18	1.61	0.7		-0.56	-1.14	2.23	0.72
	GFSv16					*GFSv16*			
	0.13	0.22	1.66	0.7		-0.68	-1.38	2.33	0.72
**Day 2**	MB (m/s)	*NMB (%)	RMSE (m/s)	R	**Day 2**	MB (m/s)	*NMB (%)	RMSE (m/s)	R
	NMMB					*NMMB*			
	0.16	0.27	1.72	0.67		-0.53	-1.08	2.34	0.68
	GFSv16					*GFSv16*			
	0.14	0.23	1.75	0.67		-0.71	-1.44	2.43	0.69
**Day 3**	MB (m/s)	*NMB (%)	RMSE (m/s)	R	**Day 3**	MB (m/s)	*NMB (%)	RMSE (m/s)	R
	NMMB					NMMB			
	n/a	n/a	n/a	n/a		n/a	n/a	n/a	n/a
	GFSv16					GFSv16			
	0.15	0.24	1.86	0.62		-0.74	-1.48	2.59	0.65
* NMB stats are normalized by observation range.									

Table S5. Average September 2020 hourly O_3 evaluation of the operational NAQFC and NACC-CMAQ Day 2 forecasts against the AirNow network in different CONUS regions (based on https://www.epa.gov/aboutepa/regional-and-geographic-offices). Statistical benchmark values based on Emery et al. (2017) are also shown for comparison. Following Emery et al., a >40 ppb (i.e., daytime) cutoff for hourly O_3 is applied for the mean observations, mean models, mean bias, and the calculated values of NMB and NME, but not for the correlation value (r) or index of agreement (IOA). Total # of obs-model pairs are based on all values (i.e., no cutoff). **Bold** font indicates statistical values outside of the Emery et al. criteria. *Italic* font indicates improved NACC-CMAQ performance.

**Day 2 Forecasts**	**Total # of Pairs**	**Mean Obs (ppb)**	**Mean Mod (ppb)**	**Mean Bias (ppb)**	**NMB (%)**	**NME (%)**	**Corr (r)**	**IOA**
**Benchmark Emery et al. (2017)**	-	-	-	-	Goal: <±5% Criteria: <±15%	Goal: <15% Criteria: <25%	Goal: >0.75 Criteria: >0.50	-
**Region 1 (Northeast)**								
**NAQFC**	35975	46.85	42.11	-4.74	-10.12	16.69	0.59	0.70
**NACC-CMAQ**			43.14	*-3.71*	*-7.92*	*15.45*	*0.70*	*0.81*
**Region 2 (NY-NJ)**								
**NAQFC**	22920	46.63	42.37	-4.26	-9.14	17.12	0.56	0.71
**NACC-CMAQ**			44.50	*-2.14*	*-4.58*	*15.35*	*0.71*	*0.81*
**Region 3 (Mid-Atlantic)**								
**NAQFC**	88924	46.62	44.01	-2.60	-5.59	12.89	0.64	0.72
**NACC-CMAQ**			45.83	*-0.79*	*-1.69*	*13.88*	*0.74*	*0.82*
**Region 4 (Southeast)**								
**NAQFC**	105832	44.52	45.62	1.10	2.46	13.53	0.61	0.65
**NACC-CMAQ**			47.50	2.98	6.68	15.68	*0.72*	*0.75*
**Region 5 (Upper Midwest)**								
**NAQFC**	109589	46.57	43.26	-3.31	-7.10	13.86	0.67	0.77
**NACC-CMAQ**			46.57	*0.00*	*0.01*	*11.29*	*0.76*	*0.83*
**Region 6 (South)**								
**NAQFC**	83955	48.16	46.98	-1.18	-2.44	13.77	0.67	0.76
**NACC-CMAQ**			48.21	*0.05*	*0.11*	*13.37*	*0.74*	*0.81*
**Region 7 (Central Plains)**								
**NAQFC**	27120	45.10	44.63	-0.47	-1.05	10.64	0.77	0.83
**NACC-CMAQ**			47.40	2.31	5.11	*9.74*	*0.81*	*0.85*
**Region 8 (Northern Plains)**								
**NAQFC**	51728	48.97	44.52	-4.45	-9.09	14.43	0.69	0.81
**NACC-CMAQ**			*45.03*	*-3.93*	*-8.03*	*14.13*	*0.72*	*0.84*
**Region 9 (West)**								
**NAQFC**	124039	55.59	49.48	-6.11	-11.00	19.47	0.68	0.78
**NACC-CMAQ**			46.73	-8.87	**-15.95**	21.82	*0.71*	*0.83*
**Region 10 (Northwest)**								
**NAQFC**	14128	48.31	39.21	-9.10	**-18.83**	22.03	0.60	0.71

NACC-CMAQ			41.46	*-6.84*	*-14.17*	*19.86*	*0.66*	*0.81*

Table S6. Same as in Table S5, but for MDA8 O_3. Note: As discussed in Emery et al. (2017), there are no cutoff values applied for MDA8 O_3.

Day 2 Forecasts	Total # of Pairs	Mean Obs (ppb)	Mean Mod (ppb)	Mean Bias (ppb)	NMB (%)	NME (%)	Corr (r)	IOA
**Benchmark Emery et al. (2017)**	-	-	-	-	Goal: <±5% Criteria: <±15%	Goal: <15% Criteria: <25%	Goal: >0.75 Criteria: >0.50	-
				**Region 1 (Northeast)**				
**NAQFC**	1680	33.22	38.12	4.89	14.73	21.68	0.62	0.71
**NACC-CMAQ**			38.50	5.28	**15.90**	20.65	*0.73*	*0.76*
				**Region 2 (NY-NJ)**				
**NAQFC**	1160	33.13	37.37	4.24	12.81	19.95	0.66	0.75
**NACC-CMAQ**			39.03	5.90	**17.81**	23.09	*0.70*	0.75
				**Region 3 (Mid-Atlantic)**				
**NAQFC**	4250	34.05	39.43	5.38	15.81	20.52	0.72	0.76
**NACC-CMAQ**			41.25	7.19	**21.13**	23.72	*0.76*	0.76
				**Region 4 (Southeast)**				
**NAQFC**	5077	31.02	39.94	8.92	**28.75**	**30.76**	0.65	0.65
**NACC-CMAQ**			40.90	9.88	**31.84**	**33.22**	*0.71*	*0.66*
				**Region 5 (Upper Midwest)**				
**NAQFC**	5210	34.34	37.68	3.34	9.73	18.24	0.74	0.82
**NACC-CMAQ**			40.36	6.01	**17.51**	20.50	*0.80*	0.81
				**Region 6 (South)**				
**NAQFC**	3900	35.84	42.23	6.39	**17.83**	23.60	0.72	0.77
**NACC-CMAQ**			43.28	7.43	**20.74**	24.33	*0.77*	*0.78*
				**Region 7 (Central Plains)**				
**NAQFC**	1255	33.69	37.50	3.81	11.30	16.54	0.81	0.85
**NACC-CMAQ**			40.00	6.31	**18.72**	20.50	*0.84*	0.83
				**Region 8 (Northern Plains)**				
**NAQFC**	2378	44.12	43.56	-0.56	-1.27	13.23	0.73	0.84
**NACC-CMAQ**			44.91	0.79	1.79	*11.84*	*0.79*	*0.88*
				**Region 9 (West)**				
**NAQFC**	5754	51.18	50.73	-0.45	-0.88	18.85	0.67	0.79
**NACC-CMAQ**			48.60	-2.59	-5.05	*18.84*	*0.68*	0.79
				**Region 10 (Northwest)**				
**NAQFC**	695	33.14	35.62	2.49	7.51	25.12	0.61	0.70
**NACC-CMAQ**			36.71	3.58	10.80	*24.95*	0.59	*0.74*

Table S7. Same as in Table S5, but for 24-hr average $PM_{2.5}$. Note: As discussed in Emery et al. (2017), there are no cutoff values applied for 24-hr average $PM_{2.5}$.

Day 2 Forecasts	Total # of Pairs	Mean Obs (ppb)	Mean Mod (ppb)	Mean Bias (ppb)	NMB (%)	NME (%)	Corr (r)	IOA
**Benchmark Emery et al. (2017)**	-	-	-	-	Goal: <±10% Criteria: <±30%	Goal: <35% Criteria: <50%	Goal: >0.70 Criteria: >0.40	-
**Region 1 (Northeast)**								
**NAQFC**	1261	7.43	8.34	0.91	12.25	41.43	0.76	0.85
**NACC-CMAQ**			9.36	1.93	25.98	46.93	0.73	0.82
**Region 2 (NY-NJ)**								
**NAQFC**	598	8.54	14.37	5.83	**68.29**	**79.48**	0.73	0.60
**NACC-CMAQ**			10.95	*2.41*	*28.21*	*47.05*	*0.77*	*0.74*
**Region 3 (Mid-Atlantic)**								
**NAQFC**	1897	9.16	11.12	1.96	21.39	38.03	0.78	0.85
**NACC-CMAQ**			10.17	*1.01*	*10.99*	*32.62*	*0.83*	*0.89*
**Region 4 (Southeast)**								
**NAQFC**	3621	8.45	9.33	0.89	10.53	40.10	**0.39**	0.61
**NACC-CMAQ**			7.92	*-0.53*	*-6.23*	*37.61*	*0.47*	*0.66*
**Region 5 (Upper Midwest)**								
**NAQFC**	3270	9.61	8.67	-0.94	-9.74	39.45	0.49	0.70
**NACC-CMAQ**			9.41	*-0.20*	*-2.05*	*32.51*	*0.68*	*0.81*
**Region 6 (South)**								
**NAQFC**	2101	8.39	7.71	-0.68	-8.12	46.83	**0.26**	0.55
**NACC-CMAQ**			6.27	-2.12	-25.27	*44.98*	***0.33***	*0.57*
**Region 7 (Central Plains)**								
**NAQFC**	926	8.67	8.78	0.11	1.29	45.72	**0.30**	0.57
**NACC-CMAQ**			8.86	0.19	2.22	*35.69*	*0.61*	*0.77*
**Region 8 (Northern Plains)**								
**NAQFC**	1790	7.66	3.98	-3.68	**-48.01**	**62.79**	**0.29**	0.52
**NACC-CMAQ**			5.11	*-2.55*	***-33.24***	***55.73***	*0.43*	*0.64*
**Region 9 (West)**								
**NAQFC**	4118	10.09	6.77	-3.32	**-32.94**	48.56	0.59	0.72
**NACC-CMAQ**			7.81	*-2.28*	*-22.62*	**52.08**	0.54	0.72
**Region 10 (Northwest)**								
**NAQFC**	3922	7.93	6.08	-1.85	-23.31	**75.40**	**0.19**	0.47
**NACC-CMAQ**			6.02	-1.91	-24.10	***70.44***	***0.23***	*0.50*

Table S8. Same as in Table S5, but for the Day 3 forecast hourly O_3.

**Day 3 Forecasts**	**Total # of Pairs**	**Mean Obs (ppb)**	**Mean Mod (ppb)**	**Mean Bias (ppb)**	**NMB (%)**	**NME (%)**	**Corr (r)**	**IOA**
**Benchmark Emery et al. (2017)**	-	-	-	-	Goal: <±5% Criteria: <±15%	Goal: <15% Criteria: <25%	Goal: >0.75 Criteria: >0.50	-
**Region 1 (Northeast)**								
**NAQFC**	n.a.	n.a.	n.a.	n.a.	n.a.	n.a.	n.a.	n.a.
**NACC-CMAQ**	35903	46.72	43.66	-3.06	-6.55	15.97	0.68	0.79
**Region 2 (NY-NJ)**								
**NAQFC**	n.a.	n.a.	n.a.	n.a.	n.a.	n.a.	n.a.	n.a.
**NACC-CMAQ**	23116	46.60	44.53	-2.07	-4.45	14.64	0.71	0.81
**Region 3 (Mid-Atlantic)**								
**NAQFC**	n.a.	n.a.	n.a.	n.a.	n.a.	n.a.	n.a.	n.a.
**NACC-CMAQ**	88703	46.62	45.74	-0.89	-1.90	13.94	0.72	0.81
**Region 4 (Southeast)**								
**NAQFC**	n.a.	n.a.	n.a.	n.a.	n.a.	n.a.	n.a.	n.a.
**NACC-CMAQ**	105520	44.68	47.21	2.53	5.67	15.69	0.70	0.75
**Region 5 (Upper Midwest)**								
**NAQFC**	n.a.	n.a.	n.a.	n.a.	n.a.	n.a.	n.a.	n.a.
**NACC-CMAQ**	108852	46.62	46.34	-0.28	-0.60	11.48	0.74	0.82
**Region 6 (South)**								
**NAQFC**	n.a.	n.a.	n.a.	n.a.	n.a.	n.a.	n.a.	n.a.
**NACC-CMAQ**	83883	48.18	48.30	0.13	0.26	13.50	0.74	0.81
**Region 7 (Central Plains)**								
**NAQFC**	n.a.	n.a.	n.a.	n.a.	n.a.	n.a.	n.a.	n.a.
**NACC-CMAQ**	27070	45.11	46.98	1.87	4.15	11.04	0.79	0.84
**Region 8 (Northern Plains)**								
**NAQFC**	n.a.	n.a.	n.a.	n.a.	n.a.	n.a.	n.a.	n.a.
**NACC-CMAQ**	51645	48.99	44.89	-4.10	-8.36	14.04	0.71	0.84
**Region 9 (West)**								
**NAQFC**	n.a.	n.a.	n.a.	n.a.	n.a.	n.a.	n.a.	n.a.
**NACC-CMAQ**	123816	55.68	46.60	-9.09	**-16.32**	22.38	0.71	0.83
**Region 10 (Northwest)**								
**NAQFC**	n.a.	n.a.	n.a.	n.a.	n.a.	n.a.	n.a.	n.a.
**NACC-CMAQ**	14065	48.41	41.64	-6.77	-13.99	19.59	0.65	0.80

Table S9. Same as in Table S5, but for Day 3 forecast MDA8 O_3. Note: As discussed in Emery et al. (2017), there are no cutoff values applied for MDA8 O_3.

Day 3 Forecasts	Total # of Pairs	Mean Obs (ppb)	Mean Mod (ppb)	Mean Bias (ppb)	NMB (%)	NME (%)	Corr (r)	IOA
**Benchmark Emery et al. (2017)**	-	-	-	-	Goal: <±5% Criteria: <±15%	Goal: <15% Criteria: <25%	Goal: >0.75 Criteria: >0.50	-
**Region 1 (Northeast)**								
**NAQFC**	n.a.	n.a.	n.a.	n.a.	n.a.	n.a.	n.a.	n.a.
**NACC-CMAQ**	1680	33.40	39.21	5.80	**17.37**	22.09	0.68	0.73
**Region 2 (NY-NJ)**								
**NAQFC**	n.a.	n.a.	n.a.	n.a.	n.a.	n.a.	n.a.	n.a.
**NACC-CMAQ**	1161	33.11	38.83	5.72	**17.26**	21.61	0.73	0.77
**Region 3 (Mid-Atlantic)**								
**NAQFC**	n.a.	n.a.	n.a.	n.a.	n.a.	n.a.	n.a.	n.a.
**NACC-CMAQ**	4253	34.13	40.90	6.77	**19.84**	22.97	0.73	0.76
**Region 4 (Southeast)**								
**NAQFC**	n.a.	n.a.	n.a.	n.a.	n.a.	n.a.	n.a.	n.a.
**NACC-CMAQ**	5077	31.00	40.24	9.24	**29.82**	31.97	0.69	0.68
**Region 5 (Upper Midwest)**								
**NAQFC**	n.a.	n.a.	n.a.	n.a.	n.a.	n.a.	n.a.	n.a.
**NACC-CMAQ**	5159	34.50	40.54	6.04	**17.49**	20.86	0.77	0.79
**Region 6 (South)**								
**NAQFC**	n.a.	n.a.	n.a.	n.a.	n.a.	n.a.	n.a.	n.a.
**NACC-CMAQ**	3900	35.66	43.18	7.52	**21.08**	**25.10**	0.75	0.77
**Region 7 (Central Plains)**								
**NAQFC**	n.a.	n.a.	n.a.	n.a.	n.a.	n.a.	n.a.	n.a.
**NACC-CMAQ**	1256	33.64	39.69	6.05	**17.98**	20.86	0.79	0.82
**Region 8 (Northern Plains)**								
**NAQFC**	n.a.	n.a.	n.a.	n.a.	n.a.	n.a.	n.a.	n.a.
**NACC-CMAQ**	2379	44.23	44.86	0.63	1.42	11.66	0.78	0.88
**Region 9 (West)**								
**NAQFC**	n.a.	n.a.	n.a.	n.a.	n.a.	n.a.	n.a.	n.a.
**NACC-CMAQ**	5758	51.25	48.64	-2.61	-5.10	19.22	0.66	0.78
**Region 10 (Northwest)**								
**NAQFC**	n.a.	n.a.	n.a.	n.a.	n.a.	n.a.	n.a.	n.a.
**NACC-CMAQ**	697	33.22	37.03	3.81	11.46	**25.09**	0.57	0.73

Table S10. Same as in Table S5, but for Day 3 forecast 24-hr average $PM_{2.5}$. Note: As discussed in Emery et al. (2017), there are no cutoff values applied for 24-hr average $PM_{2.5}$.

**Day 3 Forecasts**	**Total # of Pairs**	**Mean Obs (ppb)**	**Mean Mod (ppb)**	**Mean Bias (ppb)**	**NMB (%)**	**NME (%)**	**Corr (r)**	**IOA**
**Benchmark Emery et al. (2017)**	-	-	-	-	Goal: <±10% Criteria: <±30%	Goal: <35% Criteria: <50%	Goal: >0.70 Criteria: >0.40	-
**Region 1 (Northeast)**								
**NAQFC**	n.a.	n.a.	n.a.	n.a.	n.a.	n.a.	n.a.	n.a.
**NACC-CMAQ**	1261	7.43	9.41	1.98	26.60	**50.07**	0.73	0.81
**Region 2 (NY-NJ)**								
**NAQFC**	n.a.	n.a.	n.a.	n.a.	n.a.	n.a.	n.a.	n.a.
**NACC-CMAQ**	598	8.54	10.80	2.26	26.42	45.71	0.79	0.78
**Region 3 (Mid-Atlantic)**								
**NAQFC**	n.a.	n.a.	n.a.	n.a.	n.a.	n.a.	n.a.	n.a.
**NACC-CMAQ**	1897	9.16	10.10	0.94	10.24	32.91	0.82	0.89
**Region 4 (Southeast)**								
**NAQFC**	n.a.	n.a.	n.a.	n.a.	n.a.	n.a.	n.a.	n.a.
**NACC-CMAQ**	3621	8.45	8.09	-0.36	-4.25	40.40	0.45	0.64
**Region 5 (Upper Midwest)**								
**NAQFC**	n.a.	n.a.	n.a.	n.a.	n.a.	n.a.	n.a.	n.a.
**NACC-CMAQ**	3270	9.61	9.23	-0.37	-3.87	34.63	0.63	0.79
**Region 6 (South)**								
**NAQFC**	n.a.	n.a.	n.a.	n.a.	n.a.	n.a.	n.a.	n.a.
**NACC-CMAQ**	2101	8.39	6.24	-2.15	-25.58	46.79	**0.31**	0.56
**Region 7 (Central Plains)**								
**NAQFC**	n.a.	n.a.	n.a.	n.a.	n.a.	n.a.	n.a.	n.a.
**NACC-CMAQ**	926	8.67	8.65	-0.02	-0.25	37.08	0.57	0.74
**Region 8 (Northern Plains)**								
**NAQFC**	n.a.	n.a.	n.a.	n.a.	n.a.	n.a.	n.a.	n.a.
**NACC-CMAQ**	1790	7.66	5.12	-2.53	**-33.09**	**55.27**	0.44	0.64
**Region 9 (West)**								
**NAQFC**	n.a.	n.a.	n.a.	n.a.	n.a.	n.a.	n.a.	n.a.
**NACC-CMAQ**	4118	10.09	7.55	-2.54	-25.15	**52.82**	0.52	0.70
**Region 10 (Northwest)**								
**NAQFC**	n.a.	n.a.	n.a.	n.a.	n.a.	n.a.	n.a.	n.a.
**NACC-CMAQ**	3922	7.93	5.90	-2.03	-25.60	**70.57**	**0.23**	0.49
